# Optimization of density fitting auxiliary Slater‐type basis functions for time‐dependent density functional theory

**DOI:** 10.1002/jcc.26992

**Published:** 2022-09-07

**Authors:** Marco Medves, Giovanna Fronzoni, Mauro Stener

**Affiliations:** ^1^ Dipartimento di Scienze Chimiche e Farmaceutiche Università di Trieste Trieste Italy

**Keywords:** density fitting, TDDFT

## Abstract

A new set of auxiliary basis function suitable to fit the induced electron density is presented. Such set has been optimized in order to furnish accurate absorption spectra using the complex polarizability algorithm of time‐dependent density functional theory (TDDFT). An automatic procedure has been set up, able, thanks to the definition of suitable descriptors, to evaluate the resemblance of the auxiliary basis‐dependent calculated spectra with respect to a reference. In this way, it has been possible to reduce the size of the basis set maximizing the basis set accuracy. Thanks to the choice to employ a collection of molecules for each element, such basis has proven transferable to molecules outside the collection. The final sets are therefore much more accurate and smaller than the previously optimized ones and have been already included in the database of the last release of the AMS suite of programs. The availability of the present new set will allow to improve drastically the applicability range of the polTDDFT method with higher accuracy and less computational effort.

## Graphical abstract

A new set of auxiliary basis function suitable to fit the induced electron density is presented. Such set has been optimized in order to furnish accurate absorption spectra using the complex polarizability algorithm of time‐dependent density functional theory (TDDFT). An automatic procedure has been set up, able, thanks to the definition of suitable descriptors, to evaluate the resemblance of the auxiliary basis dependent calculated spectra with respect to a reference. The value of the descriptor allows to give a quantitative measure of the goodness of the basis set and is employed to follow it during the reduction and optimization procedures.

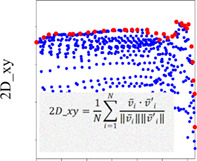

## 1 INTRODUCTION

The present study has been promoted by the recent new time‐dependent density functional theory (TDDFT) algorithm,^1^ whose implementation within the Amsterdam Density Functional‐Amsterdam Modeling Suite (ADF‐AMS) program using slater‐type orbitals (STO) basis sets^2^ has proven very efficient to treat very large systems.^3^, ^4^ Such algorithm, which consists to extract the photoabsorption spectrum from the imaginary part of the complex polarizability, will be referred as the polTDDFT algorithm in the following. The great advantage of polTDDFT consists to avoid the diagonalization (as in the typical Casida implementation of quantum chemistry^5^), in fact, the spectrum is calculated point‐by‐point solving a linear system of equations at each photon energy. The unknown term of the linear system is the induced density, so it is quite natural to represent such an equation over a basis set of density fitting functions. In this way the matrix dimension remains much smaller than in Casida or in random phase approximation (RPA) where the size of the matrix to be diagonalized has a dimension equal to the product between the number of occupied and virtual orbitals. On the other hand, in order to employ in practice the polTDDFT method, it is necessary to have available the auxiliary density‐fitting basis set, which must be optimized for polTDDFT calculation. In fact, it has been found that the standard density fitting auxiliary STO basis set included in the ADF program, which is employed to fit the Self Consistent Field (SCF) electron density for the calculation of the coulomb term of the Kohn‐Sham Hamiltonian matrix, is too large for polTDDFT.^1^ It is too large because it was developed to fit the electron density from deep core to valence orbitals, while in polTDDFT usually we need only a limited energy interval, typically up to 10 eV at most. In fact, one is usually interested in the optical region of photoabsorption, so it is not necessary to include in the density fitting set functions, which describe core electron density. Up to now, polTDDFT optimized fitting set were available only for few elements,^1^ with present work we have generated a database to be employed for polTDDFT calculations of the whole periodic table of the elements, with the exception of lanthanides and actinides.

The optimization of basis functions is a well‐consolidated problem in quantum chemistry, although it has been recently found, especially for solid‐state applications, that the “system‐specific” basis can be much more accurate than the “all‐purposes” ones.^6^ The optimization of the basis set is usually performed variationally and this ensures that the resulting basis set will be optimized for the total energy. Therefore, other properties may have more stringent basis set requirements to obtain convergent results.^7^, ^8^ Similar situations happen for density fitting auxiliary basis set: they are usually employed to fit the SCF density for the efficient calculation of the coulomb term. For such task, it is possible to employ a variational scheme^9^ or a “pair fitting” scheme as in ADF^10^ or with a partitioning technique as in AMS.^11^ Pair fitting is quite appealing since it consists in splitting the exact density in two‐center contributions and in fitting separately each pair: this is very efficient and numerically stable, since the problem is recast into a Cholesky decomposition. This allows to employ very large density fitting auxiliary basis functions without incurring in numerical instabilities and gaining high accuracy in coulomb integrals. Unfortunately, this density fitting set cannot be directly employed in polTDDFT algorithm: it is suitable for pair fitting but it is not suitable to fit the induced density for the whole molecules. In fact, the polTDDFT algorithm is numerically less stable than Cholesky decomposition therefore the auxiliary basis must be in any case heavily reduced. It is worth noting that the optimization of the density fitting functions to gain accurate TDDFT spectra has been already considered, for example to realize efficient matrix–vector products avoiding the construction of the omega Casida matrix.^12^ More recently it has been surprisingly found that for bare silver clusters a “minimal basis” density fitting set consisting of only one *s* orbital per atom is enough accurate to give quantitative match with respect to reference TDDFT.^13^

It is worth noting that auxiliary Gaussian‐Hertmite functions (named GEN‐An GEN‐An*)^14^ have proven more general as fitting functions, in fact the same set can be used to fit: in SCF both the Coulomb potential and the Fock nonlocal exchange when hybrid functionals are employed, in TDDFT, the perturbed density.^15^ At variance, STO functions seem to have quite different requirements depending on the object to be fitted, this should likely ascribed to the cusp at the origin which is retained when the STO products are performed, while for Gaussian the cusp absence, although being unphysical, makes the fitting much easier.

Another important issue concerns the optimization method: while it is natural to assume a variational criterion to fit the coulomb potential, it is much less natural to find a method to optimize the density fitting set in order to reproduce at best the photoabsorption polTDDFT spectrum. Several attempts in this direction can be found in the literature.^16^ More recently new techniques based on artificial intelligence (AI), like artificial neural networks (ANN), have proven very suitable for basis set optimization, in particular to identify a descriptor defining the resemblance of an approximate spectrum with respect to a reference one.^17^

Also in the present work, we have employed two different descriptors in order to define how much “resemblance”’ there is between a calculated spectrum and a reference spectrum, which we assume, by definition, correct. Therefore, the quality of a given auxiliary basis set is simply given by the value of the adopted descriptor, and the optimization process consists to find the auxiliary basis set which maximizes such descriptor.

The present work is organized as follows: first a short review of the polTDDFT and the Casida algorithms is given in order to stress the role of the density fitting auxiliary basis set, second an efficient method to reduce the size of the large density fitting set already available in the ADF‐AMS database is described. Third, a procedure to optimize the exponents of the reduced fit is also proposed. The size reduction of the basis set is then applied to all the elements of the periodic table (except the *f*‐blocks), while the optimization of the exponents has been applied only to a selected subset of elements. Finally a description of the database generate for the AMS program is presented.

## 2 THEORETICAL METHODS AND PROCEDURES

### 2.1 The polTDDFT method

The polTDDFT algorithm consists in an alternative method to solve the TDDFT equations. It is convenient to start with standard first‐order linear response TDDFT equations^1^:

(1) $$\left\{\;\begin{array}{c} \rho_{z}^{\left(1\right)}\left(\omega ,\bar{r}\right)=\int \chi_{\mathrm{KS}}\left(\omega ,\bar{r},{\bar{r}}^{\prime }\right)V_{\mathrm{SCF}}^{z}\left(\omega ,{\bar{r}}^{\prime }\right)d{\bar{r}}^{\prime }\\ V_{\mathrm{SCF}}^{z}\left(\omega ,\bar{r}\right)=V_{\mathrm{EXT}}^{z}\left(\omega ,\bar{r}\right)+\int \frac{\rho_{z}^{\left(1\right)}\left(\omega ,{\bar{r}}^{\prime }\right)}{\left|\bar{r}-{\bar{r}}^{\prime }\right|}d{\bar{r}}^{\prime }+\frac{\partial V_{\mathrm{XC}}}{\partial \rho }\left|\rho_{0}\rho_{z}^{\left(1\right)}\left(\omega ,\bar{r}\right)\right. \end{array}\right.$$

In Equation (1), $\rho_{z}^{\left(1\right)}\left(\omega ,\bar{r}\right)\;$ refers to the Fourier component of *ω* frequency of the time‐dependent first‐order density induced by the external potential polarized along the *z* direction and $\chi_{\mathrm{KS}}\left(\omega ,\bar{r},{\bar{r}}^{\prime }\right)$ is the dielectric susceptibility of the Kohn–Sham noninteracting systems. $V_{\mathrm{SCF}}^{z}\left(\omega ,{\bar{r}}^{\prime }\right)$ is the sum of three terms. The first one is the external potential (in present case, only the dipole field is considered), the second one is the Coulomb response of the system to the induced density (i.e., the electrostatic field generated by the induced density) finally the third term is the XC response, already approximated at the adiabatic local density approximation (ALDA) level.^18^

It is formally possible to solve the above system with respect to the first‐order density:

(2) $$\left(1-\chi_{\mathrm{KS}}K\right)\rho_{z}^{\left(1\right)}=\chi_{\mathrm{KS}}V_{\mathrm{EXT}}^{z}$$

where in Equation (2), *K* is the response kernel (sum of Coulomb and XC kernels, corresponding to the second and third terms of the right‐hand side of the second equation of system, respectively, (1)). If we now represent Equation (2) within a basis set to expand the induced density, the following nonhomogeneous linear system is obtained.

(3) $$\left[S-M\left(\omega \right)\right]b=d$$

where in Equation (3), *S* is the overlap matrix between density fitting functions, *M* is the matrix representation of the *χ*_KS_ *K* operator, *b* is the coefficients vector of the induced density (see next Expression 4), and *d* is the vector of the scalar products between the density fitting basis functions and the right‐hand side of Equation (2). In practice by solving the linear system (3), we obtain the vector *b*, which contains the coefficients of expansion of the induced density:

(4) $$\rho_{z}^{\left(1\right)}\left(\omega ,\bar{r}\right)=\sum_{\mu }f_{\mu }\left(\bar{r}\right)b_{\mu }\left(\omega \right)$$

In Expression (4), $f_{\mu }\left(\bar{r}\right)$ are the auxiliary basis functions employed to represent the induced density. Once the linear system (3) is solved, it is possible to calculate the dynamical polarizability tensor:

(5) $$\alpha_{\mathrm{zz}}\left(\omega \right)=\int \rho_{z}^{\left(1\right)}\left(\omega ,\bar{r}\right)\mathrm{zd}\bar{r}$$

as well as the photoabsorption spectrum:

(6) σω=4πωcImαω

where in Expression (6), *σ* refers to the absolute photoabsorption. The photoabsorption spectrum is therefore calculated point by point, with a scan on the excitation energy (step of 0.02 eV) and employing a small but still finite imaginary photon energy. Such imaginary energy corresponds to the reciprocal lifetime of the excited state and makes the calculated spectrum intrinsically broadened by a lorentzian function with half‐width‐half‐maximum (HWHM) equal to the imaginary photon energy 0.075 eV.

Such spectrum can be also obtained in terms of oscillator strengths:

(7) fωr=2ωiωr3Imαω

Expression (7) is quite useful since it can be directly compared with photoabsorption spectra obtained as discrete lines, provided the last ones are broadened by lorentzian functions with HWHM equal to the imaginary frequency (reciprocal lifetime) employed in the polTDDFT.^1^

It is worth noting that in polTDDFT calculations, we must specify two different basis sets: first, the standard basis set employed to expand the KS orbitals, second the auxiliary basis set to expand the induced density. To avoid any confusion we will refer to former as the “standard basis” and to the latter as the “auxiliary basis.” In fact, to build the *M* matrix and the *d* vector in the linear system (3), we need the KS orbitals and their energies (eigenvalues) which are taken from a preliminary DFT KS calculation, employing a “standard basis” of STO of TZP type and the B3LYP XC hybrid functional.^19^, ^20^ The practice goal of the present study is to generate an optimized set of “auxiliary basis” $f_{\mu }\left(\bar{r}\right)$, and we require that such set would be a good compromise between accuracy and computational economy. This means that we try to minimize the number of elements of the ‘auxiliary basis’ but we must also maximize its goodness. In order to maximize the goodness we must select a standard reference spectrum, which we assume to be error‐free and then define (with some degree of arbitrariness) a degree of “resemblance” between the polTDDFT spectrum and the standard one. Then the auxiliary basis set is changed in order to maximize the “resemblance” and minimize the basis size.

We conclude this section with some remarks regarding the efficiency of the polTDDFT with respect to the Casida scheme. For example, a calculation on the metal cluster Au_28_(SC_6_H_5_)_20_ consisting of 268 atoms at B3LYP level with HDA approximation takes 1.8 h to calculate the integrals, 2.55 h to calculate the HDA corrections and 50 min to solve the linear system 250 times (to build the spectrum up to 5 eV with energy step of 0.02 eV). This timing has been obtained using 24 cores on a HPE ProLiant ML350 Gen9 server with processor Intel® Xeon® CPU E5‐2650 v3 @ 2.30 GHz. The same calculation by Casida would have requested to extract almost 1000 roots and would be not practicable because of timing but also of numerical issues due to the too high number of eigenvalues to extract. When the system becomes larger and larger or a wider portion of the spectrum is requested, the advantages of polTDDFT with respect to Casida are even more pronounced.

### 2.2 The reference Casida TDDFT method

As we have pointed out in previous section, in order to optimize the auxiliary basis with polTDDFT, we must define a reference spectrum. It is quite natural to choose the Casida method^7^ to obtain the reference spectrum, in fact, in this case, it is possible to employ exactly the same numerical choice such as the standard basis and the XC functional. The Casida TDDFT implementation consists in solving the following eigenvalue equation:

(8) $$\Omega F=\omega^{2}F$$

It is worth noting that the dimension of the Ω matrix corresponds to the product of the number of occupied orbitals times the number of virtual orbitals and becomes rapidly very large. Therefore, the Davidson algorithm is usually employed to extract at least the lowest part of the excitation spectrum in terms of discrete lines with specific energy and intensity (oscillator strength). In order to obtain a spectrum that can be consistently compared with that obtained with the polTDDFT method, it is necessary to broaden the discrete lines with a lorentzian function, whose width has been discussed previously. In practice the following broadening is performed:

(9) $$f\left(\omega \right)=\sum_{I}^{N}\frac{\eta^{2}f_{I}}{{\left(\omega -\omega_{I}\right)}^{2}+\eta^{2}}$$

where in Expression (9), $\omega_{I}$ and $f_{I}$ are the energies and the oscillator strenghts of the *I*th discrete line, respectively, while η is the HWHM. With this representation of the spectrum, in presence of only one discrete line, we obtain a lorentzian function centered at the excitation energy having the maximum corresponding to the oscillator strength.^1^

### 2.3 The quality descriptors

The choice of the descriptors is somehow arbitrary, in fact, the deviations of a spectrum with respect to the reference one may weight different aspects, for example the energy of the spectral features, their intensity, the area under the absorption band and so on. In order to define the descriptors, it is convenient to arrange the polTDDFT spectrum, which consists in a set of *N* pairs (*E*_*i*_ *, f*_*i*_) (energy and oscillator strength, respectively), in a set of *N* two‐dimensional vectors ${\bar{v}}_{i}=\left(E_{i},f_{i}\right)=\left(x_{i},y_{i}\right)$. The same can be done for the reference Casida spectrum, in this case, we designate the vectors as $\bar{v}\prime_{i}=\left({E_{i}}^{\prime },{f_{i}}^{\prime }\right)=\left({x_{i}}^{\prime },{y_{i}}^{\prime }\right)$. The first descriptor considered (named 2D_*xy*) has been inspired by the cosine similarity (CS),^21^ which is a typical measure of similarity in data analysis:

$$\text{cosine similarity}=\frac{\bar{v}∙\bar{v\prime }}{\left\|\bar{v}\right\|\left\|\bar{v\prime }\right\|}.$$

$$2D\_\mathrm{xy}=\frac{1}{N}\sum_{i=1}^{N}\frac{{\bar{v}}_{i}∙\bar{v}\prime_{i}}{\left\|{\bar{v}}_{i}\right\|\left\|\bar{v}\prime_{i}\right\|}$$

The Euclidean norm $\left\|{\bar{v}}_{i}\right\|=\sqrt{x_{i}^{2}+y_{i}^{2}}$ is adopted in this work. Such 2D_*xy* descriptor takes the value 1 for perfect match and 0 for absence of match (orthogonal vectors). This descriptor is defined for a single spectrum, if a collection of spectra is considered then the descriptor of the collection is defined as the arithmetic mean of the descriptors relative to the single spectra.

Since it is useful to consider more descriptors, in order to check that the results are robust enough, we introduce another descriptor designed as *n*D_*y*:

$$nD\_y=\frac{\bar{y}∙\bar{y^{\prime }}}{\left\|\bar{y}\right\|\left\|\bar{y\prime }\right\|}$$

where the vector $\bar{y}=\left(f_{i}\right)=\left(y_{i}\right)$ contains only the polTDDFT intensities and ${\bar{y}}^{\prime }=\left({f_{i}}^{\prime }\right)=\left({y_{i}}^{\prime }\right)$ contains only the Casida intensities.

This second descriptor is less stringent for the intensity: in fact in presence of a spectrum which has the same shape of the reference one but is just rescaled with respect to the intensity, a perfect match is obtained since *n*D_*y* = 1.

### 2.4 Procedure to optimally reduce the auxiliary basis set

**SCHEME 1:**
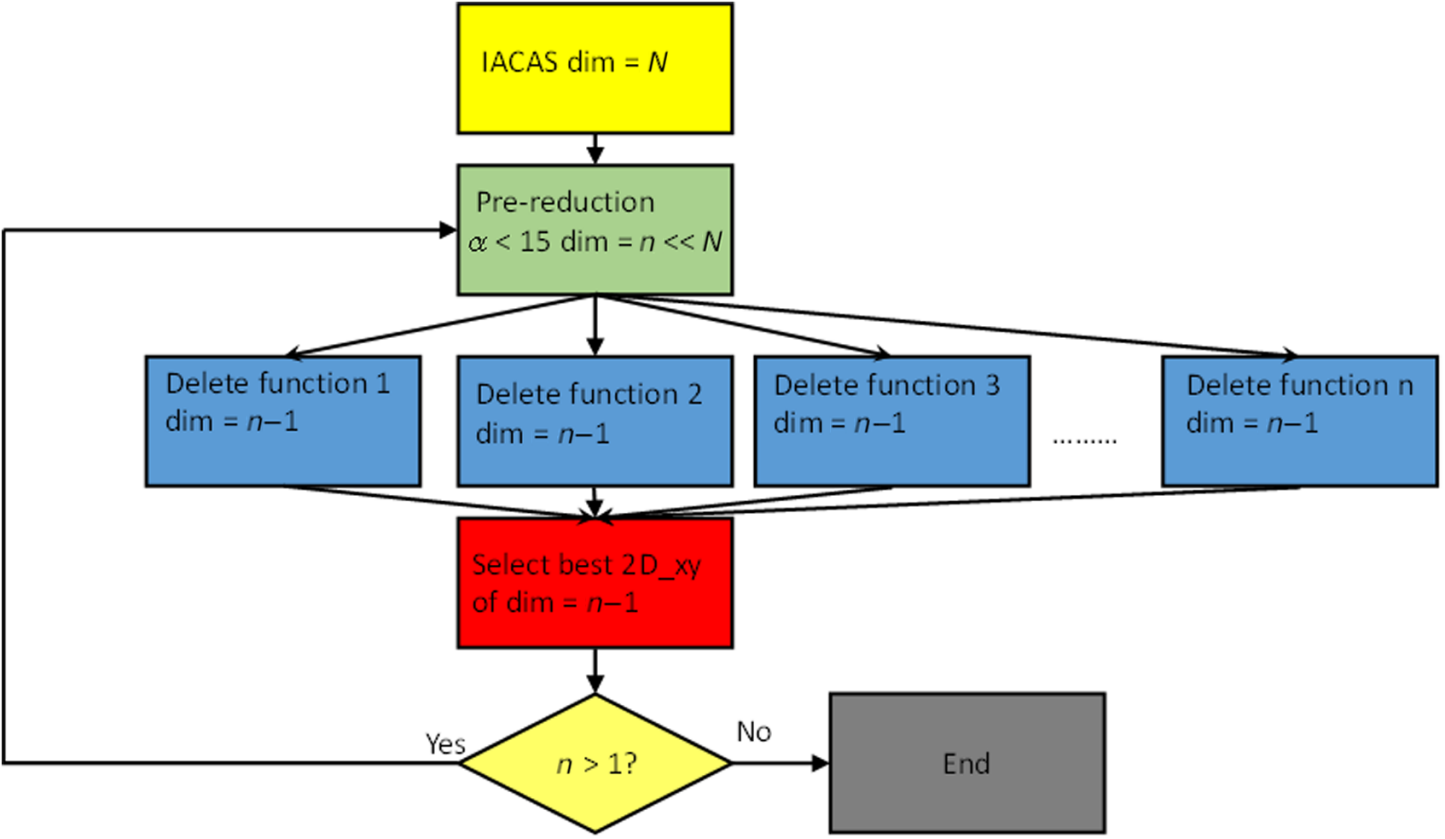
Flow chart relative to the procedure to reduce the auxiliary basis set

**FIGURE 1:**
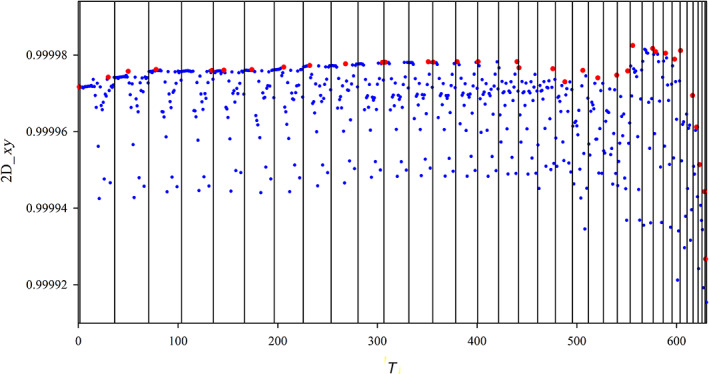
Evolution of the 2D_*xy* descriptor during the automated reduction of the auxiliary basis set for the Sn element

In the previous section, we have defined the reference spectrum and two possible measures of the quality of the approximated polTDDFT spectrum (2D_*xy* and *n*D_*y* descriptors). The next step consists to assess a practical procedure to identify an auxiliary basis which is expected to be an optimal compromise between the auxiliary basis of minimum size and the descriptor being as close as possible to +1 (best quality). All the following steps are graphically considered in the flow chart Scheme 1. It is worth noting that a very large auxiliary basis set is already available within the database included in the AMS package, but such set is not useful for polTDDFT calculations since it is far too large. We designate such set as the initial almost complete auxiliary set (IACAS) represented by the yellow box in Scheme 1. In fact, such set was specifically designed to fit the electron density of each atom pairs (“pair fitting”) in order to obtain the electrostatic potential of the Hartree term of the Kohn–Sham Hamiltonian. This set must be very large, virtually complete, in order to get the required numerical accuracy, but it can be safely employed since a Cholesky decomposition is performed to fit the pair density, which is numerically very stable. On the other hand, for polTDDFT, a too large auxiliary basis might give rise to problems of numerical linear dependence. For these reasons, our “target” auxiliary basis set is required to be small not only to be computationally cheap but also to safely avoid numerical instabilities. Therefore, we have considered first a procedure consisting to reduce systematically the number of auxiliary basis function starting from the IACAS, which is already available. It is worth noting that the IACAS contains functions to fit the electron density from the compact deep core orbitals to the more diffuse outer valence, while for polTDDFT we need only to perturb the outer valence orbitals. To this purpose a prereduction is preliminary done on the IACAS just deleting all the STO having an exponent larger than 15. This simple procedure (green box in Scheme 1) has proven useful in order to start with a set containing a much lower number of auxiliary functions having canceled all those needed to fit the core electron density. Moreover, the so‐obtained auxiliary set, although still too large, does not usually suffer of the numerical instability problems so it can be employed to run polTDDFT calculations. In some circumstances, such set was still too rich and it was not possible to run the polTDDFT calculation due to numerical linear dependence problems. In that case, we simply further reduced the set doing a diagonalization of the overlap matrix and then deleting the basis element with higher contribution in the eigenvector with minimum eigenvalue. This procedure can be repeated until the set is suitable for the polTDDFT calculation. At this point, we have set up an automatic procedure, which, starting with the pre‐reduced IACAS consisting of *n* auxiliary basis function, calculates *n* polTDDFT spectra, one for each basis set consisting of *n* − 1 elements, obtained deleting 1 basis function from the original set (blues boxes in Scheme 1). For every polTDDFT spectrum, the 2D_*xy* descriptor is calculated, and in this way the best set consisting of *n* − 1 auxiliary functions is chosen as the set giving the best 2D_*xy* descriptor (closest to 1), as reported in the red box of Scheme 1. This step can be iterated *n* times, until the set is reduced to one single function. In this way, we can associate a descriptor to each basis set of decreasing size. The evolution of the descriptor can be profitably described as reported in Figure 1. The *T* index reported on the *x* axis corresponds to each different set considered: the vertical straight lines corresponds to a jump to a basis set with one less function. Within the rectangles, there are many blue dots, which correspond to the different basis, the best basis (the highest) is designated in red. So when we jump to the next rectangle, we start from the best basis of the previous one, and again all the basis consisting of *n* − 1 elements are tested (blue dots) and the best one (red dot) is kept for the next step. It is interesting to note that within a rectangle the blue dots are distributed over a rather wide descriptor range, indicating that some basis elements are crucial to obtain accurate spectra and their suppression introduces a strong deterioration in the calculated spectrum.

**FIGURE 2:**
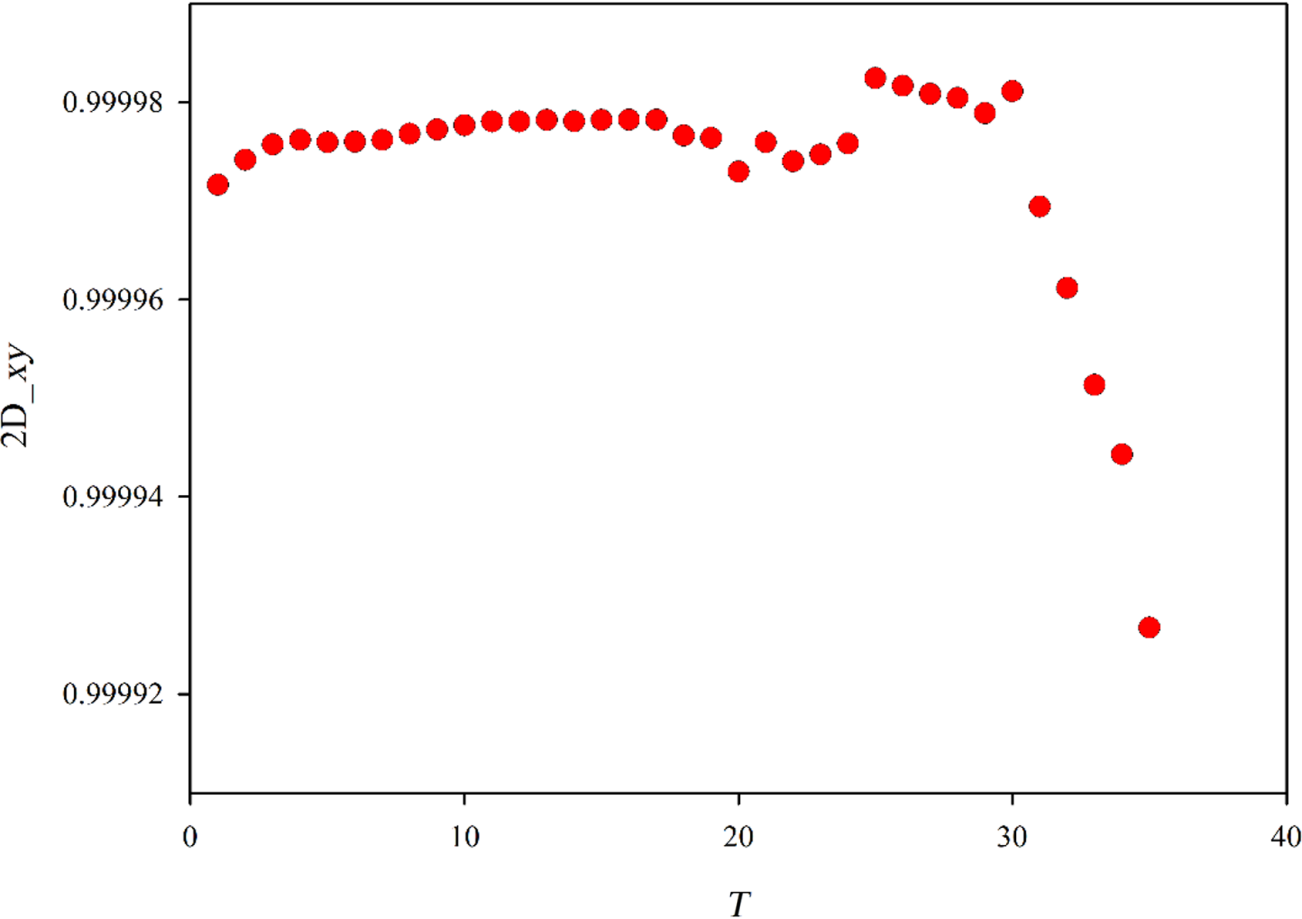
Evolution of the best 2D_*xy* descriptor along the reduction of the auxiliary basis set for the Sn element

In order to better appreciate the evolution of the best set, in Figure 2, we have reported only the red dots, which correspond to the best set of a give size. It is worth noting that up to *T* = 30, we observe a very slight improvement reducing the basis size (as *T* increases) which at the beginning is regular, but for *T* beyond 20, the behavior starts to be irregular although still increasing as average. Beyond *T* = 30, a sudden and fast regular deterioration is found. The observed behavior indicates clearly that the reduction of the basis set up to *T* = 30 does not decrease the accuracy, but beyond this point a rapid loose of accuracy is evident. At this point, the chosen optimal auxiliary basis is the one just before the sudden accuracy drop: this criterion allows to choose the basis having the smallest possible size but giving an accuracy comparable with that of larger sets. It is worth noting that the behavior reported in Figures 1 and 2 refers to the Sn element, but it is completely general, in fact it has been found in all the elements of the periodic table considered in the present work. Another very important point consists in the choice of the descriptor employed to reduce the basis set. Although in general, the descriptors are consistent each other (they usually identify the same function to be deleted) in cases where more than one function have very similar descriptors the outcome can be descriptor dependent. Since the 2D_*xy* descriptor has proven more stringent, we have used this one for the reduction process. Instead, in order to choose the best auxiliary basis, both descriptors have been checked, in order to take the safer choice. In practice, we have calculated both descriptors for the reduction series and the basis is selected so that both descriptors are maximized with the basis as small as possible.

A very important issue consists in the transferability of the obtained basis set: we must guarantee that the optimized basis for a given element using a specific molecule as a reference can be employed in other systems containing such element. This is not trivial at all, in fact from preliminary tests, we found that if only one molecule is taken as reference the obtained set is not transferable in general. This problem can be solved taking a collection of *k* molecules containing the same element as reference: so a set of *k* reference spectra will be defined and a set of different *k* descriptors will be obtained for each auxiliary basis. Then a “collection” descriptor can be defined taking the arithmetic mean of the *k* ones, and the same procedure can be applied to optimize the auxiliary basis over the collection. The so obtained auxiliary basis has proven transferable, in fact, we always checked this by comparing a polTDDFT and a Casida spectrum on a molecule outside the collection and we always got an acceptable match. In order to obtain a transferable auxiliary basis set, it is important that the collection contains the same elements in various chemical environment, but mainly as it concerns the oxidation state and the coordination number. From a computational point of view, the procedure is quite cheap: taking a collection of four molecules consisting of few tens of atoms, the optimal reduced basis takes a couple of days using around 20 cores on a HPE ProLiant ML350 Gen9 server with processor Intel® Xeon® CPU E5‐2650 v3 @ 2.30 GHz.

In summary, as an example, Table 1 reports the original IACAS auxiliary set, the pre‐reduction operated by cutting the *α* > 15 exponents, and the final reduced set (bold functions and exponents) for the Sn atom. The effect on size is dramatic, going from 57 to 6 different functions. If we take into account the angular momentum multiplicity 2 *L* + 1, the reduction is similar, going from 209 to 20 basis elements.

**TABLE 1:** The original density fitting auxiliary basis set for Sn atom

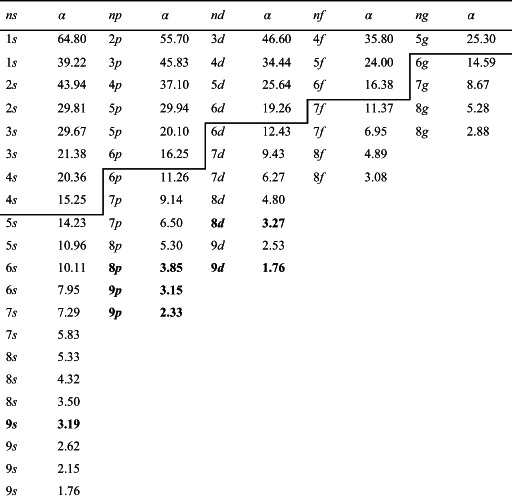

*Note*: The broken line refers to the pre‐reduction of the exponents with *α* > 15. The final optimally reduced basis elements are in bold.

### 2.5 Procedure to refine the auxiliary basis set

**SCHEME 2:**
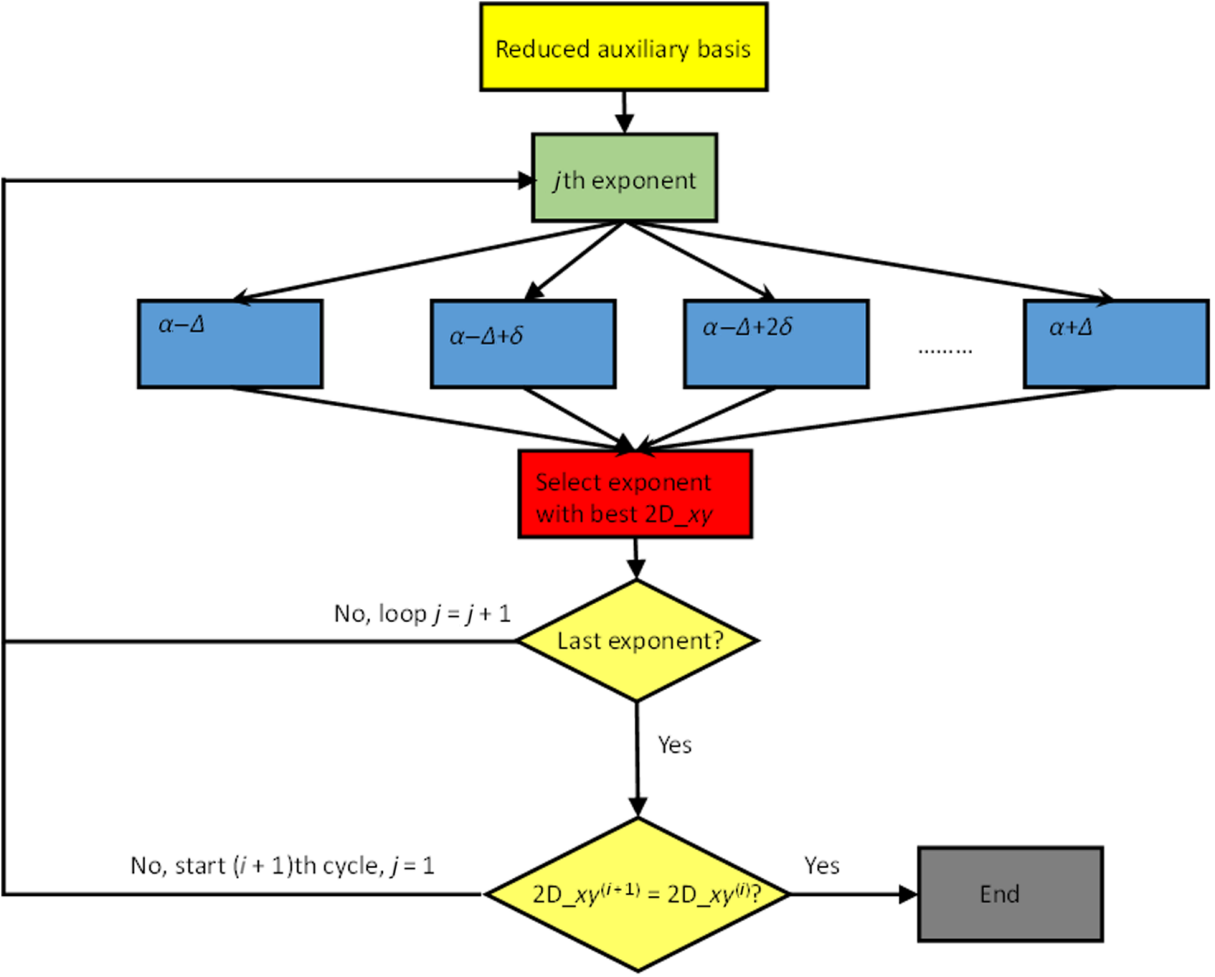
Flow chart relative to the procedure to optimize the exponents of the auxiliary basis set

In the previous section, we have described how to select the most important auxiliary basis elements starting from an initial very large set, the IACAS, so in the procedure the STO exponent were kept constant. Now we want to optimize the exponents in order to obtain an even better basis set, the procedure is described in the following and is graphically described in Scheme 2. The start is from the reduced set obtained in the previous section (upper yellow box in Scheme 2). Then, the exponent of each function is optimized separately: the exponent of the first function is varied over a set of values (blues boxes), the potTDDFT spectra are calculated and the 2D_*xy* descriptor is calculated. The exponent giving the best descriptor is then chosen (red box), and the next exponent is optimized (inner loop), using the optimized value of the previous exponent. This procedure is repeate until all the exponents are optimized. Since each exponent is optimized independently of the other ones, the cycle is repeated again on all the exponents until convergence is reached (outer loop), typically, only 3 or 4 cycles are necessary. The procedure is performed on the same collection of molecules employed in the previous section. In order to increase the efficiency, the procedure is split in two successive steps: in the first step, the basis is optimized only on the first decimal digit; in the second step also, the second digit is optimized. So in the first step the exponent is varied on an interval, which is wider, but with steps of 0.1; then in the second step, the interval is narrower but a step of 0.01 is employed. The interval wideness and step size are indicated by Δ and *δ*, respectively, in the blue boxes of Scheme 2. Such optimization procedure is much more demanding than the simpler reduction, requiring about one order of magnitude more of computer time, typically 2 or 3 weeks using 20 cpu on the same server. However, we have found that for most of the elements the reduction is enough to get a basis of good accuracy, so we have applied the exponent optimization only for few elements which were less easy to optimize or for which we required a special accuracy.

### 2.6 Computational details

In all calculations, the basis set for the expansion of the molecular Kohn–Sham orbitals has been taken from the AMS database of STO functions. The accurate triple zeta plus polarization (TZP) set has been chosen. For the geometry optimization of the molecules considered in the collection, the exchange correlation energy functional has been approximated at the local density approximation (LDA) with the VWN parametrization.^22^ In order to have accurate spectra calculations, we have considered in both Casida and polTDDFT calculations the B3LYP hybrid energy functional for both SCF and response part. In the response part, the nonlocal exchange of the kernel has been approximated at the hybrid diagonal approximation (HDA level) in order to save computer time without loss of accuracy.^23^ Since we have considered also heavy elements, we have included relativistic effects at the ZORA level.^24^

## 3 RESULTS AND DISCUSSION

### 3.1 Reduction of the auxiliary basis set

**FIGURE 3:**
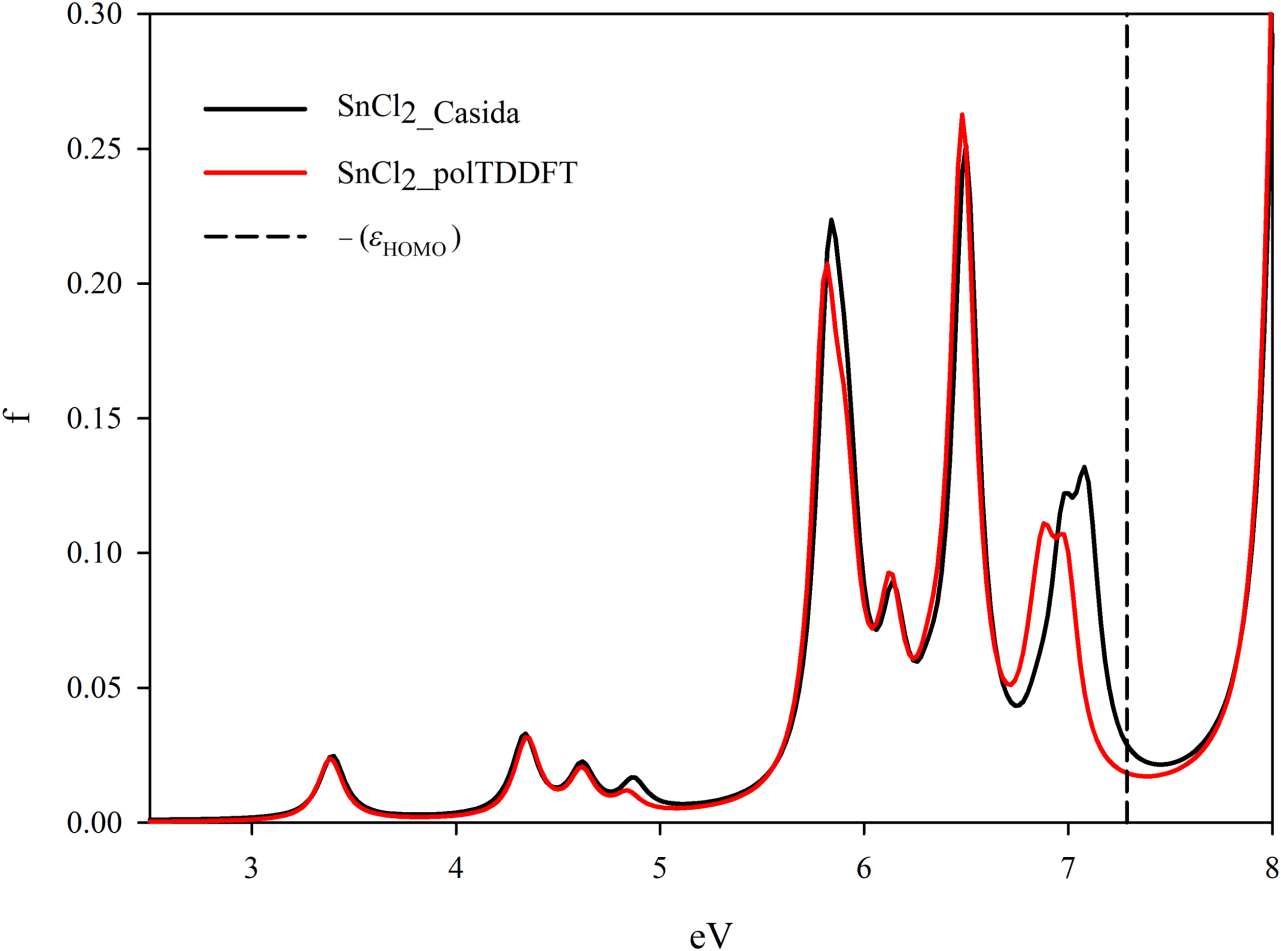
Comparison between reference (Casida) and polTDDFT spectra with reduced basis set for SnCl_2_, which belongs to the Sn collection

**FIGURE 4:**
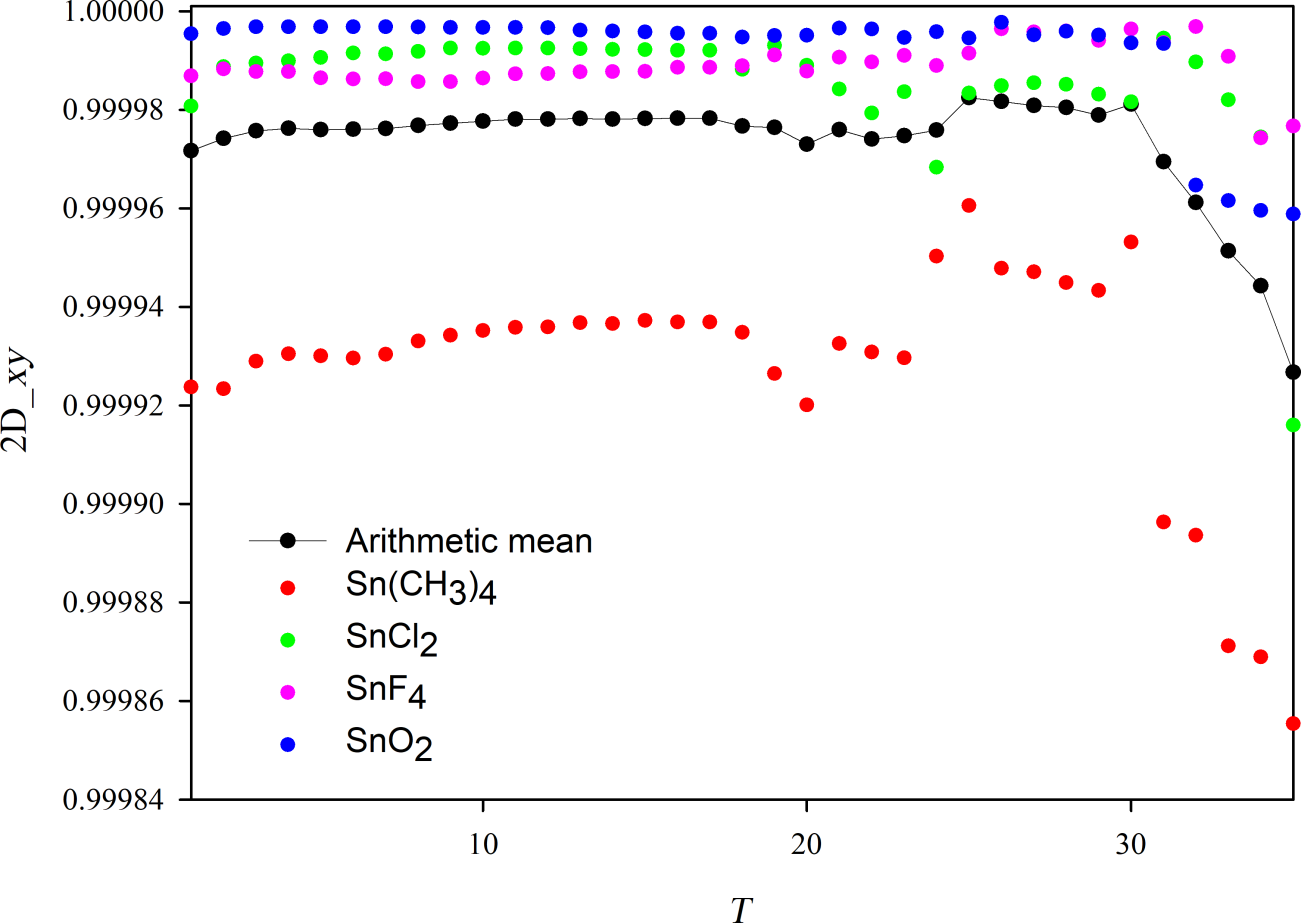
Evolution of the best 2D_*xy* descriptor along the reduction of the auxiliary basis set for the Sn element for each molecule of the collection. The arithmetic mean is reported as well.

**FIGURE 5:**
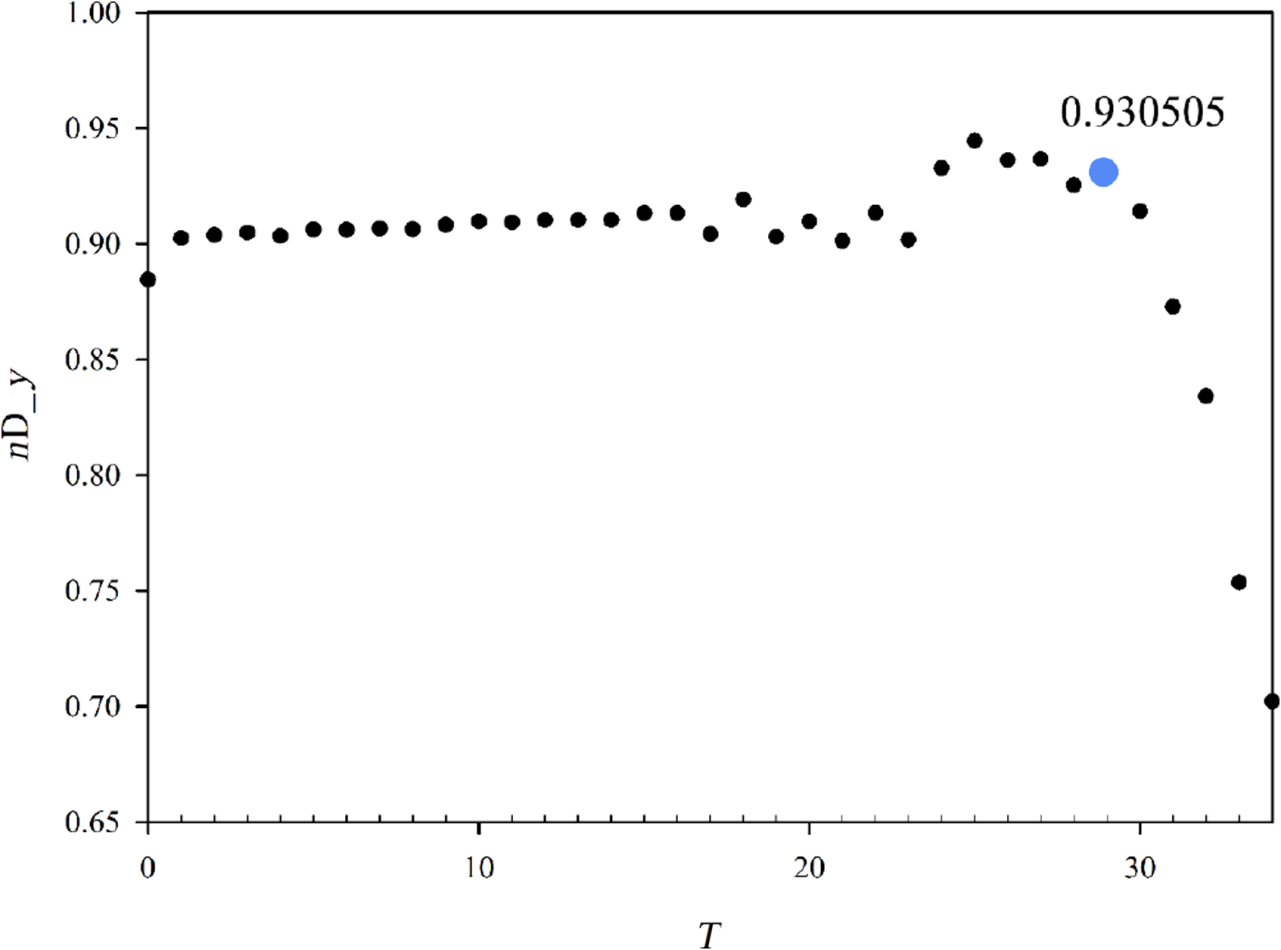
Evolution of the *n*D_*y* descriptor along the best 2D_*xy* reduced series of the auxiliary basis set for the Sn element

**FIGURE 6:**
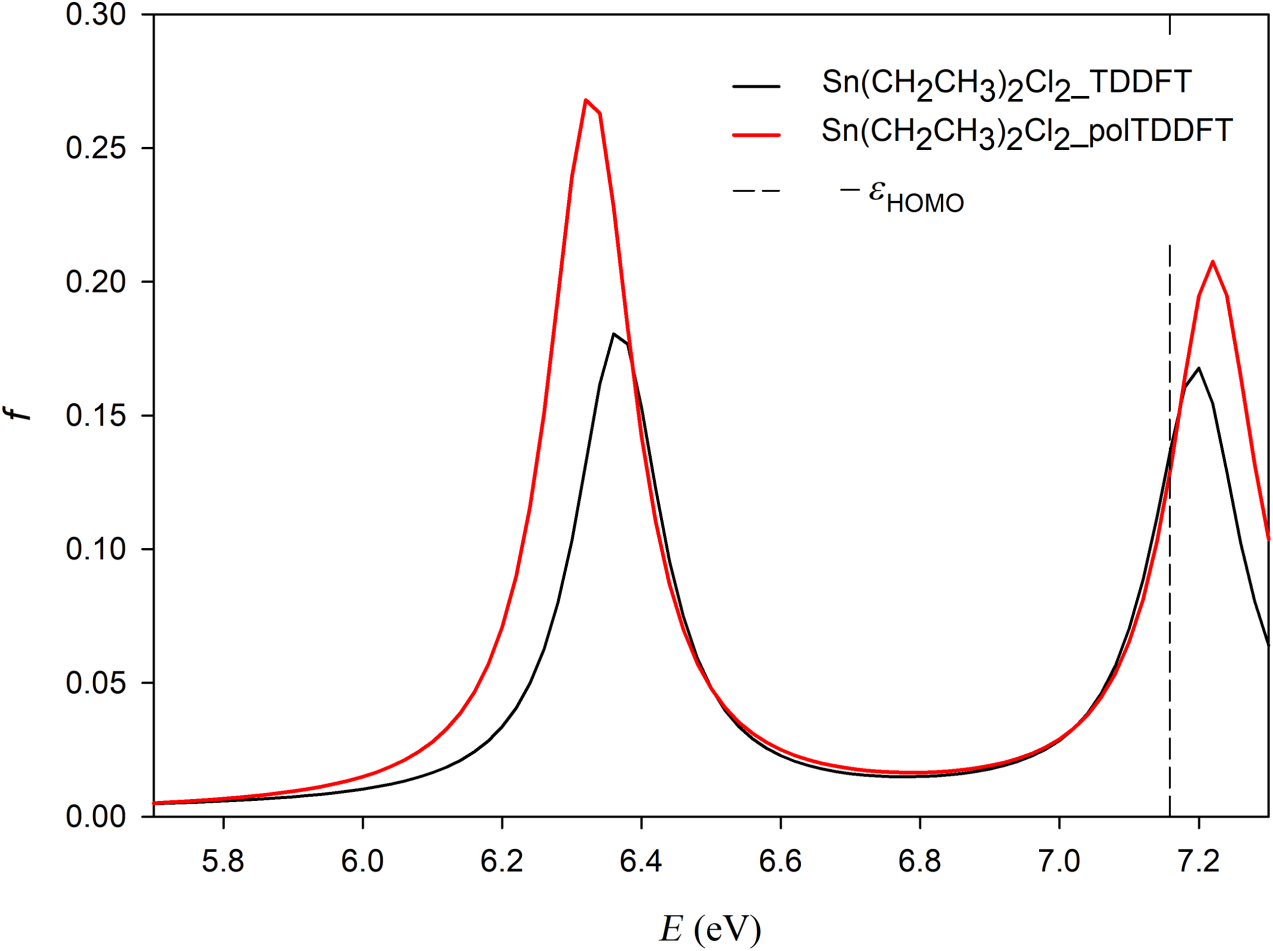
Comparison between reference (Casida) and polTDDFT spectra with reduced basis set for Sn(CH_2_CH_3_)_2_Cl_2_, which does not belong to the Sn collection

In order to describe how to realize the procedure previously described, we have selected one element of the period table (Sn) as a typical example. For the collection we chose the following 4 molecules: SnO_2_, SnCl_2_, SnF_4_, and Sn(CH_3_)_4_, in order to have two oxidation states (+2 and +4) as well as two coordination numbers (2 and 4). The geometries have been optimized and are reported in Data S1. In Table 1, we have reported the IACAS of Sn atom, which initially consists of 57 STO, we have highlighted the 22 exponents *α* > 15, which have been deleted before to start the reduction procedure, so the prereduced set consists of 35 STO. In Figure 3, we have reported the polTDDFT spectrum of SnCl_2_ employing the prereduced fit, compared with the Casida one. The match is excellent but the auxiliary set is by far too large with respect to the real necessity. In Figure 4, we have reported the 2D_*xy* descriptor trends for each molecule of the collection, together with their arithmetic mean. It is apparent that at the beginning of the procedure the reduction does not deteriorate the quality, but around *T* = 30, a sudden drop out is apparent for all the here considered systems. Quite interestingly in Figure 5, we have considered the *n*D_*y* descriptor trend on the basis set selected by the 2D_*xy* descriptor: not only the behavior is the same but also the same “best” basis set would have been identified by the *n*D_*y* descriptor. This suggests that the selection procedure is quite robust and although intrinsically descriptor‐dependent, in practice, the descriptor choice does not seem to represent a critical issue. Actually, this is not true in general: in some circumstances, the selection would be different, in that case, we checked both set (one selected by 2D_*xy* and the other one selected by *n*D_*y*) and we always found that the selection from *n*D_*y* is more accurate. In summary, the 2D_*xy* descriptor has been employed to do the reduction but the *n*D_*y* descriptor has been chosen to select the final basis. We also tried to do the reduction by *n*D_*y* descriptor, but this proven not accurate since the resulting spectra were less accurate in terms of intensity. This was not unexpected, due to the nature of the *n*D_*y* descriptor, which is not sensitive to errors due to a rescaling of the intensity. Finally, in Figure 6, we report the polTDDFT spectrum calculated for a system chosen outside the collection (Sn[CH_2_CH_3_]_2_Cl_2_), in order to verify the transferability of the so obtained auxiliary basis set. As we can see the agreement with the reference Casida results is very satisfactory confirming the transferability. In practice, this procedure has been applied to all the atoms of the periodic table, except the f‐block elements, taking into account only closed shell molecules for the collection.

It must be pointed out that, in principle, the reduction or optimization of the set for one element depends accordingly to the chosen basis of the other elements present in the molecules of the collection. However, this dependence is minimal since the other elements are previously reduced/optimized and checked for transferability. In order to avoid this dependence a method with “simultaneous” reduction or optimization should be employed but it would be computationally extremely demanding, so we accepted this dependence as a good compromise.

### 3.2 Exponent optimization of the auxiliary basis set

In most cases and for standard purposes, the basis set obtained by reduction as in the previous section does not need to be further optimized as concerns the exponents. However, there are situations in which this is necessary. In particular, at the beginning of the procedure, we do not have any basis available at all. Therefore, we started from hydrogen as the first element, which we take from a previous optimization.^1^ Then we reduced and optimized in order the following elements: C, O, Cl, S, F, N, Se, Si, P, Br, and I. In case the collection includes elements not yet available, we employed the previous optimized auxiliary basis set from.^1^

Carbon has demonstrated as one of the trickiest atoms to optimize, probably due to its very complex chemistry, which makes it to be in very different chemical contexts.

**FIGURE 7:**
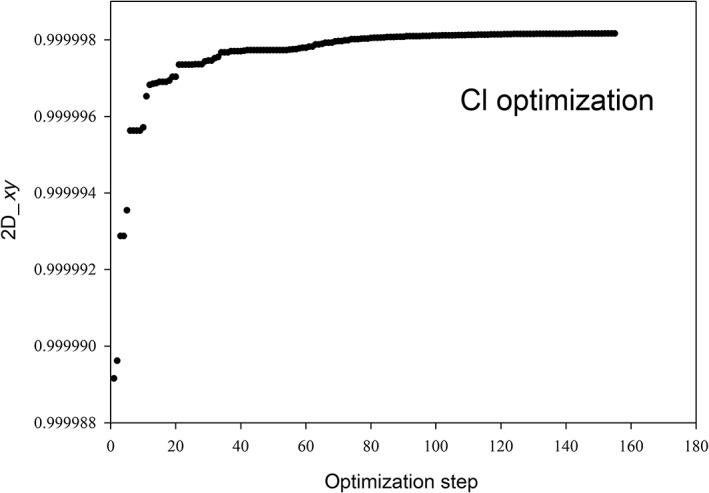
Evolution of the 2D_*xy* descriptor along the optimization procedure (exponent refinement) of the auxiliary basis set for the Cl element, calculated as arithmetic mean for HCl, CCl_4_, and Cl_2_O, which belongs to the Cl collection

**FIGURE 8:**
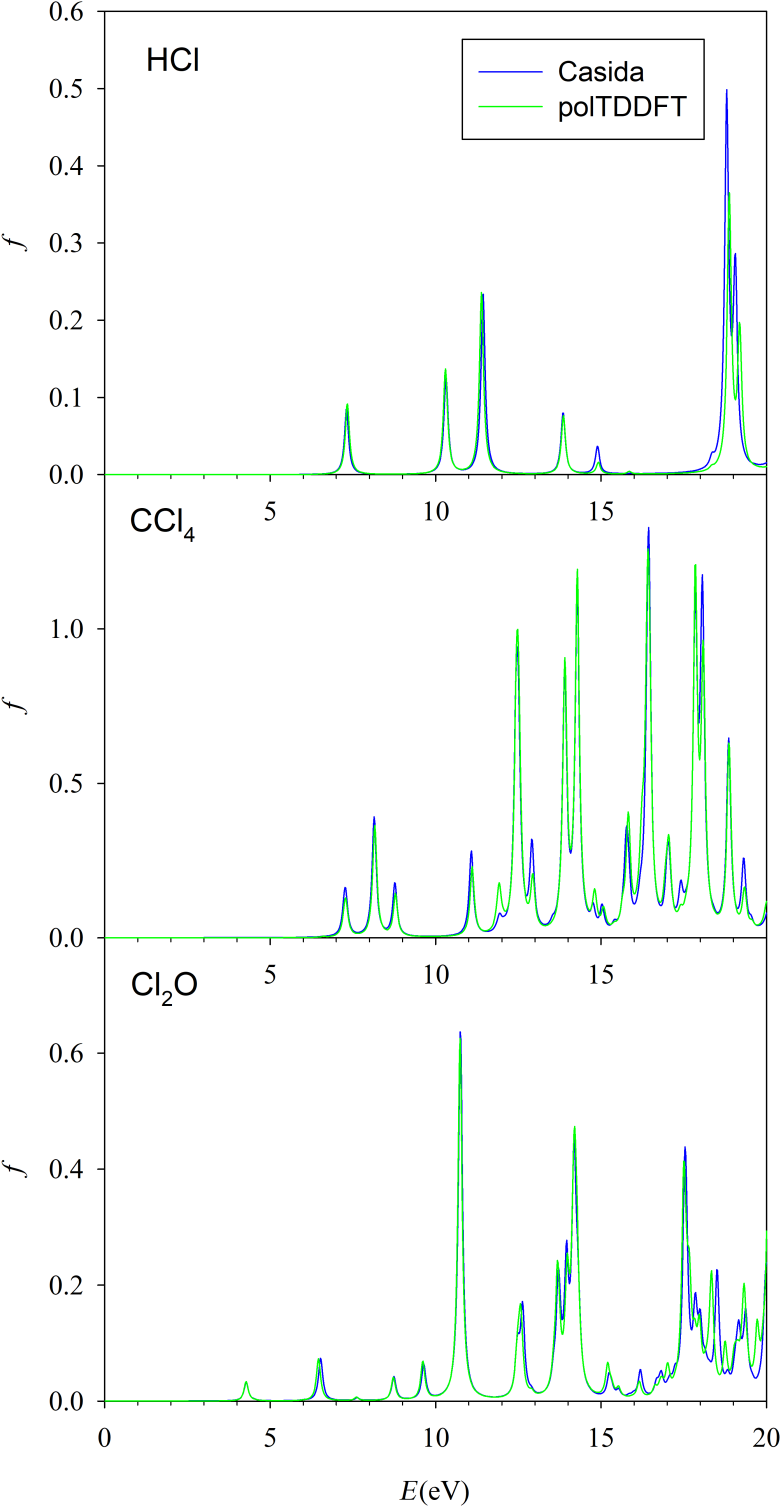
Comparison between reference (Casida) and polTDDFT spectra with optimized exponents basis set for HCl, CCl_4_, and Cl_2_O, which belong to the Cl collection

**FIGURE 9:**
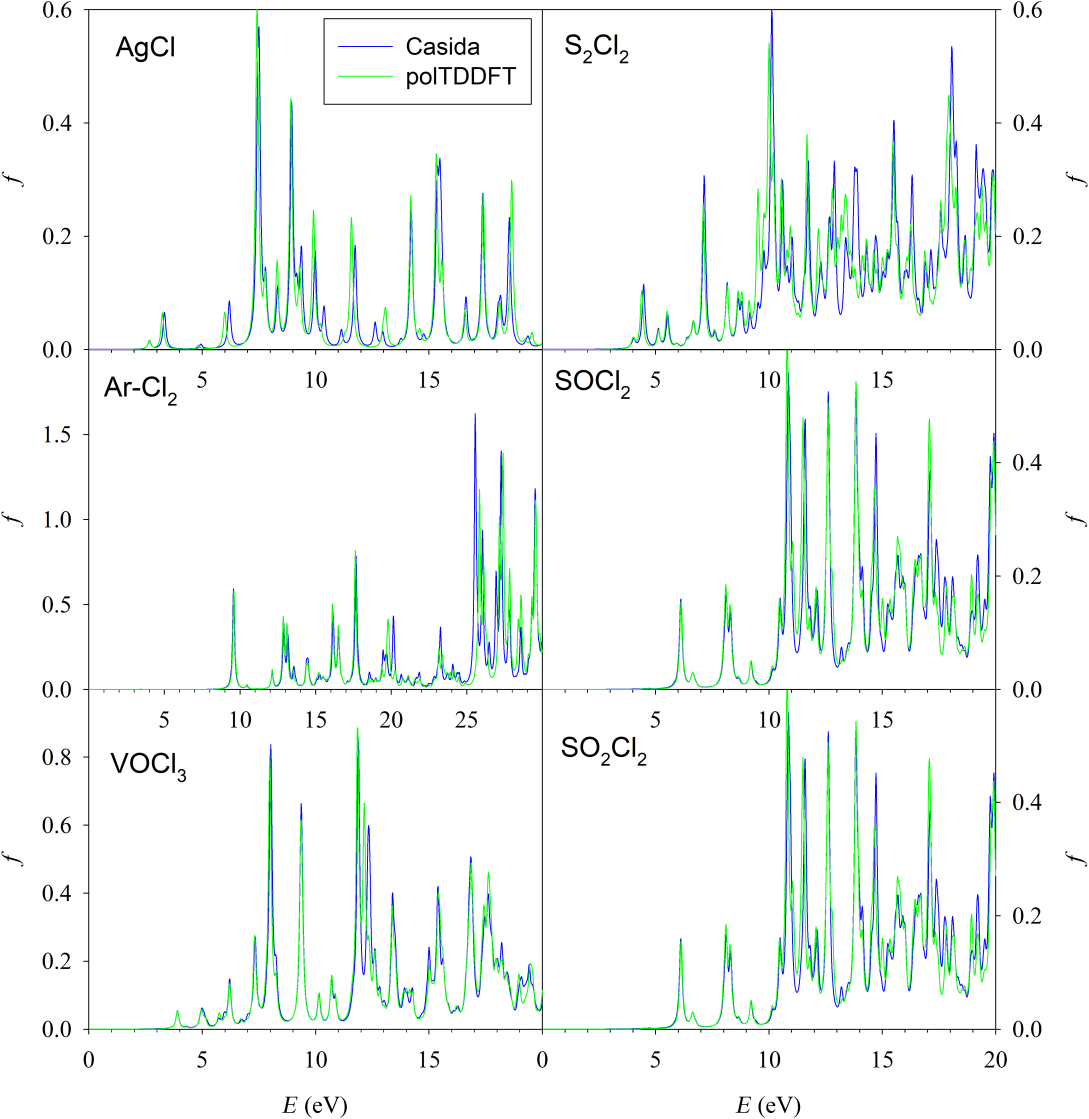
Comparison between reference (Casida) and polTDDFT spectra with cl optimized exponents basis set for AgCl, Ar‐Cl_2_, VOCl_3_, S_2_Cl_2_, SOCl_2_, and SO_2_Cl_2_, which do not belong to the Cl collection

As a typical example, we discuss the optimization results relative to the exponent refinement of the auxiliary basis set for the chlorine atom. The collection employed for optimization and previous reduction consists of three molecules: HCl, CCl_4_, and Cl_2_O. The trend of the descriptor during the process of exponent optimization is considered in figure 7, where the value of the 2D_*xy* descriptor is reported for each basis calculated accordingly to the procedure outlined in Section 2.5. It is well apparent that the optimization is quite smooth: at the beginning of the process the descriptor increases, but it assumes quickly a flat behavior indicating that the optimization is in practice completed. In Figure 8, we have reported the polTDDFT spectra of the three molecules belonging to the Cl collection, calculated with the optimized Cl basis set, in comparison with the reference Casida TDDFT spectra. The agreement is fairly nice for all molecules over the complete energy range here considered. In order to check the “transferability” of the basis, we have repeated the same analysis for six molecules not belonging to the collection, namely: S_2_Cl_2_, SOCl_2_, SO_2_Cl_2_, VOCl_3_ AgCl, and ArCl_2_, whose spectra are reported in Figure 9. ArCl_2_ is just a model system we used to reduce the basis for argon, in which Cl is in an unconventional molecular context: nevertheless, the agreement between Casida and polTDDFT is excellent in this case as well. This finding suggests that the so‐obtained auxiliary basis are transferable in different chemical context, keeping the good accuracy showed during the reduction or optimization processes.

**FIGURE 10:**
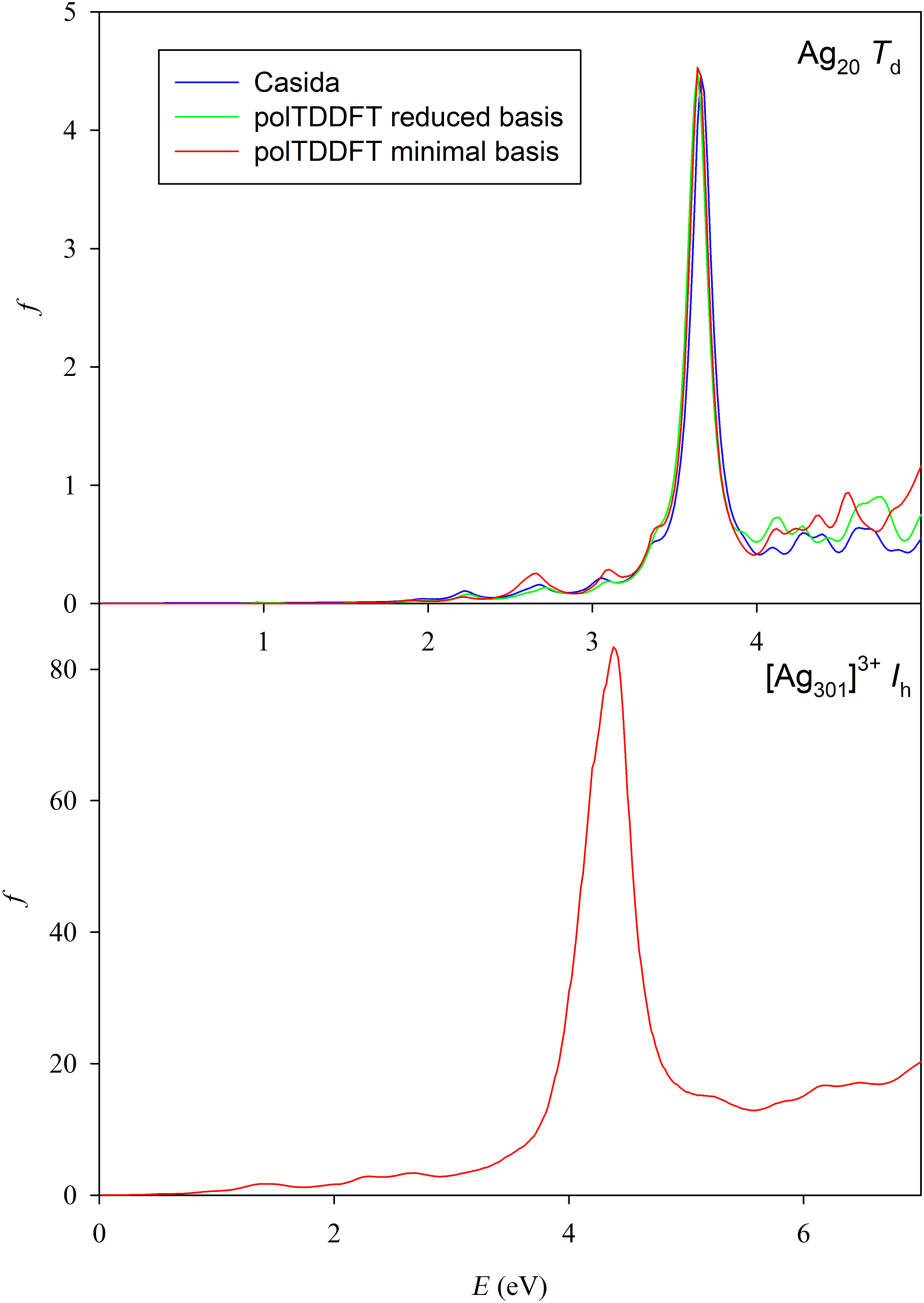
Upper panel: Comparison between reference (Casida) and polTDDFT spectra with auxiliary minimal basis with optimized exponent (*α* = 0.40) for Ag_20_. Lower panel: polTDDFT spectra calculated with auxiliary minimal basis with optimized exponent (*α* = 0.40) and DZ conventional basis for the molecular orbitals and LB94 XC potential for [Ag_301_]^3+^

As a final test, we have considered that in a recent work from Della Sala group,^15^ it has been found that for silver clusters, it is possible to obtain very accurate photoabsorption spectra by using a minimal auxiliary basis set to fit the transition density. So we have directly optimized the exponent of a single 1*s* STO function for a collection consisting of only the Ag_20_ *T*_d_ neutral cluster, obtaining a best exponent value equal to 0.40. The agreement with the reference Casida spectrum is fairly nice indeed (upper panel in Figure 10). Such basis allows to calculate huge systems, for example in the lower panel in Figure 10 we have reported the polTDDFT absorption spectrum of [Ag_301_]^3+^ calculated with the optimized auxiliary minimal basis, a conventional DZ basis for the molecular orbital and the LB94 functional,^25^ in order to be consistent with the spectrum already reported for the same systems.^3^ Thanks to the minimal size of the basis set, such calculation is extremely cheap: using the hardware described in Section 2.4. The whole calculation took only 64 h using 24 cores. We optimized a minimal basis set for gold, but the spectrum obtained was very poor: gold atom is more demanding in terms of basis size. This is due to the more important role played by the 5*d* shell, while for Ag, the 4*d* manifold is much deeper, and only 5*s* electrons are responsible for the optical properties.

### 3.3 Auxiliary basis set database organization

The database covers all the periodic table of elements, except lanthanides and actinides. The auxiliary basis are all obtained by reduction, furthermore for the C, O, Cl, S, F, N, Se, Si, P, Br, and I elements also the exponent optimization has been done. The set is already available in the last AMS2022 distributed release, in the $AMSRESOURCES/POLTDDFT directory. The complete auxiliary basis sets have been reported in Data S1.

## 4 CONCLUSIONS

In the present work, a new procedure to generate a set of STO auxiliary basis function suitable to fit the induced electron density is proposed, implemented, and applied. Such set has been optimized for each element of the periodic table (except the f‐block elements) in order to furnish accurate absorption spectra using the complex polarizability algorithm of TDDFT, also known as polTDDFT. To obtain such result we have set up an automatic procedure which is able, thanks to the definition of suitable descriptors, to evaluate the resemblance of the auxiliary basis dependent calculated spectra with respect to a reference spectrum. In particular, two different descriptors have been considered, which are very easy to calculate and have proven very efficient to quantify the resemblance of a calculated spectrum with respect to the reference spectrum. In this way, it is possible to reduce the size of the basis set maximizing the basis set accuracy. Thanks to the choice to employ a collection of molecules for each element, such basis has proven transferable to molecules outside the collection. It has been found that for most elements the reduction of the auxiliary basis set size by deleting the unnecessary functions is enough to get accurate results and small auxiliary basis sets. For some elements, a further exponent refinement has been found useful for a further improvement. The final sets are therefore much more accurate and smaller than the previous ones and have been already included in the database present in the last release of the AMS program. The availability of the present new set will allow to improve drastically the applicability range of the polTDDFT method with higher accuracy and less computational effort.

## Supporting information


**Appendix S1**Supporting Information

## Data Availability

Data are available in the Supporting Information of this article.
