# Supplementary material for: Optimization of density fitting auxiliary Slater‐type basis functions for time‐dependent density functional theory
Source: J Comput Chem. 2022 Sep 7;43(28):1923–35. doi: 10.1002/jcc.26992 (PMC9825902; doi:10.1002/jcc.26992)
Supplement: Supplementary file 1 — Appendix S1Supporting Information [file JCC-43-1923-s001.docx]

**Supporting Information for:**

**Optimization of Density Fitting Auxiliary Slater Type Basis Functions for Time Dependent Density Functional Theory**

Marco Medves^1^, Giovanna Fronzoni^1^, and Mauro Stener^1*^

^1^ Dipartimento di Scienze Chimiche, Università di Trieste, Via L. Giorgieri 1, 34127 Trieste, Italy.

**Ag.4p collection:**

Ag6 D3h

Ag -3.051087 0.000000 0.000000

Ag -0.795528 -1.377895 0.000000

Ag 1.525543 -2.642319 0.000000

Ag -0.795528 1.377895 0.000000

Ag 1.591056 0.000000 0.000000

Ag 1.525543 2.642319 0.000000

AgCl

Ag -0.001538 -0.016205 -0.086097

Cl 0.104441 -0.160457 2.175226

(PH3)Ag(SCH3)Ag(CN)

Ag -0.772440 1.191995 0.439632

S 0.669445 2.981785 1.158374

H 0.704226 4.325802 -0.843442

P -1.460133 -1.046785 0.144335

Ag 1.787091 0.889328 1.537680

H -2.535164 -1.574281 0.915693

H -0.345332 -1.893318 0.540834

H -1.832382 -1.569810 -1.127255

C 1.411347 3.623449 -0.381018

C 1.883849 -1.130236 1.512299

H 1.655924 2.825489 -1.092526

H 2.330735 4.163120 -0.114312

N 1.441727 -2.187554 1.294320

Ag(NH3)(OH)

Ag 0.346226 -1.523098 0.868646

N 1.820599 -0.280585 0.031980

H 1.424524 0.310240 -0.707473

H 2.234880 0.340940 0.735686

H 2.582206 -0.827588 -0.385443

O -0.955020 -2.734753 1.709668

H -1.835897 -2.483891 1.370058

**Al.2p collection:**

Al2H6

Al 1.291152 0.000002 0.000028

Al -1.291154 0.000001 0.000028

H -0.000001 0.000017 1.157271

H -0.000001 0.000017 -1.157230

H 1.968267 1.430359 0.000051

H 1.968220 -1.430378 0.000051

H -1.968265 1.430361 0.000051

H -1.968219 -1.430380 0.000051

Al(CH3)3

Al -0.000413 0.000551 -0.000001

C -0.002536 1.944050 -0.000002

C -1.681249 -0.974672 0.000002

C 1.683562 -0.969161 0.000000

H -1.013003 2.382574 -0.000014

H -1.553477 -2.068677 -0.000009

H 2.568471 -0.313205 0.000005

H 0.535892 2.336939 0.880714

H 0.535912 2.336934 -0.880709

H -2.291237 -0.705938 0.880704

H -2.291250 -0.705921 -0.880685

H 1.754661 -1.631840 0.880771

H 1.754667 -1.631833 -0.880777

Al(OH)3

Al -0.000131 0.000586 0.000000

O -0.006166 1.687191 0.000000

O -1.457356 -0.848761 0.000000

O 1.463084 -0.838448 0.000000

H -0.841750 2.182996 0.000000

H -1.468090 -1.820457 0.000000

H 2.310508 -0.363107 0.000000

AlCl3

Al 0.000000 0.000000 0.000000

Cl 0.000000 2.060000 0.000000

Cl 1.784000 -1.030000 0.000000

Cl -1.784000 -1.030000 0.000000

**Ar.2p collection:**

Ar2

Ar -0.719834 0.000000 0.000000

Ar 2.719834 0.000000 0.000000

ArCl2

Ar 0.000000 0.000000 0.926244

Cl 0.000000 -1.002922 -2.463122

Cl 0.000000 1.002922 -2.463122

Ar(C6H6)

Ar -0.000010 0.000069 3.164340

C 0.693457 1.201102 -0.138841

C 1.386801 -0.000005 -0.138866

C -0.693455 1.201096 -0.138838

C 0.693463 -1.201116 -0.138872

C -1.386799 -0.000004 -0.138865

C -0.693457 -1.201110 -0.138872

H 2.481434 -0.000003 -0.138542

H 1.241019 2.148882 -0.138511

H -1.241023 2.148872 -0.138509

H 1.241027 -2.148895 -0.138540

H -1.241025 -2.148886 -0.138542

H -2.481430 -0.000003 -0.138542

Ar

Ar 0.000000 0.000000 0.000000

**As.3d collection:**

AsH3

As 0.000000 0.000847 0.027978

H 0.000000 1.299709 -0.848953

H 1.124404 -0.650278 -0.849512

H -1.124404 -0.650278 -0.849512

As2O3

O -2.748816 0.471853 0.000000

As -1.710814 1.796660 0.000000

O 0.000000 0.967973 0.000000

As 1.710814 1.796660 0.000000

O 2.748816 0.471853 0.000000

AsCl3

As 0.000000 0.000064 0.013926

Cl 0.000000 2.016797 -1.051322

Cl 1.747206 -1.008480 -1.051352

Cl -1.747206 -1.008480 -1.051352

**At.5d collection:**

At2O

O -4.981457 0.369886 0.000000

At -3.100285 1.409794 0.000000

At -4.941558 -1.779780 0.000000

AtH

At 0.121536 0.000000 0.000000

H 1.861064 0.000000 0.000000

AtI

At -0.057543 0.000000 0.000000

I 2.837543 0.000000 0.000000

**Au.4f collection:**

Au6 D3h

Au -2.987853 0.000000 0.000000

Au -0.789972 -1.368271 0.000000

Au 1.493926 -2.587557 0.000000

Au -0.789972 1.368271 0.000000

Au 1.579943 0.000000 0.000000

Au 1.493926 2.587557 0.000000

AuCl

Au 0.000043 -0.018357 -0.052360

Cl 0.102860 -0.158305 2.141489

(PH3)Au(SCH3)Au(CN)

Au -0.708470 0.888698 0.490954

S 0.638964 2.646495 1.189683

H 0.516963 3.998689 -0.800451

P -1.997605 -0.845333 -0.100230

Au 1.967724 0.771593 1.578284

H -3.282239 -0.986804 0.498201

H -1.482824 -2.154513 0.124651

H -2.365842 -0.967112 -1.470119

C 1.294398 3.365008 -0.353296

C 3.001846 -0.848000 1.897871

H 1.609355 2.588449 -1.059831

H 2.160787 3.982222 -0.078260

N 3.585837 -1.840407 2.067156

**Ba.4d collection:**

Ba(OH)2

Ba -0.000003 -0.000001 0.000000

O 2.265422 0.385078 0.004160

H 3.224837 0.548160 0.005922

O -2.265419 -0.385078 -0.004160

H -3.224836 -0.548160 -0.005922

BaCO3

Ba 4.289972 -0.253440 0.318072

O 6.130635 1.089398 0.144076

C 6.993005 0.071893 -0.067307

O 8.186980 0.217350 -0.227650

O 6.368309 -1.124800 -0.075691

BaSO4

Ba 2.826892 0.544015 -2.396213

O 3.237286 -0.832584 -4.416323

S 4.672559 -0.346941 -4.196346

O 4.498072 1.171882 -4.114450

O 4.932921 -0.729719 -2.738365

O 5.640171 -0.811554 -5.149002

**B.1s collection:**

B2H6

B 0.880000 0.000000 0.000000

B -0.880000 0.000000 0.000000

H 0.000000 0.000000 0.980000

H 0.000000 0.000000 -0.980000

H 1.461800 1.049500 0.000000

H 1.461800 -1.049500 0.000000

H -1.461800 1.049500 0.000000

H -1.461800 -1.049500 0.000000

BF3

B 0.000000 0.000000 0.000000

F 0.000000 1.307000 0.000000

F 1.131900 -0.653500 0.000000

F -1.131900 -0.653500 0.000000

H3BO3

O -1.604801 0.052962 0.083931

B -0.241383 0.022439 0.126794

O 0.403312 0.016137 1.329411

O 0.477623 0.002173 -1.032880

H -1.990669 0.065484 0.979695

H 1.371799 -0.004885 1.214695

H -0.105349 0.011505 -1.814801

**Be.1s collection:**

BeH2

Be 0.000000 0.000000 0.000000

H 0.000000 0.000000 1.326400

H 0.000000 0.000000 -1.326400

BeCl2

Be 0.000000 0.000000 0.000000

Cl 0.000000 0.000000 1.750000

Cl 0.000000 0.000000 -1.750000

BeO

Be 0.000000 0.000000 0.000000

O 0.000000 0.000000 1.330900

**Bi.5d collection:**

Bi2O3

O 1.098086 1.668394 -2.682431

Bi 1.203704 0.055010 -1.561076

O -0.269461 0.564686 -0.087612

Bi -0.994561 -0.494448 1.631687

O -2.276468 0.837357 2.305431

BiOCl

Cl -2.083985 0.529622 0.000000

Bi 0.321469 -0.257239 0.000000

O 1.337017 1.425417 0.000000

BiH3

Bi 0.026093 -0.155255 0.148058

H 0.173678 -0.368080 -1.675628

H 1.018252 1.391422 0.022641

H -1.505023 0.833812 -0.116270

BiF5

F -1.337921 -0.424782 -1.487367

Bi -0.000994 -0.003347 0.001273

F 1.331223 0.420223 1.493640

F -1.529857 0.634447 1.188432

F 0.593631 -1.953084 0.022222

F 0.944518 1.326243 -1.219500

**Br.3d collection:**

HBr

H 0.000000 0.000000 0.000000

Br 0.000000 0.000000 1.414400

CBr4

C 0.000000 0.000000 0.000000

Br 1.121200 1.121200 1.121200

Br -1.121200 -1.121200 1.121200

Br -1.121200 1.121200 -1.121200

Br 1.121200 -1.121200 -1.121200

HBrO

H -0.917600 1.658900 0.000000

O 0.021300 1.454200 0.000000

Br 0.021300 -0.379800 0.000000

**C.1s collection:**

CH4

C 0.000000 0.000000 0.000000

H 0.000000 0.000000 1.089000

H 1.026719 0.000000 -0.363000

H -0.513360 -0.889165 -0.363000

H -0.513360 0.889165 -0.363000

CO

C 0.000000 0.000000 0.000000

O 0.000000 0.000000 1.128000

C2H4

C 0.000000 0.000000 0.669500

C 0.000000 0.000000 -0.669500

H 0.000000 0.928900 1.232100

H 0.000000 -0.928900 1.232100

H 0.000000 0.928900 -1.232100

H 0.000000 -0.928900 -1.232100

C2H2

C 1.016900 0.000000 0.000000

H 0.000000 0.000000 0.000000

C 1.983100 0.000000 0.000000

H 3.000000 0.000000 0.000000

C6H4O2

H 0.000000 2.182973 1.259286

H 0.000000 -2.182973 1.259286

H 0.000000 2.182973 -1.259286

H 0.000000 -2.182973 -1.259286

C 0.000000 0.000000 1.441079

C 0.000000 0.000000 -1.441079

C 0.000000 1.266644 0.674582

C 0.000000 -1.266644 0.674582

C 0.000000 1.266644 -0.674582

C 0.000000 -1.266644 -0.674582

O 0.000000 0.000000 2.678518

O 0.000000 0.000000 -2.678518

**Ca.3p collection:**

CaH2

Ca -3.630601 0.133050 -2.746160

H -5.534008 -0.416655 -3.148454

H -2.923091 1.414005 -1.344886

CaC2

Ca -3.951197 0.253303 -3.214845

C -2.546260 -1.130332 -2.254653

C -2.725543 -0.178871 -1.447102

CaO

Ca 0.000000 0.000000 0.000000

O 0.000000 0.000000 1.822100

**Cd.4p collection:**

CdS

Cd 0.000000 0.000000 -0.128982

S 0.000000 0.000000 2.128982

Cd(CH3)2

Cd 2.643858 -1.049154 -0.925326

C 4.479223 -1.053845 0.132568

C 0.809920 -1.053866 -1.985738

H 4.289137 -1.052311 1.215948

H 5.068802 -0.162014 -0.126239

H 5.061625 -1.950804 -0.124919

H -0.033875 -1.057942 -1.279964

H 0.744288 -1.948004 -2.623127

H 0.737008 -0.159105 -2.621422

CdO

Cd 2.556427 -0.934337 -0.323830

O 2.476773 -0.206005 -2.072914

CdCl2

Cd 3.186571 -0.780103 -0.696753

Cl 4.406882 -1.522066 1.085178

Cl 1.966258 -0.038138 -2.478689

**Cl.2p collection:**

CCl4

C 0.000000 0.000000 0.000000

Cl 1.020200 1.020200 1.020200

Cl -1.020200 -1.020200 1.020200

Cl -1.020200 1.020200 -1.020200

Cl 1.020200 -1.020200 -1.020200

HCl

Cl 0.000000 0.000000 0.000000

H 0.000000 0.000000 1.274600

Cl2O

O 0.000000 0.000000 0.778800

Cl 0.000000 1.396600 -0.183200

Cl 0.000000 -1.396600 -0.183200

**Co.3p collection:**

CoF3

Co 1.921094 1.691281 -0.336827

F 3.510202 1.303377 0.051004

F 1.533275 1.303354 -1.925934

F 1.533237 3.280380 0.051065

Co(NH3)3(Cl)3

N -1.260275 0.745111 -1.400381

H -0.300760 0.427154 -1.633262

N -1.528586 -1.470718 0.436222

H -1.272183 1.780680 -1.460000

N -3.210042 0.862714 0.729316

H -1.955111 0.326501 -2.028430

H -3.364357 0.625503 1.727475

Co -1.312146 0.465052 0.528887

Cl -1.349085 0.160382 2.738298

Cl 0.865633 0.027722 0.319878

Cl -1.043280 2.675680 0.653832

H -3.921790 0.431787 0.129172

H -3.220921 1.896429 0.646268

H -0.564400 -1.774107 0.202205

H -2.219373 -1.914536 -0.178886

H -1.700392 -1.710254 1.430755

Co(OH2)3(CN)3

Co 1.823499 1.604248 -0.340973

C 3.614947 1.577833 -0.155395

C 1.853619 1.594993 -2.141387

C 1.683194 3.394109 -0.194519

N 1.388088 4.488803 0.083263

N 4.717907 1.359206 0.158070

O 1.907461 -0.411990 -0.443596

O -0.191294 1.564226 -0.489007

O 1.728454 1.532969 1.674695

N 1.666398 1.363869 -3.270253

H -0.424153 2.511488 -0.326067

H 1.538582 2.475503 1.905578

H 2.682916 1.423836 1.910900

H 1.826923 -0.564313 -1.417903

H 2.864430 -0.581260 -0.260432

H -0.311256 1.460407 -1.465606

**Cr.3p collection:**

H2CrO4

O -1.737047 -3.241957 -0.621146

Cr -0.586888 -1.913228 -0.528002

O 0.888171 -2.483822 -0.485678

O -0.838807 -0.970297 0.934616

H -1.369676 -4.066509 -0.231251

O -0.816937 -1.000815 -1.799806

H -1.448016 -0.216272 0.771167

CrO2Cl2

Cr 1.599376 0.293266 -0.544981

O 2.878918 -0.462610 -0.020866

Cl 1.652813 0.370878 -2.654154

O 0.318963 -0.504873 -0.089801

Cl 1.541525 2.229012 0.296489

CrO3

Cr 1.578206 0.274510 -0.840605

O 2.935828 -0.182307 -0.133153

O 1.565850 -0.204085 -2.364500

O 0.321969 -0.332400 -0.062955

Cr(CO)6

Cr 2.189438 -2.449693 -2.975168

C 0.495467 -1.750593 -2.526350

O -0.534672 -1.324521 -2.256528

C 2.184558 -3.475873 -1.391769

C 3.020086 -1.028387 -2.053782

C 1.358842 -3.871249 -3.896227

C 2.194170 -1.423814 -4.558765

O 2.180920 -4.100945 -0.430298

C 3.883628 -3.148260 -3.423949

O 3.523076 -0.165481 -1.489811

O 2.197582 -0.799030 -5.520443

O 0.855953 -4.734518 -4.459717

O 4.914086 -3.573705 -3.693583

**Cs.4d collection:**

CsOH

Cs -0.000006 0.000016 2.332388

O 0.000001 -0.000004 -0.029644

H 0.000004 -0.000012 -1.003244

CsCH3

Cs -2.618056 0.000081 -0.004356

C 0.222490 -0.000056 0.001098

H 0.635897 -0.000013 1.028511

H 0.634716 0.890087 -0.512581

H 0.634953 -0.890099 -0.512571

CsF

Cs -0.260950 0.000000 0.000000

F 2.060950 0.000000 0.000000

Cs2

Cs 4.066790 0.000000 0.000000

Cs -0.466790 0.000000 0.000000

**Cu.3p collection:**

Cu2O

Cu -0.391430 -0.049686 0.045684

O -1.953137 -0.714863 0.463508

Cu -2.715063 0.180036 -0.830914

Cu2S

Cu -0.616343 0.248257 -0.094200

S -2.197723 -0.925769 0.561495

Cu -2.977992 0.258824 -0.954146

Cu(NH3)3(CN)

Cu 0.192820 -0.860547 -0.471955

C 0.146036 -0.500244 -2.297340

N 0.122753 -0.283749 -3.446073

H -0.470044 -3.381939 -0.501404

N 1.793711 0.012971 0.627249

N -1.557015 -0.270195 0.609515

H 2.030236 -0.188010 1.605517

H -1.733258 -0.398963 1.612278

N 0.376298 -2.972181 -0.092945

H 0.554308 -3.447523 0.799068

H 1.666789 1.026838 0.541384

H -1.645952 0.730155 0.402841

H 2.613963 -0.215367 0.055683

H -2.318623 -0.727881 0.097909

H 1.138364 -3.213054 -0.735509

**F.1s collection:**

HF

F 0.000000 0.000000 0.000000

H 0.000000 0.000000 0.916800

CF4

C 0.000000 0.000000 0.000000

F 0.759300 0.759300 0.759300

F -0.759300 -0.759300 0.759300

F -0.759300 0.759300 -0.759300

F 0.759300 -0.759300 -0.759300

OF2

O 0.000000 0.000000 0.604900

F 0.000000 1.100300 -0.268800

F 0.000000 -1.100300 -0.268800

**Fe.3p collection:**

Fe(CO)5

Fe 6.240049 -1.915834 2.258329

C 6.380116 -2.967612 3.701664

C 6.015952 -0.488427 3.316270

O 6.466569 -3.642853 4.623723

O 5.867274 0.425092 3.996008

C 4.816556 -2.853424 1.714758

C 6.101389 -0.862777 0.815729

C 7.882249 -2.408932 1.742722

O 3.903160 -3.454979 1.364581

O 8.933825 -2.730835 1.411927

O 6.017043 -0.185587 -0.104969

Fe(H2S)4(CN)2

Fe 0.122694 -0.369162 0.006064

C -0.227825 -0.121132 1.855564

C 0.474308 -0.617602 -1.841589

N 0.715070 -0.758842 -2.977702

N -0.465493 0.019687 2.992354

S -0.426907 -2.494125 0.195357

S 0.670754 1.758189 -0.177046

S 2.222175 -0.873194 0.457600

H -1.491519 -2.565555 1.058138

H 1.699220 1.833904 -1.082259

H 2.225227 -1.698320 1.553578

S -1.977083 0.136696 -0.440752

H -2.326443 1.183400 0.374205

H -1.985561 0.939005 -1.553314

H -1.215658 -2.820849 -0.878473

H 1.505305 2.068067 0.866866

H 2.583746 -1.900994 -0.375955

Fe(NH3)4(CN)2

Fe 1.924586 -0.887667 1.735917

H 2.360772 -3.491197 1.156054

N 2.127125 -2.632599 0.640779

H 2.952174 -2.387181 0.061056

H 1.367301 -2.884136 -0.004830

N 3.192814 -1.545327 3.130246

N 0.292229 -1.582011 2.811033

N 0.739693 -0.070736 0.355561

H 0.116838 -0.759867 3.420492

H 0.437095 -2.391793 3.427980

H -0.576656 -1.768139 2.293607

H 4.137936 -1.417101 2.740074

H 3.129106 -2.506566 3.496192

H 3.112767 -0.896323 3.926827

H -0.028142 -0.612378 -0.067305

H 1.357941 0.248254 -0.404213

H 0.334530 0.775786 0.781053

C 1.668988 0.603676 2.760386

N 1.271228 1.387844 3.543928

C 3.369603 -0.363107 0.749156

N 4.281230 -0.302444 0.006596

Fe(NH3)4(NO2)2

Fe 0.409669 -0.278628 -0.334124

N -0.184568 1.549794 0.117396

N -1.471986 -0.877250 -0.306480

H -1.690585 -0.687813 -1.329625

N 1.030104 -2.137678 -0.567102

N 0.181841 0.000483 -2.183596

N 2.262351 0.356848 -0.580750

H -1.139464 1.788496 -0.180058

H 2.997224 -0.311251 -0.314729

H 1.216847 -2.349688 0.457970

H 0.344061 -2.798522 -0.953823

H 2.501019 1.242984 -0.117201

N 0.629915 -0.550012 1.517261

H 0.415966 2.314313 -0.217277

H -1.631141 -1.867535 -0.080696

O 1.087608 -1.632775 1.971853

H -0.121614 1.459529 1.174942

H 2.225365 0.485586 -1.635357

H 1.883219 -2.249054 -1.130061

H -2.095054 -0.339509 0.309986

O 0.321347 0.343586 2.351157

O -0.940221 -0.156026 -2.736159

O 1.142956 0.358630 -2.915864

Fe(OH2)4(Cl)2

Fe 0.183718 0.156253 -0.138838

O 0.079849 2.117471 0.029192

O -1.786670 0.054879 0.022358

H -2.098486 0.900982 0.411238

O 0.289951 -1.805110 -0.302952

Cl -0.325479 -0.005793 -2.348723

O 2.151154 0.254032 -0.305764

H 0.446181 2.109998 0.966817

H 2.273114 0.161550 0.690051

H -0.078575 -1.803679 -1.239668

H -0.403907 -2.180777 0.280722

H 2.457218 -0.592808 -0.697278

Cl 0.696422 0.312749 2.069175

H 0.776896 2.493130 -0.550714

H -1.903697 0.144700 -0.974727

**Fr.5d collection:**

Fr2

Fr 4.030481 0.000000 0.000000

Fr -0.430481 0.000000 0.000000

FrF

Fr -0.294912 0.000000 0.000000

F 2.094912 0.000000 0.000000

FrOH

Fr 0.000003 0.000032 2.373993

O -0.000001 -0.000008 -0.050730

H -0.000003 -0.000024 -1.023763

**Ga.3d collection:**

GaCl3

Ga 0.000000 0.013788 0.000000

Cl 0.000000 2.281367 0.000000

Cl 1.966630 -1.111428 0.000000

Cl -1.966630 -1.111428 0.000000

Ga2O3

O -3.337156 1.709111 0.000000

Ga -1.711252 0.980833 0.000000

O 0.000000 0.125111 0.000000

Ga 1.711252 0.980833 0.000000

O 3.337156 1.709111 0.000000

GaH3

Ga 0.000000 0.004070 0.000000

H -1.410684 0.821143 0.000000

H 1.410684 0.821143 0.000000

H 0.000000 -1.626356 0.000000

**Ge.3d collection:**

Ge(CH3)4

Ge -0.001211 0.007948 -0.001165

C 1.727704 0.999560 -0.005120

C -1.517807 1.300039 -0.005099

C -0.103020 -1.139598 -1.628476

C -0.106756 -1.147665 1.627436

H 2.566953 0.289124 -0.005217

H 1.801250 1.634411 0.889433

H 1.801429 1.635391 -0.898813

H -2.473033 0.755688 -0.004978

H -1.472995 1.936936 0.889720

H -1.473572 1.938589 -0.898602

H -1.046750 -1.703639 -1.633452

H 0.734549 -1.851916 -1.637579

H -0.053584 -0.523094 -2.537285

H 0.804463 -1.037299 2.232044

H -0.973779 -0.863580 2.240215

H -0.211140 -2.202396 1.336237

GeO2

Ge 0.504121 -0.046924 0.506315

O 1.446034 1.059098 -0.335201

O -0.438255 -1.153374 1.346785

GeCl4

Ge 0.000000 0.000000 0.000000

Cl 0.000000 -1.779382 -1.258098

Cl 0.000000 1.779382 -1.258098

Cl -1.779382 0.000000 1.258098

Cl 1.779382 0.000000 1.258098

GeH4

Ge -0.001041 0.001562 -0.001815

H 1.344302 0.776994 -0.001639

H -1.180585 1.010550 -0.001689

H -0.080986 -0.890952 -1.269789

H -0.081089 -0.891554 1.265631

**He collection:**

He

He 0.000000 0.000000 0.000000

He2

He -0.202435 0.000000 0.000000

He 2.202435 0.000000 0.000000

He(C6H6)

He -0.000090 0.000131 2.532908

C 0.693352 1.200805 -0.086710

C 1.386687 -0.000008 -0.087019

C -0.693336 1.200796 -0.086691

C 0.693357 -1.200830 -0.086740

C -1.386674 -0.000009 -0.086987

C -0.693337 -1.200821 -0.086720

H 2.481223 -0.000007 -0.085584

H 1.240799 2.148563 -0.085215

H -1.240788 2.148550 -0.085193

H 1.240806 -2.148585 -0.085269

H -1.240791 -2.148574 -0.085245

H -2.481209 -0.000010 -0.085536

HeCl2

He -1.272385 -0.000032 0.000000

Cl 1.636138 -1.001781 0.000000

Cl 1.636247 1.001814 0.000000

**Hf.4f collection:**

HfCl4

Cl -2.124407 0.177085 -0.808828

Hf 0.001689 0.000328 -0.000410

Cl 0.579368 1.939587 1.054449

Cl 0.105862 -1.727809 1.485713

Cl 1.437487 -0.389291 -1.730924

HfO2

Hf 1.350262 1.078053 0.242627

O -0.020968 -0.026317 0.102697

O 2.724661 0.175314 -0.401925

HfH4

Hf 5.707521 -2.139598 3.437492

H 7.480772 -2.139302 3.872770

H 5.528769 -2.139303 1.620167

H 4.912359 -0.648886 4.127423

H 4.911882 -3.630066 4.127514

**Hg.4f collection:**

Hg(NH2)(Cl)

Cl -1.701058 0.225842 1.122554

Hg 0.202258 -0.026911 -0.089836

N 1.793603 -0.237669 -1.282129

H 2.471275 0.493262 -1.025595

H 2.247822 -1.128124 -1.037294

Hg(CH3)2 C3v

C 0.000000 0.000000 -2.080230

Hg 0.000000 0.000000 0.000056

H -0.516138 0.893977 -2.463169

H -0.516138 -0.893977 -2.463169

H 1.032276 0.000000 -2.463169

C 0.000000 0.000000 2.080408

H 0.516156 -0.894008 2.463091

H -1.032312 0.000000 2.463091

H 0.516156 0.894008 2.463091

Hg3F4 C3v

Hg -1.564344 0.000000 -0.186230

Hg 0.782172 1.354762 -0.186230

F 1.763159 3.053880 -0.232464

F 1.763159 -3.053880 -0.232464

F -3.526317 0.000000 -0.232464

F 0.000000 0.000000 1.668142

Hg 0.782172 -1.354762 -0.186230

HgO

Hg 0.000000 0.000000 -0.218309

O 0.000000 0.000000 1.661609

HgS

S 0.000000 0.000000 -1.874566

Hg 0.000000 0.000000 0.358066

Hg3I2 D2h

Hg 0.000000 0.000000 2.630400

I 0.000000 0.000000 -5.296185

Hg 0.000000 0.000000 -2.630400

I 0.000000 0.000000 5.296185

Hg 0.000000 0.000000 0.000000

Hg2Cl2 D2h

Hg 0.000000 0.000000 -1.278184

Cl 0.000000 0.000000 3.578361

Hg 0.000000 0.000000 1.278184

Cl 0.000000 0.000000 -3.578361

Hg(PH3)(NH3)(Cl)(Br)

N -1.059359 -0.314670 -0.753306

P 1.072317 1.952923 2.083428

Hg -1.397177 0.999826 1.286389

H -1.571118 -1.201318 -0.724306

Br -1.178922 -0.573379 3.205968

H 2.093505 2.920360 1.787954

H -0.109789 -0.495801 -1.090513

H -1.532975 0.326923 -1.402160

Cl -2.462357 2.588951 -0.095091

H 0.861721 2.365458 3.434342

H 1.957079 0.879821 2.413037

**I.4d collection:**

HI

I 0.000000 0.000000 0.000000

H 0.000000 0.000000 1.609200

CI4

C 0.000000 0.000000 0.000000

I 1.245300 1.245300 1.245300

I 1.245300 -1.245300 -1.245300

I -1.245300 1.245300 -1.245300

I -1.245300 -1.245300 1.245300

I2O

I -1.629500 0.000047 -0.735419

I 1.237175 0.000047 1.290540

O 0.419735 -0.000072 -0.593905

**In.4d collection:**

InCl3

In 0.000000 -0.000622 -0.007043

Cl -2.118539 1.224251 0.005647

Cl 2.118539 1.224251 0.005647

Cl 0.000000 -2.447880 0.005650

In2O3

O 0.000000 0.000000 -0.893759

In 1.931793 0.000000 -0.080540

In -1.931793 0.000000 -0.080540

O -3.797239 0.000000 0.583020

O 3.797239 0.000000 0.583020

InH3

In 0.000000 0.000794 -0.002325

H -1.578484 0.910441 0.003176

H 1.578484 0.910441 0.003176

H 0.000000 -1.821676 0.003174

**Ir.4f collection:**

Ir2(CO)8 D4h

C -1.903566 0.000000 -1.601771

C 0.000000 -1.903566 -1.601771

O -3.048099 0.000000 -1.707845

O 0.000000 -3.048099 -1.707845

Ir 0.000000 0.000000 -1.405854

C 0.000000 1.903566 -1.601771

O 0.000000 3.048099 -1.707845

C 1.903566 0.000000 -1.601771

O 3.048099 0.000000 -1.707845

C -1.903566 0.000000 1.601771

C 0.000000 -1.903566 1.601771

O -3.048099 0.000000 1.707845

O 0.000000 -3.048099 1.707845

Ir 0.000000 0.000000 1.405854

C 0.000000 1.903566 1.601771

O 0.000000 3.048099 1.707845

C 1.903566 0.000000 1.601771

O 3.048099 0.000000 1.707845

Ir(ONO)(NO2)(NH2)(NH3)2

Ir -0.379613 -0.164055 0.132587

N 0.010988 -1.400504 -1.447250

H -0.719345 -1.519003 -2.160227

H 0.187054 -2.283025 -0.920984

H 0.907810 -1.069178 -1.852609

N -0.623597 0.909104 1.872765

H -0.496466 0.235727 2.645050

H -1.540754 1.364586 1.974061

H 0.117598 1.621662 1.946952

O 1.556191 0.300717 0.327681

N 2.282672 0.068932 -0.781995

O 3.431658 0.396472 -0.660692

N -1.600605 0.996895 -0.861012

O -2.449483 0.085891 -0.410029

O -1.952080 1.948785 -1.506031

N 0.243579 -1.776909 1.165693

H 1.147292 -1.645853 1.638865

H -0.428200 -2.188544 1.821173

Ir(C2H4)2(CO)(Cl) C2v

Ir 0.000000 0.000000 -0.085471

Cl 0.000000 0.000000 2.224428

C 0.689656 2.097832 -0.002146

C -0.689656 2.097832 -0.002146

H 1.232974 2.144190 0.948004

H 1.255554 2.323894 -0.910807

H -1.232974 2.144190 0.948004

H -1.255554 2.323894 -0.910807

C 0.689656 -2.097832 -0.002146

C -0.689656 -2.097832 -0.002146

H 1.232974 -2.144190 0.948004

H 1.255554 -2.323894 -0.910807

H -1.232974 -2.144190 0.948004

H -1.255554 -2.323894 -0.910807

C 0.000000 0.000000 -1.903578

O 0.000000 0.000000 -3.065581

Ir(OH)3

Ir 0.874807 -0.801794 -0.693038

H -0.263242 -1.832334 1.045327

H 0.134450 -0.750819 -2.882262

H 2.256297 0.802329 0.254206

O -0.166836 -0.226433 -2.097047

O 2.472786 0.038113 -0.337614

O 0.314637 -2.218063 0.339326

Ir(SH)3

Ir 0.886951 -0.825643 -0.727925

H -0.441102 -1.900578 1.414988

H 0.071013 -0.827010 -3.350778

H 2.414350 1.080000 0.521785

S -0.384985 -0.076039 -2.286313

S 2.791642 0.092903 -0.367382

S 0.285030 -2.532633 0.424523

Ir(PH3)2(Cl)(CO)

Ir -0.025167 0.000088 -0.203268

Cl -0.166246 0.000760 2.132930

H 1.042256 -2.810008 0.773026

C 0.074120 0.000076 -2.021867

H -1.123272 -2.839332 0.675987

H 0.047443 -3.118397 -1.136506

P -0.015318 -2.253014 -0.004054

P -0.005343 2.252943 -0.004095

O 0.133908 0.000313 -3.182316

H 0.075787 3.117320 -1.136231

H -1.118436 2.846711 0.661124

H 1.045447 2.804469 0.785872

**K.3p collection:**

K2

K 0.000000 0.000000 0.000000

K 0.000000 0.000000 3.905100

KCH3

K -2.438065 -0.000001 -0.003796

C 0.199801 0.000001 0.000305

H 0.582982 0.000000 1.035987

H 0.582643 0.897353 -0.516198

H 0.582639 -0.897353 -0.516198

KH

K 0.000000 0.000000 0.000000

H 0.000000 0.000000 2.242500

KOH

K 0.000000 0.000000 2.211500

O 0.000000 0.000000 0.000000

H 0.000000 0.000000 -0.912000

**Kr.3d collection:**

Kr2

Kr -0.849797 0.000000 0.000000

Kr 2.849797 0.000000 0.000000

Kr(C6H6)

Kr -0.000025 0.000014 3.281060

C 0.693218 1.200685 -0.149813

C 1.386328 0.000001 -0.149863

C -0.693212 1.200678 -0.149810

C 0.693223 -1.200689 -0.149818

C -1.386324 -0.000001 -0.149861

C -0.693216 -1.200683 -0.149815

H 2.480950 0.000003 -0.147061

H 1.240764 2.148457 -0.146991

H -1.240763 2.148446 -0.146987

H 1.240770 -2.148460 -0.146995

H -1.240767 -2.148452 -0.146992

H -2.480945 0.000000 -0.147056

KrCl2

Kr -1.006868 -0.000066 0.000000

Cl 2.503375 -1.002625 0.000000

Cl 2.503494 1.002691 0.000000

Kr

Kr 0.000000 0.000000 0.000000

**Li.1s collection:**

LiH

Li 0.000000 0.000000 0.000000

H 0.000000 0.000000 1.594900

Li2O

O 0.000000 0.000000 0.000000

Li 0.000000 0.000000 1.606000

Li 0.000000 0.000000 -1.606000

Li2

Li 0.000000 0.000000 0.000000

Li 0.000000 0.000000 2.673000

LiCl

Li 0.000000 0.000000 0.000000

Cl 0.000000 0.000000 2.020700

**Mg.2p collection:**

MgC2

Mg -3.852835 0.168113 -3.133562

C -2.551406 -1.143048 -2.279519

C -2.733759 -0.188465 -1.473919

MgH2

Mg 0.000000 0.000000 0.000000

H 0.000000 0.000000 1.731026

H 0.000000 0.000000 -1.731026

MgO

Mg 0.000000 0.000000 0.000000

O 0.000000 0.000000 1.749000

CH3MgCl

C -0.870088 0.000000 0.001030

Mg 1.212812 0.000000 -0.001760

Cl 3.437529 0.000000 -0.003337

H -1.259013 0.000000 1.030441

H -1.260570 -0.890943 -0.513187

H -1.260570 0.890943 -0.513187

**Mn.3p collection:**

HMnO4

Mn 0.102501 -0.331823 0.194980

H -0.266772 -2.476574 0.890242

O -1.382484 0.179330 0.399636

O 0.703957 0.206063 -1.162355

O 0.985575 0.125671 1.427454

O 0.111722 -2.071967 0.075043

MnO3Cl

Mn 0.395150 -0.463798 0.509135

O -0.389106 0.023281 -0.773057

O 0.921603 0.777359 1.332896

Cl -0.915434 -1.564244 1.719378

O 1.600887 -1.395532 0.092204

HMn(CO)5

Mn -0.040696 -0.547805 0.283156

O -1.393715 -3.023280 -0.647885

C -1.675709 0.151337 0.695672

C 0.159669 0.267343 -1.339928

C 0.740913 0.756238 1.293683

C -0.870765 -2.056885 -0.322656

H -0.211286 -1.258007 1.673816

O 0.286425 0.773489 -2.363039

O -2.699754 0.565113 1.001566

O 1.225465 1.542848 1.971562

C 1.542723 -1.455856 0.284176

O 2.521250 -2.050195 0.335555

H2MoO4

O -0.679109 1.359082 -0.832489

Mo 0.001216 0.024192 0.003337

O 0.837642 0.718139 1.330740

O -1.357803 -1.142715 0.610183

O 1.193724 -0.928168 -1.113491

H -2.077189 -0.839958 1.200595

H 1.979118 -0.503872 -1.514673

**Mo.4p collection:**

Mo(CO)6

O 1.567145 -2.559098 1.098236

C 1.004127 -1.641521 0.706112

Mo -0.000002 -0.000001 0.000002

C -1.004130 1.641520 -0.706108

O -1.567148 2.559096 -1.098234

C 1.769468 0.804873 -0.650109

O 2.757039 1.257533 -1.014232

C -1.769466 -0.804882 0.650111

O -2.757034 -1.257551 1.014233

C -0.241198 -0.926399 -1.812437

O -0.374567 -1.442306 -2.826864

C 0.241197 0.926408 1.812435

O 0.374570 1.442327 2.826855

MoSi2

Si -1.257294 0.897183 0.000000

Mo -0.000003 -0.938871 0.000000

Si 1.257298 0.897188 0.000000

MoS2

S -1.661507 0.712571 0.000000

Mo 0.000001 -0.569641 0.000000

S 1.661507 0.712570 0.000000

**Na.2p collection:**

Na2

Na 0.000000 0.000000 0.000000

Na 0.000000 0.000000 3.078900

NaCH3

Na -2.071509 -0.000061 -0.002972

C 0.246109 0.000048 0.000728

H 0.606760 -0.000003 1.040659

H 0.609401 0.899764 -0.519163

H 0.609239 -0.899749 -0.519151

NaOH

O 0.000000 0.000000 -1.025400

Na 0.000000 0.000000 0.924600

H 0.000000 0.000000 -1.966800

NaH

Na 0.000000 0.000000 0.000000

H 0.000000 0.000000 1.887400

**Nb.4p collection:**

Nb2O5

O 0.387599 -0.590620 -1.358106

Nb 1.226845 -0.139607 0.360010

O 2.934241 -0.043685 0.690147

O 0.138097 1.422463 0.346295

Nb -1.227761 0.004015 -0.344984

O -0.548016 -1.032877 1.083114

O -2.911005 -0.097890 -0.781075

NbCl5

Cl -2.185083 0.691787 -0.417024

Nb -0.000055 0.000776 0.001077

Cl 2.185132 -0.692210 0.415603

Cl 0.057280 1.305924 1.869173

Cl -0.710941 -2.160968 0.140538

Cl 0.653668 0.854691 -2.009367

NbOCl3

O 1.525910 0.805502 -0.825518

Nb 0.157725 0.082191 -0.087640

Cl -0.886621 1.711036 1.125470

Cl -1.173894 -0.709953 -1.765289

Cl 0.929280 -1.597476 1.253877

Nb(OCH3)5

Nb 0.334869 0.283700 -0.358497

O 2.189207 0.206832 -0.863587

C 3.316577 -0.481237 -0.446828

O -1.456792 0.075597 0.448877

C -1.806472 -0.164016 1.774887

O -0.519963 0.220224 -2.061663

C -1.869889 0.142131 -2.427482

O 0.794648 -1.278393 0.728301

C 0.060808 -2.463725 0.843476

O 0.499393 1.990519 0.513811

C -0.525823 2.865499 0.892895

H 4.018492 0.197067 0.071888

H 3.008037 -1.288786 0.250173

H 3.838194 -0.926260 -1.313472

H -0.991011 -0.686637 2.313131

H -2.000590 0.789284 2.305033

H -2.722156 -0.780843 1.834603

H -2.499387 0.046190 -1.520907

H -2.161319 1.050894 -2.982169

H -2.026351 -0.732838 -3.081631

H -1.016642 -2.301484 0.644137

H 0.434382 -3.222549 0.130616

H 0.169749 -2.873458 1.864495

H -0.522345 3.758602 0.242514

H -1.516829 2.376890 0.817856

H -0.361089 3.196797 1.933843

**N.1s collection:**

NH3

N 0.000000 0.000000 0.000000

H 0.000000 -0.937700 -0.381600

H 0.812100 0.468900 -0.381600

H -0.812100 0.468900 -0.381600

C2N2

C 0.000000 0.000000 0.695000

C 0.000000 0.000000 -0.695000

N 0.000000 0.000000 1.845000

N 0.000000 0.000000 -1.845000

N2O

N 0.000000 0.000000 -1.199800

N 0.000000 0.000000 -0.071600

O 0.000000 0.000000 1.112600

HCN

C 0.000000 0.000000 0.000000

H 0.000000 0.000000 1.064000

N 0.000000 0.000000 -1.156000

HNO2

H 1.754500 -0.182300 0.000000

O 0.894200 -0.606100 0.000000

N 0.000000 0.525200 0.000000

O -1.113500 0.169300 0.000000

HNO3

N 0.000000 0.155100 0.000000

O -0.267400 -1.225300 0.000000

O 1.174400 0.450400 0.000000

O -0.984400 0.839600 0.000000

H 0.619200 -1.603700 0.000000

N2H4

N 0.000000 0.723000 -0.112300

N 0.000000 -0.723000 -0.112300

H -0.447000 1.003100 0.756200

H 0.447000 -1.003100 0.756200

H 0.966300 1.003100 0.030100

H -0.966300 -1.003100 0.030100

**Ne.1s collection:**

Ne2

Ne -0.300511 0.000000 0.000000

Ne 2.300511 0.000000 0.000000

Ne(C6H6)

Ne -0.000018 0.000094 2.761751

C 0.693494 1.201168 -0.105048

C 1.386857 -0.000006 -0.105011

C -0.693490 1.201161 -0.105045

C 0.693499 -1.201186 -0.105092

C -1.386855 -0.000007 -0.105001

C -0.693493 -1.201180 -0.105090

H 2.481482 -0.000004 -0.105164

H 1.241067 2.148936 -0.105271

H -1.241067 2.148926 -0.105269

H 1.241074 -2.148953 -0.105306

H -1.241071 -2.148945 -0.105303

H -2.481480 -0.000005 -0.105151

NeCl2

Ne -1.324902 -0.000138 0.000000

Cl 1.662283 -1.001862 0.000000

Cl 1.662618 1.002000 0.000000

Ne

Ne 0.000000 0.000000 0.000000

**Ni.3p collection:**

Ni(CO)4

Ni 0.790012 1.019227 1.109043

C 0.629383 1.398821 -0.655340

C -0.605555 -0.001862 1.651918

C 0.793678 2.569871 2.049791

C 2.343389 0.135994 1.411601

O 0.527909 1.648270 -1.766506

O -1.487111 -0.640545 2.000987

O 3.325194 -0.415865 1.608541

O 0.795184 3.547534 2.642367

Ni(NH3)2(CN)2

Ni -0.051170 0.769868 0.992268

N 0.035823 -0.946534 1.786022

C 0.194728 -0.008107 -0.674789

C -0.293032 1.543477 2.654559

N -0.130982 2.471812 0.165477

N -0.451448 2.020446 3.707045

N 0.354707 -0.489736 -1.724731

H -0.086665 -0.896793 2.804811

H -0.694984 -1.540071 1.372888

H 0.944009 -1.374064 1.563083

H -0.868355 2.460339 -0.550922

H 0.762448 2.647289 -0.312465

H -0.313078 3.215776 0.849555

Ni(NH3)2(NO2)(ONO)

Ni -2.617422 0.270904 -2.265766

N -1.693257 -1.312530 -2.707517

N -2.620486 0.424138 -5.055903

N -2.861935 -0.372331 -0.518808

N -3.604956 1.819878 -1.812317

H -0.709609 -1.159109 -2.955504

H -2.198266 -1.687631 -3.528008

H -1.787768 -1.886015 -1.839069

H -4.485898 1.875033 -2.337345

H -3.071180 2.679031 -1.989551

H -3.777423 1.662489 -0.788229

O -2.473302 -1.507528 -0.212065

O -3.425243 0.344148 0.326671

O -3.105148 -0.693200 -4.977244

O -2.321407 1.028425 -3.947645

**O.1s collection:**

CO

C 0.000000 0.000000 0.000000

O 0.000000 0.000000 1.128000

H2O

O 0.000000 0.000000 0.000000

H 0.000000 0.759062 -0.587729

H 0.000000 -0.759062 -0.587729

O3

O 0.000000 0.000000 0.000000

O 0.000000 1.088500 0.669700

O 0.000000 -1.088500 0.669700

C6H4O2

H 0.000000 2.182973 1.259286

H 0.000000 -2.182973 1.259286

H 0.000000 2.182973 -1.259286

H 0.000000 -2.182973 -1.259286

C 0.000000 0.000000 1.441079

C 0.000000 0.000000 -1.441079

C 0.000000 1.266644 0.674582

C 0.000000 -1.266644 0.674582

C 0.000000 1.266644 -0.674582

C 0.000000 -1.266644 -0.674582

O 0.000000 0.000000 2.678518

O 0.000000 0.000000 -2.678518

CH3OH

C -0.050300 0.668500 0.000000

O -0.050300 -0.758500 0.000000

H -1.080700 1.041700 0.000000

H 0.465000 1.041700 0.892400

H 0.465000 1.041700 -0.892400

H 0.854400 -1.067700 0.000000

**Os.4f collection:**

Os(CH3)(CN)3 C3v

Os 0.000000 0.000000 -0.079204

C 0.000000 0.000000 -2.052649

C 1.755385 0.000000 0.648017

C -0.877692 1.520208 0.648017

C -0.877692 -1.520208 0.648017

H -1.056189 0.000000 -2.389828

H 0.528094 -0.914686 -2.389828

H 0.528094 0.914686 -2.389828

N -1.431137 2.478803 1.037151

N 2.862275 0.000000 1.037151

N -1.431137 -2.478803 1.037151

Os(NH3)4(SH)2

Os 0.102799 -0.040447 -0.194126

N 0.743969 1.925292 -0.099715

N -1.717444 0.759500 -0.768443

S -0.688579 -0.011807 2.020944

S 0.922067 -0.110560 -2.402636

N 1.992086 -0.860744 0.222381

N -0.592555 -2.022054 -0.120450

H -1.695828 1.791990 -0.909737

H -2.445925 0.596799 -0.043856

H 0.002746 2.619336 -0.335868

H 1.512545 2.111816 -0.772746

H 1.985316 -1.843095 0.547035

H 2.448210 -0.844544 -0.724241

H 0.143986 -2.748162 -0.145108

H -1.277250 -2.305735 -0.832135

H -1.995522 0.462413 1.915529

H 1.488834 1.144817 -2.604426

H 1.050470 2.172102 0.853169

H -2.038479 0.376186 -1.669318

H 2.584053 -0.356322 0.893549

H -1.042117 -2.061971 0.828114

Os(PH3)2(Cl)2(CHCH3)

Os 0.264056 -0.124964 0.144394

C -1.030705 0.833914 1.072940

C -2.406668 1.184618 0.673716

P 1.670039 0.162662 1.901969

P -1.051758 -0.505795 -1.722233

Cl 1.497505 1.348510 -1.145069

Cl 0.025287 -2.371303 0.648422

H -2.260596 -1.264298 -1.632189

H -0.434074 -1.262487 -2.767197

H -1.525060 0.576110 -2.528955

H 1.163378 0.899627 3.026571

H 2.898592 0.872212 1.710324

H 2.162091 -0.987559 2.597977

H -0.781073 1.220233 2.085329

H -3.129561 0.765325 1.400091

H -2.696094 0.835312 -0.331166

H -2.542184 2.282887 0.703689

Os2(CO)8 C4v

C 1.918091 0.000000 1.676463

C -1.918091 0.000000 1.676463

Os 0.000000 0.000000 1.447040

O -3.047736 0.000000 1.887728

O 3.047736 0.000000 1.887728

C 0.000000 1.918091 1.676463

C 0.000000 -1.918091 1.676463

O 0.000000 -3.047736 1.887728

O 0.000000 3.047736 1.887728

C 1.918058 0.000000 -1.676388

C -1.918058 0.000000 -1.676388

Os 0.000000 0.000000 -1.446749

O -3.047662 0.000000 -1.887875

O 3.047662 0.000000 -1.887875

C 0.000000 1.918058 -1.676388

C 0.000000 -1.918058 -1.676388

O 0.000000 -3.047662 -1.887875

O 0.000000 3.047662 -1.887875

Os(CO)2(N2)2(Cl)2 D2h

Os 0.000000 0.000000 0.000000

C 1.951117 0.000000 0.000000

Cl 0.000000 -2.400040 0.000000

O 3.089779 0.000000 0.000000

N 0.000000 0.000000 -1.944934

N 0.000000 0.000000 1.944934

Cl 0.000000 2.400040 0.000000

C -1.951117 0.000000 0.000000

N 0.000000 0.000000 3.054631

N 0.000000 0.000000 -3.054631

O -3.089779 0.000000 0.000000

OsO4 Td

O 0.993071 -0.993071 0.993071

Os 0.000000 0.000000 0.000000

O 0.993071 0.993071 -0.993071

O -0.993071 0.993071 0.993071

O -0.993071 -0.993071 -0.993071

**Pb.5d collection:**

PbCl2

Pb 0.000000 -0.259257 0.000000

Cl -2.041247 1.389629 0.000000

Cl 2.041247 1.389629 0.000000

PbF4

F 0.000000 -1.722800 -1.218200

Pb 0.000000 0.000000 0.000000

F 0.000000 1.722800 -1.218200

F -1.722800 0.000000 1.218200

F 1.722800 0.000000 1.218200

PbO2

Pb 0.000000 0.000002 0.000001

O -2.046628 -0.000001 0.000000

O 2.046628 -0.000001 0.000000

Pb(CH3)4

Pb -0.000010 0.000009 -0.000021

C 0.046162 1.862417 1.353140

C -0.036200 -1.895207 1.307157

C -1.883906 0.057491 -1.322688

C 1.874001 -0.024712 -1.337671

H 0.949779 1.817424 1.974300

H 0.062584 2.755073 0.714830

H -0.853288 1.856135 1.981902

H -0.935418 -1.865692 1.935607

H -0.057062 -2.771980 0.647287

H 0.867679 -1.904057 1.929530

H -1.848631 0.966168 -1.937075

H -1.887879 -0.836792 -1.958809

H -2.766260 0.068789 -0.670076

H 2.761166 -0.050581 -0.692050

H 1.834705 -0.918792 -1.972860

H 1.872679 0.884207 -1.952703

**P.2p collection:**

PH3

P -0.020166 -0.000731 -0.039724

H -0.614835 0.507505 1.165218

H 1.200678 0.708713 0.223209

H 0.466223 -1.162286 0.652097

H3PO4

P 0.009398 -0.720969 -0.026578

O -0.002442 -2.178826 -0.037144

O 1.466696 -0.049669 -0.041765

O -0.726562 -0.016699 -1.267988

O -0.701604 -0.040489 1.242145

H 1.555808 0.583630 -0.782283

H -1.446333 0.572229 -0.963994

H -0.085344 0.542482 1.729213

P(CH3)3

P -0.001082 0.000107 0.678100

C 1.579652 0.264966 -0.216119

C -0.560229 -1.501418 -0.216233

C -1.019816 1.236646 -0.216128

H 2.010427 1.238430 0.067245

H 1.435739 0.240874 -1.311585

H 2.303246 -0.516025 0.066947

H 0.068836 -2.359706 0.068454

H -0.508726 -1.365604 -1.311774

H -1.597946 -1.738613 0.067055

H -2.078065 1.121863 0.066979

H -0.926368 1.125009 -1.311604

H -0.705769 2.253471 0.068463

**Pd.4p collection:**

PdO2

O -1.744439 0.000031 0.000040

Pd 0.000000 0.000000 0.000000

O 1.744439 -0.000031 -0.000040

Pd(CH3)2(CN)2 C2v

Pd 0.000000 0.000000 1.438153

C -1.991348 0.000000 1.436569

C 0.000000 -1.505516 0.078250

C 0.000000 1.505516 0.078250

C 1.991348 0.000000 1.436569

N -3.155337 0.000000 1.436622

H 0.929725 2.042758 0.298431

H -0.929725 2.042758 0.298431

H 0.000000 1.054497 -0.921104

H 0.929725 -2.042758 0.298431

H 0.000000 -1.054497 -0.921104

H -0.929725 -2.042758 0.298431

N 3.155337 0.000000 1.436622

Pd(Cl)2(NH3)2 C2h

Pd 0.000000 0.000000 0.000000

N -2.022393 -0.001078 0.000000

H -2.357320 0.468986 0.851315

H -2.362289 -0.970114 0.000000

H -2.357320 0.468986 -0.851315

N 2.022393 0.001078 0.000000

H 2.357320 -0.468986 0.851315

H 2.362289 0.970114 0.000000

H 2.357320 -0.468986 -0.851315

Cl 0.000000 0.000000 2.295551

Cl 0.000000 0.000000 -2.295551

Pd(PH3)4 C4v

Pd 0.000000 0.000000 1.076781

P -2.464043 0.000000 1.232260

P 0.000000 2.464043 1.232260

P 2.464043 0.000000 1.232260

P 0.000000 -2.464043 1.232260

H -3.440377 -1.045465 1.030709

H -2.685892 0.000000 2.650939

H -3.440377 1.045465 1.030709

H -1.045465 3.440377 1.030709

H 1.045465 3.440377 1.030709

H 0.000000 2.685892 2.650939

H 1.045465 -3.440377 1.030709

H 0.000000 -2.685892 2.650939

H -1.045465 -3.440377 1.030709

H 3.440377 1.045465 1.030709

H 3.440377 -1.045465 1.030709

H 2.685892 0.000000 2.650939

Pd3(CO)6 D3h

Pd 1.647328 0.000000 0.000000

C 2.003407 0.000000 1.892700

O 2.232907 0.000000 3.011133

C 2.003407 0.000000 -1.892700

O 2.232907 0.000000 -3.011133

Pd -0.823664 1.426628 0.000000

C -1.001704 1.735001 1.892700

O -1.116454 1.933754 3.011133

C -1.001704 1.735001 -1.892700

O -1.116454 1.933754 -3.011133

Pd -0.823664 -1.426628 0.000000

C -1.001704 -1.735001 1.892700

O -1.116454 -1.933754 3.011133

C -1.001704 -1.735001 -1.892700

O -1.116454 -1.933754 -3.011133

Pd(C2H4)2(Cl)2 D2h

Pd 0.000000 0.000000 0.000000

C 0.000000 2.103351 0.683020

C 0.000000 2.103351 -0.683020

H -0.943118 2.177698 1.235505

H 0.943118 2.177698 1.235505

H 0.943118 2.177698 -1.235505

H -0.943118 2.177698 -1.235505

C 0.000000 -2.103351 0.683020

C 0.000000 -2.103351 -0.683020

H -0.943118 -2.177698 1.235505

H 0.943118 -2.177698 1.235505

H 0.943118 -2.177698 -1.235505

H -0.943118 -2.177698 -1.235505

Cl 2.298283 0.000000 0.000000

Cl -2.298283 0.000000 0.000000

PdS2

S -2.105440 0.000123 -0.000037

Pd 0.000000 0.000000 0.000000

S 2.105439 -0.000123 0.000037

PdSe2

Se -2.237670 -0.000112 0.000039

Pd 0.000000 0.000000 0.000000

Se 2.237669 0.000112 -0.000039

**Po.5d collection:**

PoO2

Po 1.104818 0.436972 0.000000

O 2.909070 1.213030 0.000000

O 1.334812 -1.513802 0.000000

PoCl2

Cl -1.907974 1.087634 0.000000

Po 0.000000 -0.499068 0.000000

Cl 1.907974 1.087634 0.000000

PoH2

Po -0.000076 -0.130228 0.000000

H -1.247782 1.146726 0.000000

H 1.247658 1.146702 0.000000

**Pt.4f collection:**

PtO2

O -1.713025 0.000027 0.000032

Pt 0.000000 0.000000 0.000000

O 1.713025 -0.000027 -0.000032

Pt(Cl)2(NH3)2 C2h

Pt 0.000000 0.000000 0.000000

N -2.015200 -0.000948 0.000000

H -2.360467 0.469844 0.848657

H -2.360195 -0.969832 0.000000

H -2.360467 0.469844 -0.848657

N 2.015200 0.000948 0.000000

H 2.360467 -0.469844 0.848657

H 2.360195 0.969832 0.000000

H 2.360467 -0.469844 -0.848657

Cl 0.000000 0.000000 2.289058

Cl 0.000000 0.000000 -2.289058

Pt(CH3)2(CN)2 C2v

Pt 0.000000 0.000000 1.411265

C -1.972539 0.000000 1.463072

C 0.000000 -1.520194 0.075727

C 0.000000 1.520194 0.075727

C 1.972539 0.000000 1.463072

N -3.136786 0.000000 1.510113

H 0.922377 2.085571 0.262695

H -0.922377 2.085571 0.262695

H 0.000000 1.084287 -0.933658

H 0.922377 -2.085571 0.262695

H 0.000000 -1.084287 -0.933658

H -0.922377 -2.085571 0.262695

N 3.136786 0.000000 1.510113

Pt(PH3)4 C4v

Pt 0.000000 0.000000 1.100462

P -2.412337 0.000000 1.240488

P 0.000000 2.412337 1.240488

P 2.412337 0.000000 1.240488

P 0.000000 -2.412337 1.240488

H -3.379525 -1.055174 1.021314

H -2.647438 0.000000 2.655580

H -3.379525 1.055174 1.021314

H -1.055174 3.379525 1.021314

H 1.055174 3.379525 1.021314

H 0.000000 2.647438 2.655580

H 1.055174 -3.379525 1.021314

H 0.000000 -2.647438 2.655580

H -1.055174 -3.379525 1.021314

H 3.379525 1.055174 1.021314

H 3.379525 -1.055174 1.021314

H 2.647438 0.000000 2.655580

Pt3(CO)6 D3h

Pt 1.618392 0.000000 0.000000

C 2.073677 0.000000 1.824799

O 2.466471 0.000000 2.902283

C 2.073677 0.000000 -1.824799

O 2.466471 0.000000 -2.902283

Pt -0.809196 1.401568 0.000000

C -1.036838 1.795857 1.824799

O -1.233236 2.136027 2.902283

C -1.036838 1.795857 -1.824799

O -1.233236 2.136027 -2.902283

Pt -0.809196 -1.401568 0.000000

C -1.036838 -1.795857 1.824799

O -1.233236 -2.136027 2.902283

C -1.036838 -1.795857 -1.824799

O -1.233236 -2.136027 -2.902283

PtS2

S -2.076229 0.000100 -0.000034

Pt 0.000000 0.000000 0.000000

S 2.076229 -0.000100 0.000034

PtSe2

Se -2.214683 -0.000122 0.000012

Pt -0.000001 0.000000 0.000000

Se 2.214683 0.000122 -0.000012

Pt(C2H4)2(Cl)2 D2h

Pt 0.000000 0.000000 0.000000

C 0.000000 2.075051 0.687988

C 0.000000 2.075051 -0.687988

H -0.940345 2.181982 1.239685

H 0.940345 2.181982 1.239685

H 0.940345 2.181982 -1.239685

H -0.940345 2.181982 -1.239685

C 0.000000 -2.075051 0.687988

C 0.000000 -2.075051 -0.687988

H -0.940345 -2.181982 1.239685

H 0.940345 -2.181982 1.239685

H 0.940345 -2.181982 -1.239685

H -0.940345 -2.181982 -1.239685

Cl 2.290960 0.000000 0.000000

Cl -2.290960 0.000000 0.000000

**Ra.5d collection:**

Ra(OH)2

Ra 0.003560 -0.006402 0.007619

O 2.279824 0.297584 -0.097935

H 3.178648 0.609784 0.065583

O -2.278210 -0.304463 0.093498

H -3.183822 -0.596503 -0.068766

RaCO3

Ra 4.220334 -0.251968 0.315950

O 6.155558 1.095589 0.155940

C 7.006829 0.070686 -0.065926

O 8.204314 0.211646 -0.217569

O 6.381865 -1.125553 -0.096894

RaSO4

Ra 2.781064 0.567827 -2.344919

O 3.242430 -0.829359 -4.435324

S 4.678152 -0.349524 -4.203874

O 4.521544 1.170962 -4.122329

O 4.939313 -0.744810 -2.748228

O 5.645397 -0.819996 -5.156027

**Rb.4p collection:**

RbH

Rb 0.000000 0.000000 -0.074944

H 0.000000 0.000000 2.317444

Rb2

Rb 0.000000 0.000000 -0.087216

Rb 0.000000 0.000000 3.992316

RbOH

Rb 0.000006 -0.000001 2.300953

O -0.000001 0.000000 -0.014717

H -0.000004 0.000001 -0.986737

RbCH3

Rb -2.562416 0.000000 -0.003094

C 0.237701 0.000000 -0.000303

H 0.610203 0.000000 1.038224

H 0.612256 0.899226 -0.517364

H 0.612256 -0.899226 -0.517364

**Re.4f collection:**

CH3ReO3

C 1.931091 0.258880 -0.617311

Re -0.008587 -0.000442 0.002587

O -1.004234 0.658771 -1.235249

O -0.229619 -1.695654 0.190964

O -0.158550 0.850806 1.489533

H 2.126552 1.335093 -0.747526

H 2.609661 -0.156706 0.144483

H 2.074386 -0.267248 -1.574481

HRe(CO)5

Re -0.044164 -0.561910 0.306559

O -1.464689 -3.146608 -0.725808

C -1.816315 0.216009 0.723641

C 0.173394 0.321483 -1.450083

C 0.810148 0.873399 1.369243

C -0.942440 -2.184824 -0.385890

H -0.233835 -1.337046 1.841047

O 0.300161 0.832585 -2.472427

O -2.839628 0.635487 1.024300

O 1.296658 1.668142 2.036336

C 1.681091 -1.532440 0.277978

O 2.664139 -2.119937 0.320783

HReO4

Re 0.099834 -0.313519 0.196696

H -0.259389 -2.677227 0.880250

O -1.493118 0.301220 0.405284

O 0.757134 0.272024 -1.279212

O 1.059039 0.234219 1.515381

O 0.090998 -2.186016 0.106602

ReO3Cl

Re 0.378778 -0.476954 0.524255

O -0.436770 0.072052 -0.883983

O 0.975761 0.884688 1.384031

Cl -1.011168 -1.645769 1.807564

O 1.706498 -1.456951 0.048690

ReF7

F 2.923008 -0.218480 0.389967

Re 1.232559 -0.086171 -0.379118

F 1.901807 1.645475 -0.634413

F 1.921987 -0.213959 -2.102507

F -0.285930 -1.002848 0.206328

F 1.637113 -1.901745 -0.537894

F -0.159817 0.734017 -1.302312

F 0.862055 0.701400 1.265342

Re(PH3)2(NH3)2(H2S)(CN)

Re 0.899558 -0.078941 -0.399297

N 0.823090 1.916361 0.384304

C -1.112063 -0.327501 -0.233976

H 0.690275 -2.179541 -2.195465

N -2.242164 -0.634897 -0.143921

S 3.207172 0.200866 -0.423942

N 0.658312 -2.123227 -1.166978

P 0.823606 0.603055 -2.581620

H -0.301768 -2.393770 -0.891921

H 2.077184 0.996592 -3.198853

H 0.035128 1.655735 -3.173986

P 0.949085 -0.853112 1.767977

H 0.572869 0.080031 2.815780

H 2.123540 -1.327677 2.453279

H 3.663918 0.528544 -1.698090

H 0.152136 2.505820 -0.122059

H 0.536111 1.943202 1.375626

H 1.753868 2.389755 0.330529

H 3.480693 1.564972 -0.055833

H 1.312539 -2.824572 -0.807340

H 0.472428 -0.368621 -3.600580

H 0.066363 -1.893541 2.218869

**Rh.4p collection:**

Rh(PH3)2(Cl)(CO)

Rh -0.055035 0.000005 -0.264107

Cl -0.169790 -0.000295 2.083918

H 1.054140 -2.750791 0.781957

C 0.073191 0.000972 -2.079128

H -1.113041 -2.818766 0.724654

H 0.036232 -3.198795 -1.081599

P -0.026024 -2.260337 -0.006622

P -0.015759 2.259355 -0.006691

O 0.168663 0.003396 -3.231790

H 0.071275 3.194459 -1.082849

H -1.111942 2.828237 0.702832

H 1.053270 2.744490 0.800026

Rh2(CO)8 D4h

C -1.912247 0.000000 -1.529644

C 0.000000 -1.912247 -1.529644

O -3.058601 0.000000 -1.568241

O 0.000000 -3.058601 -1.568241

Rh 0.000000 0.000000 -1.394223

C 0.000000 1.912247 -1.529644

O 0.000000 3.058601 -1.568241

C 1.912247 0.000000 -1.529644

O 3.058601 0.000000 -1.568241

C -1.912247 0.000000 1.529644

C 0.000000 -1.912247 1.529644

O -3.058601 0.000000 1.568241

O 0.000000 -3.058601 1.568241

Rh 0.000000 0.000000 1.394223

C 0.000000 1.912247 1.529644

O 0.000000 3.058601 1.568241

C 1.912247 0.000000 1.529644

O 3.058601 0.000000 1.568241

Rh(OH)3

Rh 0.897986 -0.827253 -0.709715

H -0.252071 -1.798276 1.029561

H 0.131179 -0.745703 -2.866146

H 2.220790 0.786581 0.255232

O -0.164044 -0.224385 -2.080181

O 2.472296 0.036425 -0.337422

O 0.316764 -2.216389 0.337569

Rh(SH)3

Rh 0.888347 -0.826351 -0.735841

H -0.406599 -1.856079 1.391240

H 0.090313 -0.836040 -3.309188

H 2.366550 1.040614 0.523258

S -0.402582 -0.059668 -2.286916

S 2.805173 0.093940 -0.373559

S 0.281698 -2.545415 0.419905

Rh(C2H4)2(CO)(Cl) C2v

Rh 0.000000 0.000000 -0.087297

Cl 0.000000 0.000000 2.232901

C 0.685426 2.115755 -0.002347

C -0.685426 2.115755 -0.002347

H 1.230306 2.111088 0.948034

H 1.254312 2.332220 -0.911975

H -1.230306 2.111088 0.948034

H -1.254312 2.332220 -0.911975

C 0.685426 -2.115755 -0.002347

C -0.685426 -2.115755 -0.002347

H 1.230306 -2.111088 0.948034

H 1.254312 -2.332220 -0.911975

H -1.230306 -2.111088 0.948034

H -1.254312 -2.332220 -0.911975

C 0.000000 0.000000 -1.907215

O 0.000000 0.000000 -3.063238

Rh(ONO)(NO2)(NH2)(NH3)2

Rh -0.355357 -0.173712 0.136812

N 0.034749 -1.388567 -1.451006

H -0.682529 -1.503963 -2.175883

H 0.189052 -2.257922 -0.894725

H 0.940353 -1.055124 -1.831928

N -0.583132 0.902181 1.880008

H -0.506414 0.242885 2.667429

H -1.464466 1.423303 1.968140

H 0.206768 1.560812 1.939807

O 1.568262 0.293535 0.343506

N 2.291922 0.079921 -0.763445

O 3.437838 0.421208 -0.640537

N -1.661441 1.001375 -0.877108

O -2.411980 0.053674 -0.401444

O -2.107706 1.901326 -1.537514

N 0.207128 -1.838549 1.120321

H 1.088434 -1.661000 1.625169

H -0.496783 -2.119683 1.816396

**Rn.5d collection:**

Rn2

Rn -0.183895 0.000000 0.000000

Rn 4.183895 0.000000 0.000000

RnF2

F -1.692201 -0.595314 1.171662

Rn -0.000002 -0.000001 0.000001

F 1.692203 0.595315 -1.171663

Rn(C6H6)

Rn -0.000035 0.000278 3.610941

C 0.693364 1.200931 -0.176056

C 1.386597 -0.000022 -0.176009

C -0.693357 1.200924 -0.176052

C 0.693370 -1.200980 -0.176142

C -1.386591 -0.000022 -0.176005

C -0.693360 -1.200973 -0.176134

H 2.481262 -0.000018 -0.175640

H 1.240958 2.148721 -0.175729

H -1.240957 2.148711 -0.175722

H 1.240965 -2.148770 -0.175918

H -1.240958 -2.148761 -0.175907

H -2.481256 -0.000020 -0.175629

**Ru.4p collection:**

Ru(PH3)2(Cl)2(CHCH3)

Ru 0.232437 -0.095677 0.165628

C -1.045590 0.852169 1.089983

C -2.410866 1.187228 0.673312

P 1.684986 0.159375 1.903952

P -1.046056 -0.512942 -1.734248

Cl 1.427232 1.411644 -1.133134

Cl -0.068255 -2.339530 0.684317

H -2.240786 -1.295769 -1.671592

H -0.373526 -1.273364 -2.739293

H -1.515714 0.543475 -2.576430

H 1.241382 0.881711 3.061774

H 2.919025 0.847199 1.685253

H 2.176664 -1.010667 2.562899

H -0.782885 1.228485 2.099649

H -3.136037 0.760259 1.394249

H -2.683143 0.836416 -0.334826

H -2.555694 2.284993 0.707120

Ru(CO)2(N2)2(Cl)2 D2h

Ru 0.000000 0.000000 0.000000

C 1.959557 0.000000 0.000000

Cl 0.000000 -2.402336 0.000000

O 3.094954 0.000000 0.000000

N 0.000000 0.000000 -1.960453

N 0.000000 0.000000 1.960453

Cl 0.000000 2.402336 0.000000

C -1.959557 0.000000 0.000000

N 0.000000 0.000000 3.066182

N 0.000000 0.000000 -3.066182

O -3.094954 0.000000 0.000000

RuO4 Td

O 0.989392 -0.989392 0.989392

Ru 0.000000 0.000000 0.000000

O 0.989392 0.989392 -0.989392

O -0.989392 0.989392 0.989392

O -0.989392 -0.989392 -0.989392

Ru(NH3)4(SH)2

Ru 0.094334 -0.025221 -0.196017

N 0.799620 1.931218 0.012774

N -1.747639 0.692962 -0.879518

S -0.471469 0.072264 2.110460

S 0.711722 -0.232884 -2.483089

N 2.020789 -0.778568 0.202643

N -0.675072 -1.985142 -0.114712

H -1.973486 1.666043 -0.627540

H -2.575133 0.140665 -0.614112

H 0.295351 2.657196 -0.516474

H 1.795641 2.071600 -0.208047

H 2.066327 -1.672428 0.710954

H 2.371586 -0.922796 -0.767901

H -0.007419 -2.743339 -0.310950

H -1.498725 -2.185127 -0.698406

H -1.758480 0.549470 2.180457

H 1.296203 0.958828 -2.840228

H 0.649489 2.104548 1.026492

H -1.623111 0.639977 -1.910160

H 2.669619 -0.148465 0.694080

H -0.956764 -2.035991 0.887210

Ru(CH3)(CN)3 C3v

Ru 0.000000 0.000000 -0.134260

C 0.000000 0.000000 -2.100866

C 1.729876 0.000000 0.651382

C -0.864938 1.498117 0.651382

C -0.864938 -1.498117 0.651382

H -1.063851 0.000000 -2.403745

H 0.531925 -0.921322 -2.403745

H 0.531925 0.921322 -2.403745

N -1.408941 2.440357 1.082127

N 2.817882 0.000000 1.082127

N -1.408941 -2.440357 1.082127

Ru3(CO)3 C3v

Ru 1.438136 0.000000 -0.886139

C 1.714387 0.000000 0.869689

O 1.922692 0.000000 2.016449

Ru -0.719068 1.245462 -0.886139

C -0.857194 1.484703 0.869689

O -0.961346 1.665100 2.016449

Ru -0.719068 -1.245462 -0.886139

C -0.857194 -1.484703 0.869689

O -0.961346 -1.665100 2.016449

**Sb.4d collection:**

SbF5

F -1.915791 -0.000889 0.000000

Sb 0.000000 0.002381 0.000000

F 1.915791 -0.000889 0.000000

F 0.000000 1.909703 0.000000

F 0.000000 -0.955153 1.650366

F 0.000000 -0.955153 -1.650366

SbH3

Sb 0.001001 0.000518 -0.304906

H -1.417454 -0.029993 0.698763

H 0.683370 1.244211 0.697947

H 0.733082 -1.214736 0.698296

SbH3O4

O 3.713305 -1.826953 -0.697263

Sb 3.591257 -0.045821 0.187785

O 1.736571 0.082906 0.865628

O 4.948349 0.325712 1.384280

O 3.257885 0.961327 -1.495352

H 4.406080 -2.338016 -0.223178

H 3.564255 0.374710 -2.222437

H 1.194398 0.358235 0.092337

Sb2S3

S -3.719424 0.828331 0.000000

Sb -1.907075 -0.543735 0.000000

S 0.000000 1.078108 0.000000

Sb 1.907075 -0.543735 0.000000

S 3.719424 0.828331 0.000000

**S.2p collection:**

H2S

S 0.000000 0.000000 0.103000

H 0.000000 0.961600 -0.823900

H 0.000000 -0.961600 -0.823900

CS2

C 0.000000 0.000000 0.000000

S 0.000000 0.000000 1.554000

S 0.000000 0.000000 -1.554000

SO2

S 0.000000 0.000000 0.000000

O 0.000000 1.237100 0.721500

O 0.000000 -1.237100 0.721500

H2SO4

S 0.000000 0.000000 0.124800

O 1.251000 -0.034900 0.800000

O -1.251000 0.034900 0.800000

O 0.000000 -1.217200 -0.873200

O 0.000000 1.217200 -0.873200

H -0.354800 1.993300 -0.412100

H 0.354800 -1.993300 -0.412100

**Sc.3p collection:**

ScH3

H -1.537332 0.887790 0.109431

Sc -0.000002 -0.000262 -0.326149

H 1.537331 0.887787 0.109431

H 0.000002 -1.775315 0.109387

ScF3

F -1.597694 0.921813 0.002641

Sc 0.000001 0.000406 -0.005823

F 1.597693 0.921813 0.002641

F 0.000000 -1.844033 0.002641

Sc2O3

O -0.781453 0.625665 -0.817100

Sc 0.056537 -1.072767 -1.129909

O 1.400156 -0.005093 -1.993339

Sc 1.050494 1.064443 -0.436439

O 1.042577 -0.634756 0.459769

**Se.3d collection:**

H2Se

Se 0.000000 0.000000 0.056900

H 0.000000 1.040500 -0.967300

H 0.000000 -1.040500 -0.967300

SeO2

Se 0.000000 0.000000 0.280700

O 0.000000 1.346400 -0.596500

O 0.000000 -1.346400 -0.596500

CSe

C 0.000000 0.000000 0.000000

Se 0.000000 0.000000 1.699665

**Si.2p collection:**

SiH4

Si 0.000000 0.000000 0.000000

H 0.854400 0.854400 0.854400

H -0.854400 -0.854400 0.854400

H -0.854400 0.854400 -0.854400

H 0.854400 -0.854400 -0.854400

SiO

Si 0.000000 0.000000 0.000000

O 0.000000 0.000000 1.509700

H4SiO4

Si -0.003099 -0.177667 0.000047

O 0.014213 1.448742 -0.000073

O -1.599497 -0.477231 0.000148

O 0.717546 -0.782412 1.347770

O 0.717003 -0.783042 -1.347688

H 0.890174 1.871255 -0.000447

H -1.869090 -1.411423 0.000264

H 1.480606 -1.375602 1.254756

H 1.481944 -1.373821 -1.254777

SiH3CH3

C 0.000000 0.000000 -1.236700

Si 0.000000 0.000000 0.631900

H 0.000000 -1.023700 -1.627200

H -0.886600 0.511900 -1.627200

H 0.886600 0.511900 -1.627200

H 0.000000 1.389300 1.151400

H -1.203100 -0.694600 1.151400

H 1.203100 -0.694600 1.151400

**Sn.4d collection:**

SnO2

Sn 0.000000 0.000000 0.000000

O -1.865127 0.000000 0.000000

O 1.865127 0.000000 0.000000

SnF4

Sn 0.000000 0.000000 0.000000

F 0.000000 -1.578701 -1.115778

F 0.000000 1.578701 -1.115778

F -1.578701 0.000000 1.115778

F 1.578701 0.000000 1.115778

Sn(CH3)4

Sn -0.000431 0.000038 -0.000180

C 0.047956 1.765477 1.281799

C -0.038268 -1.796965 1.237656

C -1.786491 0.058729 -1.252804

C 1.776614 -0.027264 -1.266757

H 0.945744 1.743893 1.915097

H 0.066041 2.675576 0.666084

H -0.843154 1.785903 1.924517

H -0.931582 -1.792203 1.877614

H -0.059699 -2.691554 0.599708

H 0.857389 -1.832178 1.873418

H -1.774969 0.961329 -1.879495

H -1.817490 -0.827463 -1.901890

H -2.686475 0.072168 -0.622278

H 2.681082 -0.053209 -0.643060

H 1.762102 -0.915698 -1.913313

H 1.801631 0.873220 -1.896117

SnCl2

Sn 0.000000 -0.268110 0.000000

Cl -1.831787 1.314055 0.000000

Cl 1.831787 1.314055 0.000000

**Sr.4p collection:**

Sr(OH)2

Sr 1.385562 0.744248 -2.055385

O 3.521622 1.082117 -1.687563

O -0.634844 -0.090680 -2.244019

H 4.469368 1.174384 -1.506550

H -1.505412 -0.512403 -2.306149

SrF2

Sr 2.905895 5.440133 5.625664

F 0.941636 5.876196 4.893709

F 3.719647 4.855802 7.517916

SrCO3

Sr 2.682845 2.885394 2.249839

O 3.526001 1.526259 0.745149

O 1.482540 1.156411 1.615709

C 2.432598 0.732592 0.754214

O 2.316400 -0.257485 0.058711

**Ta.4f collection:**

TaF5

F -0.463771 -0.471620 1.781734

Ta 0.002553 -0.003149 0.000659

F 0.444252 0.499016 -1.777705

F 0.811505 1.554659 0.629705

F 1.008289 -1.561972 -0.170718

F -1.802828 -0.016935 -0.463676

Ta(CH3)5

C 1.449031 0.131265 -1.475245

Ta 0.122583 0.013867 0.216191

C -2.008087 -0.218608 0.449528

C -0.064352 -2.017114 -0.475464

C 1.146822 0.183980 2.073783

C -0.512468 1.921584 -0.557401

H 1.937955 1.121408 -1.510882

H 2.234570 -0.634754 -1.324339

H 0.976691 -0.058224 -2.452984

H -2.231265 -1.161405 0.981437

H -2.375583 0.622201 1.067970

H -2.574688 -0.212800 -0.496173

H -0.539904 -2.604411 0.333322

H -0.673826 -2.140051 -1.385629

H 0.937043 -2.443936 -0.666948

H 2.069428 0.785430 1.966859

H 0.511414 0.678115 2.833281

H 1.436301 -0.806534 2.472749

H -1.231178 2.391208 0.138335

H 0.372900 2.580243 -0.635327

H -0.983388 1.868434 -1.553162

Ta(NH2)5

Ta -0.853028 0.690672 -0.721458

N 0.978284 1.411688 -0.302500

N -0.896130 -0.864140 -1.950140

N -0.891581 2.229103 -2.046513

N -0.795610 -0.236872 1.085502

N -2.646091 1.329446 -0.068761

H 1.441860 1.284319 0.596904

H 1.432587 2.123892 -0.876283

H -1.712128 -1.460272 -2.101210

H -0.108249 -1.175161 -2.521321

H -0.683238 3.213285 -1.866696

H -1.385990 2.140475 -2.938431

H -0.969697 0.184660 2.000101

H -0.316142 -1.133179 1.206852

H -3.091191 0.959151 0.772556

H -3.085063 2.192159 -0.390351

Ta2O5

O -0.552001 -0.711968 -1.305958

Ta 1.157345 -0.075967 -0.509228

O 2.774103 -0.051750 -1.203267

O 0.048556 1.440675 0.136005

Ta -1.168838 -0.098268 0.482264

O 0.485674 -0.990506 1.127429

O -2.786537 -0.115716 1.174154

Ta2S5

S -0.716733 -0.901165 -1.700370

Ta 1.201981 -0.073070 -0.530952

S 3.191219 -0.053666 -1.377927

S 0.057240 1.905193 0.185782

Ta -1.214640 -0.099082 0.501309

S 0.641305 -1.262158 1.470774

S -3.202071 -0.119553 1.352784

**Tc.4p collection:**

HTcO4

O -0.156433 0.819628 -1.669472

Tc -0.003206 0.003720 0.012048

O -0.687608 0.984438 1.232593

O -0.825837 -1.489323 -0.066694

O 1.641068 -0.257853 0.398036

H 0.322617 1.672389 -1.762112

HTc(CO)5

Tc -0.049197 -0.571645 0.316410

O -1.466022 -3.153238 -0.726919

C -1.819799 0.215568 0.730434

C 0.174316 0.325912 -1.458607

C 0.808503 0.868965 1.373011

C -0.946738 -2.194229 -0.381734

H -0.235169 -1.334779 1.825895

O 0.305664 0.839843 -2.474951

O -2.837363 0.646128 1.026822

O 1.297474 1.667297 2.030345

C 1.682502 -1.535002 0.283347

O 2.670349 -2.110482 0.321626

TcO3Cl

Tc 0.383517 -0.471163 0.517249

O -0.434495 0.068671 -0.877876

O 0.970972 0.875423 1.382873

Cl -1.004600 -1.641863 1.804588

O 1.697706 -1.454002 0.053723

**Te.4d collection:**

HTeOF5

F -0.824412 1.663314 -0.289443

Te 0.047142 -0.015613 -0.030176

F 0.747496 -1.763289 0.290065

F 0.410943 0.460012 1.798647

F -0.275001 -0.468287 -1.847396

F -1.650430 -0.708421 0.475230

O 1.787792 0.698082 -0.528798

H 2.174571 1.022402 0.321371

TeH2

Te 0.000000 0.000000 0.064418

H 0.000000 1.183506 -1.132509

H 0.000000 -1.183506 -1.132509

H2TeO3

O 1.518127 -0.881768 -0.009600

Te 0.000623 0.179643 0.758672

O 0.008563 1.573409 -0.438520

O -1.526819 -0.879794 0.005705

H 1.692380 -0.434433 -0.872011

H -1.695574 -0.459357 -0.870546

Te(CH3)2

C 1.574414 -1.012655 0.000268

Te -0.003621 0.481492 0.002615

C -1.579146 -1.015255 -0.001434

H 1.490461 -1.623175 0.906668

H 1.487760 -1.622940 -0.906036

H 2.527253 -0.468790 -0.001063

H -1.490373 -1.625656 -0.907469

H -1.495242 -1.625485 0.905177

H -2.532907 -0.473035 -0.004026

**Ti.3p collection:**

H2TiO3

Ti 0.177676 0.546751 1.466726

O 1.431789 0.421767 0.116100

O -1.321399 0.230647 0.896846

O 0.234964 2.013380 2.567555

H 2.278496 0.907562 0.173401

H -0.571255 2.521518 2.784440

TiO2

Ti 1.350255 1.008783 0.215577

O 0.032115 0.012261 0.106484

O 2.671586 0.206006 -0.378663

TiH4

Ti 1.380297 0.961862 0.572216

H 0.519330 0.383462 1.915875

H 2.535098 0.017981 1.383295

H 0.014944 0.190788 -0.063166

H 1.927657 -0.159667 -0.570266

Ti(CH3)4

Ti 5.684011 -2.139424 3.460522

C 7.695125 -2.139501 3.922719

C 5.520061 -2.139419 1.404008

C 4.816297 -0.433774 4.213235

C 4.816176 -3.845041 4.213170

H 7.854137 -2.139369 5.015348

H 8.163197 -1.239234 3.485708

H 8.163073 -3.039945 3.485940

H 4.449941 -2.140015 1.128431

H 6.003670 -3.040429 0.987249

H 6.002663 -1.237859 0.987275

H 3.985787 -0.675660 4.899097

H 4.439382 0.183356 3.378266

H 5.593915 0.128535 4.761821

H 3.985455 -3.603128 4.898769

H 5.593663 -4.407262 4.762032

H 4.439530 -4.462265 3.378149

**Tl.5p collection:**

Tl2O3

O 1.492191 0.672411 0.020926

Tl 3.192412 -0.125509 -0.002535

O 4.952389 -1.120370 -0.032396

Tl 6.712537 -0.125189 -0.002524

O 8.412872 0.672557 0.020929

TlH3

Tl 0.000000 0.000015 0.005669

H -1.516293 0.875428 -0.000090

H 1.516293 0.875428 -0.000090

H 0.000000 -1.750870 -0.000090

TlCl

Cl -2.199574 0.000000 0.000000

Tl 0.305574 0.000000 0.000000

**V.3p collection:**

H3VO4

V -0.967756 -0.153720 0.936710

O 0.807637 -0.067805 0.744060

O -1.604415 -1.568506 0.061767

O -1.473390 -0.240822 2.649855

O -1.560149 1.198047 0.351129

H 1.298320 -0.872423 1.014697

H -2.251514 -1.391916 -0.652695

H -1.298433 -1.099254 3.091076

VOCl3

V 0.493923 -0.150876 -0.166036

Cl 2.533166 0.160046 0.395096

Cl -0.457171 1.699001 -0.659562

Cl -0.575605 -1.163140 1.384099

O 0.474350 -1.069867 -1.450821

V2O5

O -0.864770 2.412491 4.840165

V 0.538893 2.725610 5.544815

O 1.906911 1.943288 4.915696

O 1.525839 4.332642 5.630991

V 2.638413 3.202400 6.430284

O 1.332816 2.475550 7.317662

O 3.911692 3.732520 7.240108

**W.4f collection:**

H2WO4

O -1.777964 -3.395857 -0.600147

W -0.599801 -1.917360 -0.520309

O 1.038236 -2.468166 -0.465648

O -0.900818 -0.846912 1.010616

H -1.446820 -4.281337 -0.347616

O -0.831872 -0.978366 -1.953418

H -1.390161 -0.004902 0.916420

WO3

W 1.571317 0.402236 -0.837562

O 3.012328 -0.221626 -0.093488

O 1.565435 -0.244644 -2.450168

O 0.252773 -0.380248 -0.019996

W(CO)6

W 2.189449 -2.449687 -2.975131

C 0.358435 -1.695078 -2.490699

O -0.671929 -1.267673 -2.220226

C 2.184295 -3.558383 -1.264306

C 3.086167 -0.913614 -1.978698

C 1.292728 -3.985910 -3.971330

C 2.194599 -1.341142 -4.686055

O 2.180919 -4.183654 -0.301893

C 4.020552 -3.204032 -3.459618

O 3.590941 -0.050367 -1.415195

O 2.197952 -0.716074 -5.648596

O 0.787972 -4.849364 -4.534533

O 5.051053 -3.631092 -3.730110

**Xe.4d collection:**

Xe2

Xe -0.123332 0.000000 0.000000

Xe 4.123332 0.000000 0.000000

Xe(C6H6)

Xe -0.000033 0.000202 3.544798

C 0.693368 1.200933 -0.170561

C 1.386616 -0.000016 -0.170525

C -0.693364 1.200929 -0.170550

C 0.693377 -1.200972 -0.170615

C -1.386611 -0.000016 -0.170517

C -0.693365 -1.200963 -0.170616

H 2.481166 -0.000016 -0.170136

H 1.240885 2.148640 -0.170232

H -1.240887 2.148630 -0.170215

H 1.240900 -2.148674 -0.170351

H -1.240893 -2.148663 -0.170353

H -2.481159 -0.000014 -0.170129

XeO4

O -1.295488 0.040071 -1.327230

Xe -0.003163 -0.000249 -0.004174

O -0.002181 1.605722 0.916751

O -0.362461 -1.378005 1.178708

O 1.663293 -0.267540 -0.764055

XeF2

F -1.640090 -0.577014 1.135610

Xe 0.000000 0.000000 0.000000

F 1.640089 0.577014 -1.135609

XeF4

F -0.900545 0.204000 1.818813

Xe -0.000003 -0.000002 -0.000003

F 0.900562 -0.203991 -1.818809

F 1.658889 0.946884 0.715212

F -1.658903 -0.946891 -0.715213

XeF6

F -0.742975 -1.215149 1.423893

Xe -0.000001 -0.000014 -0.000002

F 0.742910 1.215159 -1.423894

F 1.847165 -0.724191 0.346505

F -1.847150 0.724215 -0.346479

F -0.302424 -1.433979 -1.381404

F 0.302477 1.433959 1.381381

**Y.4p collection:**

YF3

F -1.726784 0.995425 0.047407

Y 0.000027 0.001027 -0.139732

F 1.726792 0.995471 0.047394

F -0.000035 -1.991923 0.047031

Y2O3

O -0.845598 0.680614 -0.807924

Y -0.012758 -1.174143 -1.178336

O 1.443651 -0.005306 -2.063061

Y 1.119493 1.165423 -0.387811

O 1.063523 -0.689097 0.520114

YN

Y -0.123800 0.000000 0.000000

N 1.723800 0.000000 0.000000

**Zn.3p collection:**

ZnO

Zn 2.552137 -0.895110 -0.418033

O 2.481063 -0.245232 -1.978711

ZnCl2

Zn 3.186273 -0.779198 -0.696184

Cl 4.306582 -1.458522 0.925609

Cl 2.066856 -0.102587 -2.319689

Zn(CH3)2

Zn 2.645243 -1.052248 -0.927541

C 4.318604 -1.053899 0.040596

C 0.969931 -1.053969 -1.892608

H 4.141079 -1.054010 1.127472

H 4.915703 -0.162998 -0.210625

H 4.913008 -1.946499 -0.211127

H 0.116917 -1.054619 -1.195795

H 0.890817 -1.946163 -2.533836

H 0.888683 -0.162641 -2.534754

**Zr.4p collection:**

Zr(CH3)4

Zr 5.690383 -2.139417 3.442642

C 7.844906 -2.139543 3.963787

C 5.496397 -2.139417 1.234776

C 4.754897 -0.320008 4.272117

C 4.754719 -3.958769 4.272044

H 7.997336 -2.139317 5.058838

H 8.348777 -1.245827 3.551307

H 8.348598 -3.033540 3.551699

H 4.432489 -2.140373 0.932853

H 5.974952 -3.033132 0.793589

H 5.973323 -1.244786 0.793683

H 3.929289 -0.552709 4.969456

H 4.352619 0.319036 3.463734

H 5.513052 0.267916 4.824149

H 3.928966 -3.726036 4.969199

H 5.512767 -4.546657 4.824260

H 4.352613 -4.597854 3.463607

ZrH4

Zr 1.386849 1.017379 0.554310

H 0.478887 0.386517 2.022319

H 2.620180 -0.001580 1.459768

H -0.106153 0.188399 -0.118294

H 1.997563 -0.196290 -0.680149

ZrO2

Zr 1.350182 1.079037 0.243028

O -0.057163 -0.029441 0.109167

O 2.760937 0.177455 -0.408797

**Silver (TZP, 4p frozen)**

BASIS

1S 30.800

2S 20.300

3S 9.550

4S 5.950

2P 21.100

3P 10.100

4P 4.450

3D 11.650

5S 2.600

5S 1.550

5S .950

4D 4.750

4D 2.600

4D 1.380

5P 1.550

END

CORE 4 3 1 0

1S 310.000

1S 78.300

1S 47.000

2S 54.150

2S 20.100

3S 10.450

3S 6.850

4S 6.350

4S 4.050

5S 2.600

5S 1.550

5S .950

2P 29.850

2P 19.250

3P 11.600

3P 8.400

4P 6.150

4P 4.150

4P 2.900

3D 18.300

3D 10.950

3D 7.250

4D 4.750

4D 2.600

4D 1.380

END

DESCRIPTION

.93427515040237756744E-02 .79161714874711963663E-01

.94293409930058114110E+00 -.20824595347993083566E-01

.10611231075810122117E-02 -.18073619252765875040E-02

.29608835192324002225E-02 -.21368405611759937884E-02

.61696413207212798431E-03 -.21451446434727752051E-03

.11122959122939918037E-03 -.40631203839249601902E-04

-.25253038496165823926E-02 -.13937756140515417114E+00

-.68235910164350252471E-01 -.25078696570339875649E+00

.11160443945104474572E+01 -.19309937892559934491E-02

.43185486058788177810E-02 -.23734884948556625103E-02

.29491467336081455142E-03 -.10954330965657048407E-04

-.13120692865570931826E-04 .75261819002624997737E-05

.61554821562137247790E-03 .13994077665616730388E+00

-.13421321931555943441E+00 .23760469669870643017E+00

-.65682481791913649438E+00 .10594460078740253195E+01

.16479388430216579398E+00 -.84091056877724779106E-01

.27675721468335155728E-01 -.11150250961868087354E-01

.61573801720435899359E-02 -.23127352546557142753E-02

-.20689878375675221705E-03 -.71540403708801256211E-01

.79869917647607716060E-01 -.12038644451406062263E+00

.30683797580385269166E+00 -.52331414417081212331E+00

-.38985339757169990005E+00 .10426509505339320860E+01

.40095001231969418232E+00 .12626580041728082890E-01

-.68554640393270454418E-02 .27975626435307221658E-02

.25314091014024653337E+00 .76716444691571228365E+00

.66048032326003740938E-02 -.76871511001935398030E-02

.60390255453612673789E-02 -.44671886616956231822E-02

.16462490544650375344E-02

-.85589490756034183883E-01 -.42824599079174208160E+00

.65048787451645606605E+00 .51028794935868360128E+00

-.14392610750236015799E-01 .96187397489979151871E-02

-.33134461854683557903E-02

.32149574228818214583E-01 .18567618923669307329E+00

-.29809629318072944359E+00 -.33796668083798508952E+00

.59979061319826942889E+00 .50985845374814497966E+00

.12706796926511096379E+00

.14040676769022453207E+00 .58374637726637235158E+00

.34309374242289297774E+00 -.12638261652646069159E-02

.77845280288926365243E-03 -.23174213935337883031E-03

0/

END

FIT

7S 8.55

7S 6.76

8S 6.11

9S 3.55

9S 2.34

7P 7.55

8P 4.29

9P 3.45

9P 2.50

6D 9.89

7D 7.28

8D 3.51

8D 2.33

END

**Aluminium (TZP, 2p frozen)**

BASIS

1S 11.50

2S 4.10

2P 4.65

3S 0.95

3S 1.35

3S 2.00

3P 0.60

3P 0.95

3P 1.50

3D 1.60

END

CORE 2 1 0 0

1S 11.5000

1S 15.9000

2S 3.3000

2S 4.8000

3S 0.9500

3S 1.3500

3S 2.0000

2P 3.2000

2P 6.3000

3P 0.5800

3P 0.9500

3P 1.6500

END

DESCRIPTION

.74617226491287536039E+00 .26024478454017924145E+00

-.63176603906744174116E-02 .63563159354031052023E-02

.14533558888029966209E-02 -.37704972683678377593E-02

.40974221329893416310E-02

-.35440559336582833083E+00 .32428893835932955103E-01

.40421616009395122671E+00 .68296731874671490203E+00

-.29740510441851148934E-02 .70442802580953055303E-02

-.67280909143557950103E-02

.63895151844658615303E+00 .42738003267475860314E+00

-.44761169084379260480E-02 .10063442288389928825E-01

-.73449010974815914093E-02

0/

END

FIT

4S 4.23

5S 2.43

5S 1.71

5S 1.20

4P 8.86

5P 2.43

5P 1.51

4D 4.85

5D 3.43

5D 1.20

END

**Argon (TZP, 2p frozen)**

BASIS

1S 14.70

2S 6.05

2P 7.10

3S 1.30

3S 2.10

3S 3.35

3P 1.20

3P 1.90

3P 3.00

3D 2.200

END

CORE 2 1 0 0

1S 21.9000

1S 16.3000

2S 6.6500

2S 4.7500

3S 1.7500

3S 2.6000

3S 4.1000

2P 9.3000

2P 5.4500

3P 1.2000

3P 1.9000

3P 3.2000

END

DESCRIPTION

.26317348226030784497E+00 .74039887765646672513E+00

.28999611060854049022E-01 -.39757704838006197035E-01

.32232609636603584691E-02 -.99772178318447059947E-02

.22935850106690695155E-01

.55882258804981807532E-01 -.40959264427498354166E+00

.91204009827694587642E+00 .19773537550869277335E+00

-.89198569322790927788E-02 .23966984512071042024E-01

-.43221269542186052659E-01

.40179995460197687196E+00 .63722591477277668037E+00

.19599319281012831523E-02 -.39161588143862106903E-02

.48415658337450989332E-02

0/

END

FIT

3S 11.77

4S 10.47

5S 3.30

5S 2.40

5P 4.92

5P 3.00

END

**Arsenic (TZP, 3d frozen)**

BASIS

1S 23.55

2S 12.70

3S 6.55

2P 15.15

3P 6.15

3D 4.53

4S 1.50

4S 2.30

4S 3.30

4P 1.00

4P 1.70

4P 2.85

4D 1.6

END

CORE 3 2 1 0

1S 30.8500

1S 39.0500

2S 13.0000

3S 4.5000

3S 6.7000

4S 1.5000

4S 2.3000

4S 3.5500

2P 12.2500

2P 18.4500

3P 4.5000

3P 7.2500

4P 1.0000

4P 1.7000

4P 2.9000

3D 2.8500

3D 5.2500

3D 9.8000

END

DESCRIPTION

.62483952630577876963E+00 .37610674958196282924E+00

.12001576985213965876E-01 .24148279378428554043E-01

-.19559769865605560607E-01 -.26793195050208704934E-02

.83597271688812362089E-02 -.16164531488641845081E-01

-.48309746395440583688E+00 .85330579533119649271E-01

.11138193388503292436E+01 .75633595976745654488E-01

-.66543321631230958890E-01 -.74269237098786959617E-02

.23568860461816543844E-01 -.47410939647120044216E-01

.24885032535344839277E+00 -.75041180753853561902E-01

-.57424158142221615186E+00 .17750338357386127863E+00

.96293837207003718515E+00 .46832236201276820908E-02

-.15716093878250750326E-01 .31288052407416673539E-01

.64671688489427914259E+00 .37357031631692516616E+00

-.79302052357569322721E-02 .10319929663264681280E-01

.95099313109673407497E-03 -.25966395966205012454E-02

.43491537964431429067E-02

-.31408467240052162506E+00 -.12945078617831770607E+00

.47023140963934778602E+00 .67813987341924020225E+00

-.97235883130819848394E-03 .29491839461874805491E-02

-.40259919092260195925E-02

.27558596930976358985E+00 .58115490192663166180E+00

.27942695350821517009E+00

0/

END

FIT

7S 4.80

7P 3.96

7D 4.31

END

**Astatine (TZP, 5d frozen)**

BASIS

1S 63.000

2S 39.700

3S 23.400

4S 13.700

5S 8.150

2P 42.850

3P 19.900

4P 13.650

5P 7.200

3D 24.600

4D 12.250

5D 4.600

4F 9.850

1S 211.300

6S 4.250

6S 2.850

6S 1.940

6P 3.300

6P 2.050

6P 1.280

END

CORE 5 4 3 1

1S 889.000

1S 211.300

1S 97.000

2S 113.200

2S 42.250

3S 53.700

3S 23.050

4S 14.050

4S 10.000

5S 8.400

5S 5.800

6S 4.250

6S 2.850

6S 1.940

2P 74.600

2P 40.050

3P 22.700

3P 16.500

4P 15.500

4P 10.600

5P 7.200

5P 4.800

6P 3.300

6P 2.050

6P 1.280

3D 30.800

3D 19.800

4D 13.750

4D 9.100

5D 6.650

5D 4.300

5D 2.800

4F 18.950

4F 11.000

4F 6.600

END

DESCRIPTION

.25385987391806585922E-01 .16072113949532884636E+00

.84771579977356192881E+00 .21397690373946225140E-01

-.47045212517866349944E-01 .41284218603691892613E-01

.12991991458086700559E-02 -.63166923325218087525E-03

.52568721162177267387E-03 -.23335051416610360785E-03

.62786511420799120194E-04 -.13482269775513708345E-04

.30998586757558641209E-05 -.43117899703562603530E-06

-.86398090775832037808E-02 -.63411494985378277778E-01

-.26632125174527970790E+00 -.83596550262576549417E-01

.10044649698609473543E+01 .86581613970619905607E-01

.13325738742235436907E-02 .50067402042965614140E-02

-.78748970412072863412E-02 .63194862188114776444E-02

-.33546388686329958913E-02 .15796334544759102394E-02

-.81653153375137968884E-03 .27095611285319758403E-03

.42070819499094944810E-02 .20889999359540445401E-01

.15275635895531275432E+00 -.36714230101847268074E-02

-.34727034220422614563E+00 -.34419769612318035223E+00

.11941305998991538218E+01 .29890388098805947305E-02

-.42197708433683225188E-02 .61649076356959514600E-02

-.44434757467348377086E-02 .24521228767516666490E-02

-.13627004702669275049E-02 .46795339746731438868E-03

-.22309181435937598496E-02 -.58012813530493563888E-02

-.93174660197685110763E-01 .25346606089375472992E-01

.96289250038805507259E-01 .29393854158555943190E+00

-.89494372882840367289E+00 .10948335315207897711E+01

.19167414180646097366E+00 -.29594094728613493800E-01

.17408243165266949759E-01 -.99763349422631701635E-02

.57342204648407760562E-02 -.20113788382267643387E-02

.10824813836863072733E-02 .18650469800580292846E-02

.47400432061815163387E-01 -.16499332179959545763E-01

-.30767656167000584366E-01 -.16140446332964542009E+00

.47160473550337744797E+00 -.73044484775779050523E+00

-.14525934222839598231E+00 .89562845403103352737E+00

.37635010783137778922E+00 .13750965742335545874E-01

-.75870130673637344726E-02 .30260981361476765351E-02

.14623590461955504605E+00 .87935242169613658803E+00

.17069137681329938500E-01 -.33630938272844142223E-01

.28688770054978808555E-01 -.11752520319793203010E-01

.52167180793033290573E-02 -.30666369182306496902E-02

.13459784686851532492E-02 -.66993914093544163196E-03

.23635609149628820534E-03

-.58628167582634477251E-01 -.51337058742377683629E+00

.97857260969203274659E+00 .26080250008761735048E+00

-.12095261244288807445E+00 .43758719974053603141E-01

-.19116144607244232173E-01 .11247789499036625616E-01

-.49619416751048280381E-02 .24806062675043595925E-02

-.87755408375124086765E-03

.26968708133012803119E-01 .27077596845997020658E+00

-.46299650553520377283E+00 -.68269223536599976665E+00

.10462687420626342050E+01 .56533537255184640458E+00

.17369958188055874271E-01 -.10540998594085681031E-01

.51900245846781866213E-02 -.27417726409166252866E-02

.99552444740653436503E-03

-.12363141424821149847E-01 -.12238678744431417955E+00

.15585797989542082842E+00 .49635663179721822846E+00

-.75013263075120184542E+00 -.23718135148930338807E+00

.82484350907004400089E+00 .35259568412174530083E+00

-.16077674373257582526E-02 .20476469077578356680E-02

-.70451112177313324810E-03

.36307452109894428061E+00 .67563950971368391674E+00

-.33517521083176455947E-02 .25222093647833667904E-02

.22847340803181870675E-03 -.61712924522475608950E-03

.29035337550449889050E-03

-.19613970092261309719E+00 -.37175403833237968820E+00

.70531532139630537248E+00 .48911560141437010474E+00

-.58692366168934797729E-02 .36178271147195980792E-02

-.93000130952159538143E-03

.75054232777409080057E-01 .14958568044342079872E+00

-.35372655127015822929E+00 -.17050377006876304975E+00

.54703423473065826421E+00 .52329484286755134637E+00

.11348044605714474597E+00

.23962989083113392375E+00 .61602319205786482126E+00

.26362795861880333748E+00

0/

END

FIT

10S 4.71

11S 3.63

10P 5.00

11P 4.19

11P 3.22

10D 5.22

END

**Gold (TZP, 4f frozen)**

BASIS

1S 57.000

2S 36.650

3S 21.050

4S 12.400

2P 39.650

3P 17.950

4P 12.550

3D 22.100

4D 11.200

4F 8.000

1S 184.000

5S 7.000

5S 4.600

6S 3.150

6S 1.950

6S 1.210

5P 6.650

5P 4.500

5P 3.100

5D 5.050

5D 2.800

5D 1.500

6P 1.950

END

CORE 4 3 2 1

1S 760.000

1S 184.000

1S 87.700

2S 99.400

2S 38.300

3S 48.700

3S 20.750

4S 12.700

4S 9.000

5S 7.000

5S 4.600

6S 3.150

6S 1.950

6S 1.210

2P 65.900

2P 36.600

3P 20.800

3P 15.300

4P 13.550

4P 9.250

5P 6.000

5P 3.700

3D 28.000

3D 17.900

4D 12.200

4D 7.800

5D 5.050

5D 2.800

5D 1.500

4F 16.150

4F 8.900

4F 4.900

END

DESCRIPTION

.22018617895738584744E-01 .14116974977423085713E+00

.87207693657938767373E+00 .77680889589463730793E-02

-.25221315127642383869E-01 .22058018545083320910E-01

.85474922905381706291E-03 -.61155453930447493168E-03

.56849729991794632853E-03 -.30032240792580685088E-03

.13360337039928301024E-03 -.53805316319825778441E-04

.25808355788516243944E-04 -.89712035329347714497E-05

-.74301761942448460441E-02 -.55584553577420862946E-01

-.27607557570960511439E+00 -.85069418488694703573E-01

.98473277922767876724E+00 .10980611439562316944E+00

.36350035199224214541E-02 .88635186836117730094E-03

-.17929041216155235366E-02 .13928777214444929521E-02

-.75563740692087598503E-03 .33864950344558931083E-03

-.16981692269205694358E-03 .60095244924641582674E-04

.36155958996457898159E-02 .16135758013044225467E-01

.15781806613780821613E+00 -.79192180749857350730E-02

-.30880828962613571242E+00 -.37744400432206853813E+00

.11929913799355595927E+01 -.62861852690925560869E-02

.72405099200356084696E-02 -.26164496421376653816E-02

.68207106909025892738E-03 -.14251503497624676601E-03

.39225324072805088291E-04 -.94110898754734729913E-05

-.19006718691067976418E-02 -.27927537528881786998E-02

-.95151375339088767324E-01 .29786589973954117477E-01

.63260544421577677432E-01 .31623676965735764144E+00

-.87849784290291010880E+00 .10559412773604577307E+01

.21914770570424652041E+00 -.11095858047777526836E-01

.50126428323178711874E-02 -.25447872183713324788E-02

.13760467616083140851E-02 -.50472702572226942844E-03

.15128143207712455021E+00 .87214239235873480816E+00

.13229175940767643610E-01 -.20960022992712542406E-01

.16133812185068818756E-01 -.66520657387665266605E-02

.19863381480148575679E-02 -.70243588713429165171E-03

-.57814562752468760809E-01 -.51067204765293661151E+00

.97530755301405136493E+00 .22728829123725974304E+00

-.67054229068071236064E-01 .24485888776444180576E-01

-.72465894505855943386E-02 .25799466191666411974E-02

.25287909708401334896E-01 .26589730458413995962E+00

-.50687020012771810684E+00 -.47721541536086825586E+00

.93701274548289703592E+00 .52005190668748813021E+00

.90339002800095321438E-02 -.29097001873307293610E-02

.37550105477285616873E+00 .66530197545234670553E+00

-.48982083504430732473E-02 .39286895797436757749E-02

-.10497093965855495718E-02 .36613946156453309371E-03

-.11370041091496406861E-03

-.19911235427959006472E+00 -.34333924066275745002E+00

.72717859834297116084E+00 .45904078411905252510E+00

-.33164479837385543032E-02 .23456149893496436483E-02

-.77427476107391789758E-03

.28964845446318310263E+00 .62715619383714515944E+00

.23076743973531274601E+00

0/

END

FIT

9S 6.94

10S 4.43

11S 3.42

8P 8.56

10P 4.52

10P 3.45

8D 8.98

9D 6.96

9D 4.87

9F 4.00

END

**Boron (TZP, 1s frozen)**

BASIS

1S 4.42

2S 1.04

2S 1.70

2S 2.80

2P 0.68

2P 1.26

2P 2.60

3D 2.0

END

CORE 1 0 0 0

1S 6.50

1S 4.08

2S 1.04

2S 1.70

2S 2.80

END

DESCRIPTION

.23535242399080710807E+00 .77723858082216623622E+00

.12786517332543910225E-02 -.29315027241077996553E-02

.37407557753748525535E-02

0/

END

FIT

3S 1.36

2P 4.29

3P 2.55

3P 1.68

3D 3.33

3D 2.13

3D 1.36

END

**Barium (TZP, 4d frozen)**

BASIS

1S 36.800

2S 24.200

3S 11.300

4S 8.000

2P 25.750

3P 12.450

4P 7.300

3D 14.350

4D 5.400

1S 106.300

5S 4.000

5S 2.550

6S 1.750

6S 1.150

6S .780

5P 3.700

5P 2.500

5P 1.750

6P 1.150

5D 2.200

5D 1.100

4F 2.000

END

CORE 4 3 2 0

1S 427.000

1S 106.300

1S 57.500

2S 64.900

2S 24.800

3S 13.200

3S 10.000

4S 9.750

4S 6.200

5S 4.000

5S 2.550

6S 1.750

6S 1.150

6S .780

2P 38.100

2P 23.900

3P 14.000

3P 10.650

4P 7.800

4P 5.300

5P 3.700

5P 2.500

5P 1.750

3D 18.050

3D 10.850

4D 7.750

4D 5.000

4D 3.450

END

DESCRIPTION

.11373609102224768383E-01 .86833536086007245824E-01

.93007151379566288529E+00 -.11585462054878125845E-01

.12163506853406311908E-02 -.41531013700343748576E-02

.68532806640778246368E-02 -.38992749915521219758E-02

.68297583751768416432E-03 -.25677783414214085589E-03

.15691719324424481335E-03 -.89470419233557410750E-04

.56338090287532646737E-04 -.21131105415008863202E-04

-.33411877016894029994E-02 -.75171847217431211896E-01

-.19625961083264162332E+00 -.18402335794592131202E+00

.11174615195336645712E+01 .21193140004194827714E-01

-.38115215637354593614E-01 .24648675562397099476E-01

-.56525258010274919904E-02 .24341550156313663394E-02

-.15573033676764204587E-02 .91026995404054611687E-03

-.57900960438139336024E-03 .21818864070725086366E-03

.11185103051405288072E-02 .68370890420563754097E-01

.71906926234318480767E-02 .16834112480618240726E+00

-.68076900467442180709E+00 .79570762777675474453E+00

.66473213816147069810E+00 -.37742747944513260761E+00

.82293212602230991770E-01 -.34647072435994935580E-01

.22141436290996716757E-01 -.12986434303412393992E-01

.82853102666067147269E-02 -.31284389490432852436E-02

-.50414456378712049420E-03 -.35399050121749417452E-01

.33642952762967569605E-02 -.87620204756397770951E-01

.34157383457518314396E+00 .14164671481873470152E-01

-.16306206325263494783E+01 .15000391221475448678E+01

.62690240755211878998E+00 .19458250856122918937E-01

-.12090794996109927376E-01 .77489456888076083119E-02

-.51715547583642646964E-02 .19990843513713451446E-02

.21329134889297321487E+00 .80649273519242803410E+00

.10576517252775970851E-01 -.12582106489151020923E-01

.91115023457743297974E-02 -.64100352686617496564E-02

.35518966220105929049E-02 -.23471530328490705121E-02

.84374222763821001263E-03

-.73689129508912515343E-01 -.46268285724196661635E+00

.76369453247733809498E+00 .39989760586134726061E+00

-.18466818870571777289E-01 .12214948161469407067E-01

-.67238338775726025007E-02 .44883085463203447468E-02

-.16289619589640218569E-02

.29579823518368415780E-01 .22795089599409879000E+00

-.40386100101829702780E+00 -.34139023622480729747E+00

.82594961856337745232E+00 .42955187863636212775E+00

.97468943636819429382E-02 -.68764663269392234390E-02

.28908967625974838628E-02

.40898284247634697497E+00 .64711218264684600143E+00

-.11052981595215432414E-01 .79216473383099499095E-02

-.20969051938893087245E-02

-.18733707057833812182E+00 -.29962691110332728694E+00

.52026177590954569840E+00 .56878066161845819426E+00

.92623829555676509551E-01

0/

END

FIT

9S 5.41

10S 4.11

10S 3.43

11S 2.21

11S 1.86

8P 5.57

9P 4.66

10P 2.25

10D 1.88

8F 4.43

9F 1.88

9G 3.87

9G 2.20

END

**Beryllium (TZP, 1s frozen)**

BASIS

1S 3.44

2S 0.78

2S 1.22

2S 2.22

2P 0.80

2P 1.50

3D 1.90

END

CORE 1 0 0 0

1S 5.4600

1S 3.1800

2S 0.7800

2S 1.2200

2S 2.2200

END

DESCRIPTION

.20765834995432722043E+00 .81146803211735540895E+00

-.20642593701102680999E-02 .52632219986129338829E-02

-.53150282176997959774E-02

0/

END

FIT

3S 1.48

2P 3.17

2P 1.78

3P 1.48

3D 1.48

END

**Bismuth (TZP, 5d frozen)**

BASIS

1S 61.000

2S 38.700

3S 22.600

4S 13.250

5S 7.800

2P 40.250

3P 20.300

4P 12.850

5P 7.000

3D 23.700

4D 11.900

5D 4.100

4F 9.250

1S 201.000

6S 3.850

6S 2.550

6S 1.720

6P 2.900

6P 1.750

6P 1.070

END

CORE 5 4 3 1

1S 835.000

1S 201.000

1S 93.800

2S 108.200

2S 40.900

3S 51.950

3S 22.250

4S 13.600

4S 9.700

5S 7.900

5S 5.400

6S 3.850

6S 2.550

6S 1.720

2P 71.600

2P 38.900

3P 22.050

3P 16.050

4P 14.800

4P 10.150

5P 6.800

5P 4.400

6P 2.900

6P 1.750

6P 1.070

3D 29.850

3D 19.150

4D 13.250

4D 8.650

5D 6.150

5D 3.850

5D 2.400

4F 17.950

4F 10.300

4F 6.050

END

DESCRIPTION

.24546174743581086058E-01 .15308751685267227294E+00

.85878546549040890579E+00 .13086250872962520639E-01

-.33407976545256817813E-01 .29335354984877198142E-01

.96842783954249759062E-03 -.57736896962581093617E-03

.53330712434114719601E-03 -.28419987059580568631E-03

.11728264307100355851E-03 -.45659532963269062458E-04

.22595605341112454551E-04 -.74518987178869502968E-05

-.83308858937783183962E-02 -.60479870912815682227E-01

-.27056424331917561243E+00 -.83275772442121726025E-01

.99787048811670464588E+00 .94557698235203505033E-01

.18128855988242254232E-02 .38555512300010594609E-02

-.57850573052990938647E-02 .44386309663663721728E-02

-.23907019397378347081E-02 .11212636030842049184E-02

-.60824482012821325192E-03 .20974403489884709375E-03

.40543339587088920301E-02 .19132491465847788897E-01

.15481736934159029406E+00 -.51694610517937821317E-02

-.33619479267651636301E+00 -.35386967226246457408E+00

.11956789695304501642E+01 -.61368948601896915543E-02

.69230656977716182113E-02 -.20047950786149589149E-02

-.49715211432555621025E-04 .34522997895953248153E-03

-.26559053197067401027E-03 .10416416780589884299E-03

-.21440571214755383897E-02 -.46097471888331010420E-02

-.94214549514692172427E-01 .27101637330308035734E-01

.85686139977104933130E-01 .30118104680250812333E+00

-.89133010320198802212E+00 .10839169350632764655E+01

.19666577993315079165E+00 -.17203115246439089348E-01

.99337224383858856169E-02 -.58109340537104399796E-02

.35214829524558739771E-02 -.12839231558721671354E-02

.10212642152696597628E-02 .12095895288401230152E-02

.47085567249872291307E-01 -.17257148771298913559E-01

-.24222652609771147536E-01 -.16245958855965692003E+00

.46032267024905554331E+00 -.71696885751814998233E+00

-.10558688451864779723E+00 .87361329389775310705E+00

.36146476421917139721E+00 .10803707794729066477E-01

-.58181947380696787814E-02 .23882661096483368511E-02

.14767164654585032690E+00 .87684927978171356422E+00

.17100900315482066294E-01 -.30895572225587180498E-01

.25428137431597271773E-01 -.10448958109872339903E-01

.40982353862494590516E-02 -.22008003249748904658E-02

.93310933091138173814E-03 -.47713288314555637147E-03

.17562772538167449013E-03

-.58256744344736162422E-01 -.51250297589841653512E+00

.98235080107575001307E+00 .24209245516717056712E+00

-.10028031569381283539E+00 .37097078690484501517E-01

-.14479303264289542300E-01 .78167789648849114736E-02

-.33384322749502519205E-02 .17150179796994536111E-02

-.63284206334014183031E-03

.26314197067388810786E-01 .26932942771090051526E+00

-.48816901615680996507E+00 -.59246367172402292223E+00

.10001359815830110911E+01 .54701115098978103646E+00

.15637256676381959541E-01 -.82995204299393336017E-02

.38762413337020090558E-02 -.20786530216434047011E-02

.78196213197607512043E-03

-.11752032192708072519E-01 -.11827196613983290141E+00

.16717710348466688353E+00 .42623920049963870404E+00

-.70392087406367398472E+00 -.20072721811966279248E+00

.82258151748289543015E+00 .34593884045233069813E+00

-.56128609253200392390E-02 .37867965333116342629E-02

-.13933818880534287136E-02

.36788405936502882598E+00 .67203967631389793791E+00

-.49583782245003074976E-02 .38104686259953272041E-02

-.77187402643014017042E-03 .66811727184624698350E-04

.29106192113685300238E-04

-.19717127345019797025E+00 -.36351737899545755184E+00

.71205905815997172503E+00 .48226591278532776608E+00

-.74500718433327646831E-02 .43751354836442882951E-02

-.12787447271842109116E-02

.70223671996906877890E-01 .13746531918051735355E+00

-.33538305828540621034E+00 -.13960254838656166454E+00

.54531871854318270110E+00 .51540855907429428129E+00

.12093493531540314145E+00

.25764411364644218549E+00 .61613734758143634540E+00

.25327945081844965847E+00

0/

END

FIT

10S 5.83

11S 2.56

10P 5.80

11P 2.79

10D 4.93

10F 3.47

END

**Bromine (TZP, 3d frozen)**

BASIS

1S 22.50

2S 14.30

3S 6.75

2P 16.15

3P 6.65

3D 5.34

4S 1.80

4S 2.80

4S 4.25

4P 1.20

4P 1.95

4P 3.20

4D 1.8

END

CORE 3 2 1 0

1S 32.8000

1S 41.3000

2S 13.9000

3S 4.9500

3S 7.2000

4S 2.8000

4S 1.8000

4S 4.5000

2P 13.1000

2P 19.4500

3P 5.0500

3P 7.9000

4P 1.2000

4P 1.9500

4P 3.2500

3D 3.5000

3D 6.0500

3D 10.9000

END

DESCRIPTION

.59605432813271552739E+00 .40379341487546988043E+00

.15352005925122496136E-01 .57395062565161629464E-01

-.37028567725496841501E-01 .11472274721394018493E-01

-.34797589341823264436E-02 -.37440830115832025859E-01

-.48542381771376863497E+00 .84193162900644213931E-01

.11189919956564216008E+01 .14196749542657433052E+00

-.10121487604610512889E+00 .24404440679338470360E-01

-.71643540525313069434E-02 -.86638221698010345340E-01

.25640492889948202082E+00 -.78065889432532217795E-01

-.58785163485596725330E+00 .14479998196567639424E+00

.99166648996338135902E+00 -.39690719446847950308E-02

.42562307080422759396E-04 .30593016174723611234E-01

.62427291019852659826E+00 .39313623089516303022E+00

-.12877715032817323160E-01 .16125392476858197571E-01

.16413101717873972325E-02 -.43720510524642049177E-02

.69089204822407298059E-02

-.31931714836664953916E+00 -.13719474523593180870E+00

.48768664076964546306E+00 .66231971042035042707E+00

-.14896496348283446310E-02 .41865195895933546982E-02

-.57424511573696320615E-02

.27765153474675258183E+00 .58011906448084959997E+00

.25810992141226096752E+00

0/

END

FIT

6S 5.92000000000000

7S 2.88000000000000

6P 3.89000000000000

7P 3.86000000000000

7P 2.78000000000000

7D 5.12000000000000

7F 3.37000000000000

END

**Carbon (TZP, 1s frozen)**

BASIS

1S 5.40

2S 1.28

2S 2.10

2S 4.60

2P 0.82

2P 1.48

2P 2.94

3D 2.2

END

CORE 1 0 0 0

1S 7.68

1S 5.00

2S 1.28

2S 2.10

2S 4.60

END

DESCRIPTION

.24480746892337518217E+00 .76163894174335367016E+00

.17339063337058347857E-02 -.33959965802830903263E-02

.87269724034686312758E-02

0/

END

FIT

2S 5.52999999999998

2S 4.98000000000000

3S 4.79000000000000

3S 2.34000000000000

3P 3.09000000000000

3P 4.20000000000000

3P 1.96000000000000

3D 7.18000000000000

3D 4.44000000000000

3D 2.60000000000000

3D 1.64000000000000

4F 3.55000000000000

END

**Calcium (TZP, 3p frozen)**

BASIS

1S 13.25

2S 7.75

3S 3.25

2P 7.32

3P 2.68

4S 0.65

4S 1.05

4S 1.75

4P 1.06

3D 1.50

3D 3.50

END

CORE 3 2 0 0

1S 24.4000

1S 18.2500

2S 7.4000

2S 4.8500

3S 4.0000

3S 2.5500

4S 0.7000

4S 1.0500

4S 1.6500

2P 10.8500

2P 6.4500

3P 1.8500

3P 2.7000

3P 4.0000

END

DESCRIPTION

.25978903709524903132E+00 .74365971124386254232E+00

.19801809519918053132E-01 -.26605534704150949482E-01

.18444137019035720254E-01 -.89171176145810583658E-02

.99035096060814107634E-03 -.26873266289614431215E-02

.40566496729237287375E-02

.64426021816616413362E-01 -.42791094672971730084E+00

.10247938352085514957E+01 .71896700986887426832E-01

-.20267966657095008720E-01 .12637023733740947173E-01

-.19883421378967011499E-02 .51898500187391605892E-02

-.72138407383330587636E-02

-.33047234488223907467E-01 .16913170211300632140E+00

-.33161678698535201892E+00 -.29727091449164222414E+00

.80742498635528170414E+00 .46991069599694329506E+00

.40376301539758177248E-02 -.95870865038835857697E-02

.12911353486446409608E-01

.35199926078806187402E+00 .68352910944844436081E+00

.14703805309505895652E-02 -.22338226179057377521E-02

.25619218592213529109E-02

-.11048268309754295802E+00 -.27399607758340288610E+00

.19497340587590056282E+00 .56378590532672545610E+00

.37467333817227516102E+00

0/

END

FIT

6S 6.54

7S 1.99

6P 2.12

END

**Cadmium (TZP, 4p frozen)**

BASIS

1S 31.900

2S 20.800

3S 9.600

4S 6.200

2P 21.700

3P 10.300

4P 4.650

3D 11.850

5S 2.800

5S 1.700

5S 1.070

4D 5.100

4D 2.900

4D 1.630

5P 1.700

END

CORE 4 3 1 0

1S 333.000

1S 82.900

1S 48.200

2S 56.050

2S 20.600

3S 10.700

3S 6.650

4S 6.800

4S 4.300

5S 2.800

5S 1.700

5S 1.070

2P 30.700

2P 19.750

3P 11.750

3P 8.600

4P 6.450

4P 4.350

4P 3.050

3D 18.600

3D 11.200

3D 7.500

4D 5.100

4D 2.900

4D 1.630

END

DESCRIPTION

.90481233134411479663E-02 .76818636748975085982E-01

.94413758436287875675E+00 -.18504101254378790831E-01

.91494826423400377278E-03 -.16670998156085845877E-02

.36585216505409562555E-02 -.28860643604238030331E-02

.52716048824634823260E-03 -.19134541444621057773E-03

.10218776863443945139E-03 -.37484587735200557374E-04

-.24123977257548205426E-02 -.12898751136305014464E+00

-.79987901843304565208E-01 -.24851445062120525176E+00

.11138975583007246772E+01 .28273605673607986495E-02

-.59877935967491040595E-02 .58963350469622092392E-02

-.17573623468122008626E-02 .77781915320372771363E-03

-.44630784741086349356E-03 .16846835789641426031E-03

.60919562089119065878E-03 .12312382350617330495E+00

-.10905573266022178247E+00 .22746526529997948551E+00

-.65663749944504734568E+00 .10578584227894973768E+01

.23120944597593015568E+00 -.15958836558822322993E+00

.34655254521458192518E-01 -.14783545262024459788E-01

.84485195675477707533E-02 -.31950736113254205040E-02

-.21818589640104464546E-03 -.62285351318513045016E-01

.65061438045721939338E-01 -.11442349016191645517E+00

.30870832667243980341E+00 -.48339385688968911081E+00

-.69382649265894824619E+00 .12715348086455326992E+01

.44760476886767475158E+00 .12969424565704448793E-01

-.69763269507781477605E-02 .28995004257184654219E-02

.24881668521209854283E+00 .77160907266404121785E+00

.67229538441496471471E-02 -.83516110134674876458E-02

.65535106454585705105E-02 -.46924282972349113871E-02

.17210781376221866677E-02

-.85190323792826053162E-01 -.43061032685479910809E+00

.69338531727110463976E+00 .46341471569966408151E+00

-.84392048467020336355E-02 .55507928413017970301E-02

-.19191483275005946467E-02

.32550851214812839829E-01 .18930874901947122857E+00

-.31786383863600209398E+00 -.33750273511373185542E+00

.59337996106420376474E+00 .53326781864335925931E+00

.12023505605131949714E+00

.14643538401091299450E+00 .58595287449915001154E+00

.33324023745062736346E+00 -.17702449823566209896E-02

.98231122150281070284E-03 -.29278900617828331312E-03

0/

END

FIT

9S 3.96

9S 2.63

8P 4.70

9P 2.77

7D 5.32

8D 2.70

END

**Chlorine (TZP, 2p frozen)**

BASIS

1S 13.95

2S 5.65

2P 6.70

3S 1.60

3S 2.30

3S 3.30

3P 1.20

3P 2.05

3P 2.85

3D 2.00

END

CORE 2 1 0 0

1S 20.30

1S 15.25

2S 6.25

2S 4.45

3S 1.60

3S 2.30

3S 3.45

2P 8.85

2P 5.05

3P 1.20

3P 2.05

3P 3.35

END

DESCRIPTION

.29450852085577067241E+00 .70931233652257552613E+00

.22471401804959500320E-01 -.27897346182188625247E-01

.36905682931946026844E-02 -.10935371214404175991E-01

.17343862900148287326E-01

.52122198949804257606E-01 -.40213904312159348686E+00

.90517699258131267825E+00 .18967740230286964098E+00

-.60779552524764237850E-02 .15341351375187160452E-01

-.19708648992745079337E-01

.38449875087383306171E+00 .65794776373117913604E+00

.94667543824818634231E-03 -.12565839797322396997E-02

.28500210347375187422E-02

0/

END

FIT

4S 4.75000000000000

5S 2.77000000000000

5S 6.28000000000000

5S 1.89000000000000

4P 4.28000000000000

5P 5.78000000000000

5P 3.04000000000000

5D 3.87000000000000

5D 2.59000000000000

4F 5.41000000000000

4F 2.39000000000000

END

**Cobalt (TZP, 3p frozen)**

BASIS

1S 14.10

2S 11.65

3S 4.85

2P 10.74

3P 4.18

3D 1.50

3D 3.25

3D 6.70

4S 0.85

4S 1.40

4S 2.40

4P 1.43

END

CORE 3 2 0 0

1S 32.2500

1S 25.0000

2S 10.5000

2S 6.9500

3S 6.0000

3S 3.7500

4S 0.9000

4S 1.4000

4S 2.3000

2P 15.2000

2P 9.6500

3P 5.4500

3P 3.2500

END

DESCRIPTION

.32393627069371688787E+00 .67555266434687732957E+00

.45686269615884476936E-01 -.62695453780890322837E-01

.39873729264645184356E-01 -.15057233786809514839E-01

.14328164869961681691E-02 -.37568589528077481372E-02

.58291920823719421338E-02

.89052698922833295470E-01 -.47823741187855273171E+00

.10561139786233206816E+01 .63199991699697574687E-01

-.43131581580227625061E-01 .22419467244612387852E-01

-.27610296740680022841E-02 .70561148065792734727E-02

-.10334764377718184611E-01

-.50327620343073219134E-01 .20618391406534866683E+00

-.34249850309607487997E+00 -.39667593825398883567E+00

.93528503151189346099E+00 .42884463006556422515E+00

.47263417983190152832E-02 -.10958112139420623371E-01

.13655259649339690844E-01

.33653779541509609707E+00 .69008898074940216993E+00

.22046048910434419207E-02 -.57672067162025080888E-03

-.11589804958542981872E+00 -.30130066083108503205E+00

.71182962484452472207E+00 .42860506422585586961E+00

0/

END

FIT

6S 7.14

7S 3.15

7S 2.32

5P 9.08

6P 6.16

6P 3.60

7P 2.48

5D 11.14

7D 2.86

6F 2.93

END

**Chromium (TZP, 3p frozen)**

BASIS

1S 13.85

2S 10.00

3S 4.15

2P 9.28

3P 3.58

3D 1.24

3D 2.70

3D 5.70

4S 0.80

4S 1.30

4S 2.20

4P 1.30

END

CORE 3 2 0 0

1S 27.2500

1S 21.7000

2S 9.2000

2S 6.0500

3S 5.2500

3S 3.3000

4S 0.8400

4S 1.2800

4S 2.0500

2P 13.2000

2P 8.2500

3P 4.6500

3P 2.8000

END

DESCRIPTION

.43427910634164273995E+00 .56442503309148872592E+00

.47408786255677834443E-01 -.65503958463408992752E-01

.42672576432440927074E-01 -.16477695568343539434E-01

.16871968230534599865E-02 -.44848913031334527654E-02

.67326928147217685386E-02

.85648033597503656078E-01 -.47041553149860843019E+00

.10523754686740456332E+01 .59794390999302261813E-01

-.27010717160597344511E-01 .14622111489884364113E-01

-.20613026374999314158E-02 .53043870010220112859E-02

-.74187629080587928418E-02

-.49427335468510351368E-01 .20121328658237822018E+00

-.33179364730974064690E+00 -.39532686316149001637E+00

.88166119393047415187E+00 .47626964336068372541E+00

.34205039304706697824E-02 -.79126042983085221466E-02

.10123176334486075345E-01

.35382814158660724768E+00 .67521048181887077799E+00

.28258255945464959280E-02 -.10167562206283354328E-02

-.12129592003976694126E+00 -.28592141743672799814E+00

.69509954037669574589E+00 .43933415819678278691E+00

0/

END

FIT

5S 10.08

6S 4.55

7S 2.89

6P 5.45

6P 3.26

7P 2.30

6D 6.30

5G 5.17

END

**Cesium (TZP, 4d frozen)**

BASIS

1S 36.000

2S 26.300

3S 11.200

4S 7.950

2P 25.100

3P 12.300

4P 7.000

3D 14.000

4D 5.150

1S 102.900

5S 3.750

5S 2.350

6S 1.750

6S 1.050

6S .660

5P 3.550

5P 2.300

5P 1.500

6P 1.050

5D 2.000

5D 1.000

4F 2.000

END

CORE 4 3 2 0

1S 413.000

1S 102.900

1S 56.300

2S 63.800

2S 24.250

3S 12.900

3S 9.500

4S 9.350

4S 5.950

5S 3.750

5S 2.350

6S 1.750

6S 1.050

6S .660

2P 37.100

2P 23.350

3P 13.750

3P 10.450

4P 7.500

4P 5.050

5P 3.550

5P 2.300

5P 1.500

3D 17.600

3D 10.550

4D 7.500

4D 4.800

4D 3.250

END

DESCRIPTION

.11127187032081421042E-01 .86106598973145806464E-01

.93087938656639745982E+00 -.12073096444304464922E-01

.11750182935410047672E-02 -.36427142426264476002E-02

.63038338175669368993E-02 -.38250293375274645007E-02

.65221301471782636150E-03 -.24122839419132526733E-03

.16307897536853695414E-03 -.88303667996382612343E-04

.38339084657433014224E-04 -.14157136376149895372E-04

-.32421036603922015126E-02 -.79924043736560326789E-01

-.18563006778831681043E+00 -.18988305061563165443E+00

.11177426351815455963E+01 .11349683652858754204E-01

-.22835139196125793515E-01 .16615889866213824633E-01

-.40706244747535482234E-02 .17841235070859228200E-02

-.12717338009374014058E-02 .70565090191401845347E-03

-.31078332020725727087E-03 .11536258812355857251E-03

.10557417610878201686E-02 .73978641488832069806E-01

-.50463128618899169217E-02 .17490787422958301023E+00

-.67942297219067382397E+00 .85250543055352545618E+00

.58860179329798545389E+00 -.34956093427347528779E+00

.73410035984461732839E-01 -.30526191749853465174E-01

.21574061570205051858E-01 -.11967157230448196084E-01

.52797096221097504845E-02 -.19624338974866270893E-02

-.46736846922596988899E-03 -.38243030857247013732E-01

.10120691791476394208E-01 -.90743318650834764449E-01

.33858947765439367794E+00 -.96470428981311112659E-01

-.14856149778148650675E+01 .14927276821831974019E+01

.60434370734773257272E+00 .17797850489895471887E-01

-.12149796643959646925E-01 .72516693845611317204E-02

-.33784440819808678191E-02 .12852191561388880484E-02

.21843119468705898822E+00 .80211986014300251657E+00

.69400188617227808602E-02 -.85728635679950416365E-02

.65690513088646762530E-02 -.47368783586362161325E-02

.24428505250679187141E-02 -.13823492770086813486E-02

.47195467458742862985E-03

-.75334573843105323565E-01 -.45946454005486359051E+00

.74456901834867394996E+00 .41712580630348206068E+00

-.13975952764458531019E-01 .95384551338334212789E-02

-.48413318653855721627E-02 .27563468294204240654E-02

-.94870358593830553858E-03

.29782493600765208575E-01 .22454557624997004139E+00

-.39744887984835014505E+00 -.32657373992129173912E+00

.82971458658866648062E+00 .41179528578817203854E+00

.74557315955440682173E-02 -.45153933591247476589E-02

.18605996163681473837E-02

.41306139403380942587E+00 .64258942411518704674E+00

-.85909873067816274611E-02 .66455081892923114836E-02

-.16540546095417999178E-02

-.18559030477649610114E+00 -.28860931635034492437E+00

.51065092797782829503E+00 .56688834208442295814E+00

.10338084564106898855E+00

0/

END

FIT

9S 4.74

10S 2.96

10S 2.46

9P 4.30

9P 3.22

10P 2.05

10D 2.34

END

**Copper (TZP, 3p frozen)**

BASIS

1S 14.30

2S 12.80

3S 5.30

2P 11.71

3P 4.53

3D 1.28

3D 3.10

3D 6.90

4S 0.85

4S 1.40

4S 2.45

4P 1.37

END

CORE 3 2 0 0

1S 34.80

1S 27.00

2S 11.35

2S 6.60

3S 6.70

3S 4.05

4S 0.86

4S 1.40

4S 2.40

2P 16.05

2P 10.45

3P 6.00

3P 3.50

END

DESCRIPTION

.32719495826575639708E+00 .67160552365945680453E+00

.50130506692236058430E-01 -.93712530478825287195E-01

.69994820503186144789E-01 -.14651884502090619516E-01

.11936617251035593849E-02 -.31034577342938175944E-02

.50925973127004938931E-02

.90169200906398488948E-01 -.48342182793878019087E+00

.10792879550353280393E+01 .48373742422133825247E-01

-.48312078627883649207E-01 .18301479218742228161E-01

-.20130410123787383612E-02 .51011860052754638348E-02

-.78608815077525207027E-02

-.51063291626816928459E-01 .20885176247430531360E+00

-.33810725014468478999E+00 -.59351979277011313041E+00

.11277254236229756756E+01 .45102256282693564682E+00

.23285536767258664496E-02 -.51763929050106013280E-02

.64483880376193184608E-02

.37066114226633273265E+00 .65363014570697741679E+00

.40532148053367177479E-02 -.17765113873764698093E-02

-.12788509846331549968E+00 -.29232746492066019606E+00

.71380058401358237319E+00 .43330707741682694678E+00

0/

END

FIT

7S 3.20

5P 9.24

6P 3.51

7P 2.37

6D 7.43

6D 4.15

7D 2.74

END

**Fluorine (TZP, 1s frozen)**

BASIS

1S 8.33

2S 8.00

2S 0.74

2S 1.94

2S 3.24

2P 1.24

2P 2.30

2P 4.54

3D 2.00

END

CORE 1 0 0 0

1S 10.8800

1S 7.7000

2S 1.9400

2S 3.2400

2S 0.7400

END

DESCRIPTION

.28978578929459486435E+00 .71851567156830775662E+00

-.22668143369522085646E-02 .31161712531234580029E-02

.53090331019027352764E-03

0/

END

FIT

2S 8.17000000000000

3S 3.64000000000000

3S 2.76000000000000

3P 3.34000000000000

3P 2.07000000000000

3D 6.00000000000000

3D 2.40000000000000

4F 3.11000000000000

END

**Iron (TZP, 3p frozen)**

BASIS

1S 14.35

2S 11.05

3S 4.60

2P 10.25

3P 3.98

3D 1.40

3D 3.05

3D 6.40

4S 0.85

4S 1.40

4S 2.40

4P 1.39

END

CORE 3 2 0 0

1S 30.45

1S 23.90

2S 10.05

2S 6.35

3S 6.10

3S 3.70

4S 0.90

4S 1.40

4S 2.30

2P 14.60

2P 9.20

3P 5.20

3P 3.10

END

DESCRIPTION

.36192266398710098985E+00 .63680979384668456866E+00

.54195890622947419379E-01 -.88785570960143514463E-01

.60652511122246573205E-01 -.16104884376681109781E-01

.15300041328752331249E-02 -.40365325463932984554E-02

.63741602926594490008E-02

.89768049727271162497E-01 -.47737781637671344859E+00

.10555513067973438801E+01 .73992006531672663283E-01

-.52572385663977851122E-01 .20849605803622772715E-01

-.26246173487387996527E-02 .67319975631636658556E-02

-.99741187017448780516E-02

-.48109916026499180419E-01 .20159932694680987586E+00

-.27959535098328713865E+00 -.60841808605157388090E+00

.10027501752331309248E+01 .53114837154178762280E+00

.16671250680436889430E-02 -.35127016362889755194E-02

.48796683498209076843E-02

.33569888749914128523E+00 .69183726361712050146E+00

.19856413851412060487E-02 -.43582994920330134486E-03

-.11457284824804236933E+00 -.30096384424055472318E+00

.70527849423995470080E+00 .43496661342823372820E+00

0/

END

FIT

7S 4.29

7S 1.70

6P 6.05

6P 3.54

7P 2.44

5D 10.91

6D 4.12

7D 2.78

END

**Francium (TZP, 5d frozen)**

BASIS

1S 65.000

2S 40.750

3S 24.050

4S 14.000

5S 8.850

2P 39.950

3P 23.550

4P 12.800

5P 8.250

3D 24.500

4D 13.200

5D 5.050

4F 10.450

1S 220.000

6S 4.200

6S 2.650

7S 1.850

7S 1.150

7S .720

6P 3.250

6P 1.900

7P 1.150

6D 2.300

6D 1.150

5F 2.000

END

CORE 5 4 3 1

1S 931.000

1S 220.000

1S 100.200

2S 118.900

2S 43.600

3S 55.450

3S 23.800

4S 14.550

4S 10.400

5S 8.950

5S 6.250

6S 4.200

6S 2.650

7S 1.150

7S 1.850

7S .720

2P 77.700

2P 41.250

3P 23.400

3P 17.000

4P 11.100

4P 16.200

5P 7.650

5P 5.150

6P 3.250

6P 1.900

3D 31.800

3D 20.450

4D 14.300

4D 9.550

5D 6.250

5D 3.800

4F 19.950

4F 11.750

4F 7.150

END

DESCRIPTION

.26844561446598625559E-01 .16949314004007259848E+00

.83608409980171505271E+00 .26911581255013323349E-01

-.52866131181967229713E-01 .46488509022064056220E-01

.14167754309154397246E-02 -.58464479158773475457E-03

.40741116305395363257E-03 -.11224798896590627666E-03

-.96267948550949011100E-05 .16025897317573208013E-04

-.11100321946836720359E-04 -.31361206721925851726E-05

.61514610346878299214E-05 .11563343000722385295E-05

-.91584418696322514308E-02 -.67511114121818302514E-01

-.25970666154060184638E+00 -.84416349914386859532E-01

.10115936762295911766E+01 .78009676832446528394E-01

-.15962614679971686565E-03 .75782248175469361234E-02

-.11556001456708209219E-01 .85794205402700135510E-02

-.36044519377996251178E-02 .12731822173465022138E-02

-.68511663671444824643E-03 -.17007094540453878318E-03

.34595790377222479127E-03 .61888938077069054807E-04

.44560704126072395170E-02 .22955257324776483230E-01

.14986345440671522411E+00 -.10375659201294687699E-02

-.36575940342970603147E+00 -.32617177326055374342E+00

.11970161057874719734E+01 -.57686798999890430070E-02

.58535274321929219379E-02 -.66315107359914734608E-03

-.10085484321595875826E-02 .67206100409337088050E-03

-.42742516310956062912E-03 -.11671656883302556241E-03

.23093001680733440039E-03 .42914472246083841024E-04

-.23705514217054793856E-02 -.68233681972509202529E-02

-.92523698043241328626E-01 .23450726781317172281E-01

.11143953499020779752E+00 .28167841403272564360E+00

-.90274218306549869340E+00 .11066358520593602144E+01

.18417108904703977101E+00 -.28321968603959357669E-01

.13625541714952142322E-01 -.61682311166514989381E-02

.36680793257040647179E-02 .97706204987477738091E-03

-.19437231387001889862E-02 -.35882190807802721421E-03

.11707062827545934840E-02 .23933336768639209072E-02

.48012226152220824349E-01 -.15723119823493249964E-01

-.39787568193109620895E-01 -.15757826695581686471E+00

.48500615349219200478E+00 -.74015903823590234456E+00

-.19412371472293704633E+00 .90265627218299604451E+00

.41389827019489755511E+00 .93379327358099790957E-02

-.45729205343714640283E-02 -.13445254347082742120E-02

.25752684335398458651E-02 .50263497249284413287E-03

.14448979514769483279E+00 .88129910148368195699E+00

.21143841603194900303E-01 -.41737521507408344157E-01

-.13129763943777844962E-01 .34646398560858132631E-01

.53868465321030983839E-02 -.26549469046194067688E-02

.80131141964427286210E-03 -.27813154032627903723E-03

-.58723124762538055077E-01 -.51405875752389185163E+00

.96112632861635238779E+00 .29864693842193873463E+00

.49283243049854583151E-01 -.14786156252371021602E+00

-.19847552329854623726E-01 .97778403828774566181E-02

-.29643678868996907114E-02 .10326353305657556044E-02

.27435799416053532201E-01 .27220293760643188286E+00

-.43245881622867210492E+00 -.77494610918461137583E+00

.58806879586507132540E+00 .10838601782059835710E+01

.18182866799620823334E-01 -.89885949827626272779E-02

.30372572056631638206E-02 -.11063591312075987238E-02

-.12820990685564711761E-01 -.12656248087530586055E+00

.14748877488668446500E+00 .55288201587918373026E+00

-.29269634530398930261E+00 -.77291650176856308008E+00

.83384177001252024475E+00 .36592152609710387745E+00

-.38282755562927366089E-02 .18230167617952093822E-02

.35684465217805733106E+00 .68167876068381150212E+00

-.39220097925931852664E-02 .31359689338357980143E-02

-.42056993115400283928E-03 .39652394033626818656E-04

-.19324984742330028609E+00 -.38400543732925995766E+00

.69781942448223466258E+00 .49926043711853240747E+00

-.36444328509162410810E-02 .19368539403362234827E-02

.75041101741297785988E-01 .17191370560630594988E+00

-.40692300425947586229E+00 -.11406862841258501307E+00

.78104820473553149274E+00 .34916502998725051921E+00

.22096110490911993751E+00 .61197114245518646758E+00

.28008643818640865941E+00

0/

END

FIT

11S 3.09

11S 2.55

10P 5.41

11P 4.56

11P 3.52

12P 2.97

12P 2.32

12D 2.71

10F 4.73

11G 2.50

END

**Gallium (TZP, 3d frozen)**

BASIS

1S 16.75

2S 13.40

3S 5.75

2P 13.80

3P 5.80

3D 3.60

4S 1.20

4S 1.90

4S 3.05

4P 0.70

4P 1.25

4P 2.25

4D 1.5

END

CORE 3 2 1 0

1S 28.8500

1S 36.5000

2S 12.2500

2S 7.0500

3S 4.6000

3S 7.6000

4S 1.2000

4S 1.9000

4S 3.1000

2P 11.3000

2P 17.1000

3P 3.9500

3P 6.6000

4P 0.7000

4P 1.2500

4P 2.3000

3D 2.1000

3D 4.2500

3D 8.4500

END

DESCRIPTION

.60541299506334134151E+00 .39001262263183822387E+00

.89299286174358921686E-01 -.19780660695391641402E+00

-.26188432900687709515E-01 .15335778135813113443E+00

.22271638670645953553E-02 -.63710377634384907780E-02

.11516404046766645294E-01

-.49008816624109574622E+00 .92811937851834475088E-01

.10668630655232438365E+01 .10358939591070538688E+00

.32809232829711841573E-01 -.10269936813588888269E+00

-.36084395355321062515E-02 .10006009499064960969E-01

-.16914035640934837329E-01

.21234297149646183533E+00 -.51937004750889734228E-01

-.26198807609205676528E+00 -.90924734811142304824E+00

.52733131967042701227E+00 .13047885453687719792E+01

.29234606862006819010E-02 -.65532761058816561134E-02

.82024047787665164966E-02

.63557190255699735904E+00 .38627373667268505297E+00

-.52328179992474172996E-02 .78220010250202448576E-02

.55959096352670959114E-03 -.14337835049378980234E-02

.24333733615539742193E-02

-.29622208189094284547E+00 -.13430698198462612258E+00

.44795016231694312347E+00 .70070458050168404895E+00

-.12487009554822230128E-02 .32593098400804055778E-02

-.44992708217204951254E-02

.25646042102109434246E+00 .58082711777469164804E+00

.33180329240305844740E+00

0/

END

FIT

7S 3.52

7P 3.04

6D 4.68

END

**Germanium (TZP, 3d frozen)**

BASIS

1S 16.95

2S 13.95

3S 5.95

2P 14.10

3P 6.15

3D 4.09

4S 1.25

4S 1.95

4S 3.15

4P 0.80

4P 1.35

4P 2.35

4D 1.6

END

CORE 3 2 1 0

1S 29.8500

1S 37.8000

2S 12.7000

2S 7.5500

3S 4.8500

3S 7.9500

4S 1.3000

4S 1.9500

4S 3.0500

2P 11.8500

2P 17.8500

3P 4.2000

3P 6.9000

4P 1.3500

4P 0.8000

4P 2.4000

3D 2.5000

3D 4.8000

3D 9.2000

END

DESCRIPTION

.60275901473250304008E+00 .39222137336885282144E+00

.94289264337138550820E-01 -.19336115333900971702E+00

-.26473361605203159230E-01 .14479324599299503440E+00

.29437943558091356384E-02 -.79733690965424486308E-02

.11638582495025756244E-01

-.49002609842823663389E+00 .91376075427298586451E-01

.10656485219808640164E+01 .10237857713060709297E+00

.32696836595599070019E-01 -.10078123756538759359E+00

-.47059252168196166397E-02 .12397061865603246217E-01

-.17098109884715207568E-01

.21390501702287337271E+00 -.51759341105718774390E-01

-.25586713617008544075E+00 -.87616887682454458464E+00

.53239971664691276843E+00 .12512959715712093089E+01

.38190828469915200083E-02 -.85362557316477488678E-02

.93007225443902021705E-02

.65078786799567334143E+00 .36874874879261704264E+00

-.69245807315714436730E-02 .11276642521803065949E-01

-.20499755826405272920E-02 .83029346582821916562E-03

.31382763756747250085E-02

-.30741414954169027363E+00 -.12789899559337664092E+00

.45264081038122205847E+00 .69440237567580109790E+00

.36282986418332088298E-02 -.14386094177158477870E-02

-.48640018029565516844E-02

.27114889499287031516E+00 .58245772480088398027E+00

.29663636213369465322E+00

0/

END

FIT

7S 2.00

7P 3.29

7P 2.05

6D 5.05

7D 3.51

7F 2.95

7G 3.20

END

**Hydrogen (TZP)**

BASIS

1S 0.69

1S 0.92

1S 1.58

2P 1.25

END

CORE 0 0 0 0

END

DESCRIPTION

END

FIT

1S 3.16

1S 1.38

2P 4.00

END

**Helium (TZP)**

BASIS

1S 1.15

1S 1.65

1S 3.05

2P 1.00

END

CORE 0 0 0 0

END

DESCRIPTION

END

FIT

1S 6.10

1S 4.41

2P 2.65

3D 4.00

3D 2.50

4F 3.00

END

**Hafnium (TZP, 4f frozen)**

BASIS

1S 50.000

2S 32.050

3S 18.400

4S 11.300

2P 36.150

3P 15.700

4P 11.300

3D 18.750

4D 10.400

4F 5.250

1S 158.100

5S 5.650

5S 3.650

6S 2.600

6S 1.650

6S 1.060

5P 5.550

5P 3.650

5P 2.450

5D 3.600

5D 1.850

5D .950

6P 1.650

END

CORE 4 3 2 1

1S 645.000

1S 158.100

1S 77.800

2S 86.400

2S 33.900

3S 43.350

3S 18.250

4S 11.150

4S 7.800

5S 5.650

5S 3.650

6S 2.600

6S 1.650

6S 1.060

2P 56.600

2P 32.600

3P 18.650

3P 14.000

4P 11.600

4P 7.750

5P 4.800

5P 2.900

3D 24.900

3D 15.750

4D 10.500

4D 6.450

5D 3.600

5D 1.850

5D .950

4F 12.250

4F 6.100

4F 2.850

END

DESCRIPTION

.18082298777973987436E-01 .11920803724371352439E+00

.89777984025268120494E+00 -.44868409075296874361E-02

-.24471336267801722421E-02 .25589414046696496927E-02

.80969961514960807846E-04 -.97846818406718857170E-04

.98808740215157675685E-04 -.59612000206557093156E-04

.36016280689361194285E-04 -.18789000283756405806E-04

.96619919305609112958E-05 -.34148955592965896419E-05

-.60300446649664335083E-02 -.47679897601569806720E-01

-.28315213890289819831E+00 -.88837062374715780599E-01

.97259794124329990161E+00 .12552982048335289966E+00

.37931585248098778802E-02 .39406324820574013855E-03

-.10445898548010730880E-02 .86761984249663422642E-03

-.58329171942291490032E-03 .31971376187802535594E-03

-.16807264955624327861E-03 .59955352511553439967E-04

.29320757907967563358E-02 .11160024046140873399E-01

.16179573201257194115E+00 -.11712776994157647961E-01

-.26883712852171320895E+00 -.40783506723088119861E+00

.11866362174015210318E+01 -.57445652115023539130E-02

.68930554162402856572E-02 -.28082194583835761413E-02

.12516802707031931749E-02 -.52807429631172874730E-03

.24157690185744784463E-03 -.80924495628011816747E-04

-.15248392347557739035E-02 -.54469547631277732462E-05

-.95731938573453356778E-01 .32555727219096677882E-01

.33616265634975425747E-01 .33163213446069955692E+00

-.85286659434900324861E+00 .10214666133818537386E+01

.24409741286927486015E+00 -.65399662178940295640E-02

.33544989503239705866E-02 -.19828910563531583315E-02

.11144325885152448364E-02 -.41186526797824666354E-03

.16192584948670063483E+00 .86136903092023153761E+00

.43289934176684108716E-02 -.73710141869106357917E-02

.64660674606540971543E-02 -.31754467679921041107E-02

.10113869378311826901E-02 -.38688401730632010489E-03

-.59120881110317484441E-01 -.50457014370773634049E+00

.94587784600541069402E+00 .23693038162690399639E+00

-.36345666677317201021E-01 .14323085740093596807E-01

-.43085253654746935928E-02 .16387113111455168472E-02

.24735249479524112209E-01 .25810350618698940650E+00

-.50220723342215889407E+00 -.37867720742575317061E+00

.89805573767929014473E+00 .47040215606037349971E+00

.37718003988990492746E-02 -.12297491991298556500E-02

.38596319665409783983E+00 .65738440517175655842E+00

-.53955851751864855950E-02 .39563617207175267101E-02

-.10697601378831837152E-02 .46904545285031651021E-03

-.16787806524652269715E-03

-.19943930968647854973E+00 -.31342104313262347803E+00

.74072364379166588666E+00 .43717260314714012637E+00

-.91825669864065278132E-05 .10889622696166813710E-02

-.43185179147231647417E-03

.40341510514843909041E+00 .59654282360910293459E+00

.21391389147608269594E+00

0/

END

FIT

10S 3.88

8P 6.71

10P 2.60

9D 5.42

9D 3.73

10D 2.01

9F 4.47

8G 5.19

9G 1.90

END

**Mercury (TZP, 4f frozen)**

BASIS

1S 58.000

2S 37.050

3S 21.400

4S 12.650

2P 40.750

3P 17.800

4P 12.950

3D 22.450

4D 11.400

4F 8.300

1S 188.400

5S 7.200

5S 4.800

6S 3.300

6S 2.050

6S 1.280

5P 6.850

5P 4.650

5P 3.200

5D 5.300

5D 3.050

5D 1.710

6P 2.050

END

CORE 4 3 2 1

1S 781.000

1S 188.400

1S 89.200

2S 101.600

2S 38.900

3S 49.500

3S 21.100

4S 12.950

4S 9.200

5S 7.200

5S 4.800

6S 3.300

6S 2.050

6S 1.280

2P 67.300

2P 37.150

3P 21.100

3P 15.450

4P 13.850

4P 9.450

5P 6.150

5P 3.850

3D 28.450

3D 18.200

4D 12.450

4D 8.050

5D 5.300

5D 3.050

5D 1.710

4F 16.600

4F 9.250

4F 5.200

END

DESCRIPTION

.22513325915168577035E-01 .14439922734080237743E+00

.86791626106452379741E+00 .10408392196657657858E-01

-.29968842122238374681E-01 .26184992522449806324E-01

.10155405593068090892E-02 -.71328739933446409956E-03

.66298823935477318656E-03 -.35374269844766568615E-03

.15733066782723883516E-03 -.60929399725794862008E-04

.28847595561562055529E-04 -.99763237157542864820E-05

-.76038564963347423861E-02 -.57149126769014617355E-01

-.27363510817773983863E+00 -.86241400328867207081E-01

.99536187914396878629E+00 .99679967205252795193E-01

.17334266235863140301E-02 .31603035885105724631E-02

-.42864536951340180201E-02 .29479997240021379480E-02

-.15205176562234495874E-02 .64360607342729919764E-03

-.31673847235201216125E-03 .11129373638445703987E-03

.36980152139674872004E-02 .17211092057227411062E-01

.15613653156714624171E+00 -.58146229706335858523E-02

-.32074106733187429663E+00 -.36670987844106994746E+00

.11953621712532089827E+01 -.11233220899528991582E-01

.11935635628839865591E-01 -.52314405638490303618E-02

.18635689125617877632E-02 -.59286910949164252360E-03

.25230270254423571574E-03 -.83173559218428384635E-04

-.19471101794743814802E-02 -.34393423909306712616E-02

-.94416366996062567329E-01 .28357315711420534587E-01

.71162410056148817095E-01 .31036707735001817232E+00

-.88351700763809193351E+00 .10577779431733043136E+01

.22111082262702336232E+00 -.13227437362150163899E-01

.62119948723930486223E-02 -.30558692890762222810E-02

.16384842642033700666E-02 -.59933269955369063760E-03

.15052976082258229251E+00 .87375955941839411611E+00

.10793551255241564182E-01 -.18584847854072800155E-01

.15230253358864563953E-01 -.63400091478658651342E-02

.19622314313572563824E-02 -.70979062786036685277E-03

-.58010075817926866348E-01 -.51149350321915909579E+00

.98432349595859791602E+00 .22062346442738905994E+00

-.69512667429874414049E-01 .24847946768938311557E-01

-.75343001991380323043E-02 .27329653164994377294E-02

.25589591885702731716E-01 .26695008119803292912E+00

-.50732857718599277952E+00 -.49945086083935213983E+00

.95349435698930118388E+00 .52557055377401651342E+00

.83656568138838188070E-02 -.28162959109551050953E-02

.37437325320320730127E+00 .66656888418134929708E+00

-.55954330773452251938E-02 .42595703097313012200E-02

-.11376631924606246064E-02 .39991781691265109074E-03

-.12041339403236801396E-03

-.19957496000914801138E+00 -.34671892029575773764E+00

.72066724809526805373E+00 .46402071757863838641E+00

-.43451985170868780758E-03 .13613435545118681650E-02

-.42188727592427623320E-03

.28150967443811875102E+00 .62397614235188025855E+00

.23595990567882418887E+00

0/

END

FIT

9S 7.28

10S 6.70

10S 4.67

9P 5.83

10P 4.91

10P 3.76

10D 2.99

9F 6.54

END

**Iodine (TZP, 4d frozen)**

BASIS

1S 34.200

2S 23.550

3S 10.450

4S 7.400

2P 24.100

3P 11.700

4P 6.600

3D 13.400

4D 4.600

5S 3.800

5S 2.500

5S 1.690

5P 3.100

5P 1.900

5P 1.190

END

CORE 4 3 2 0

1S 381.000

1S 95.600

1S 53.900

2S 61.350

2S 23.200

3S 12.250

3S 8.500

4S 8.550

4S 5.450

5S 3.800

5S 2.500

5S 1.690

2P 35.200

2P 22.300

3P 13.200

3P 9.900

4P 7.000

4P 4.600

5P 3.100

5P 1.900

5P 1.190

3D 21.700

3D 13.150

3D 9.000

4D 6.800

4D 4.350

4D 2.850

END

DESCRIPTION

.10862135295578343244E-01 .85420997577325796790E-01

.93239950831159845723E+00 -.14099026477701184779E-01

.12075224848296462008E-02 -.35800969913538645099E-02

.71159299618631663767E-02 -.50161845596488723042E-02

.99223953749937249605E-03 -.43432608295143604587E-03

.24523173121627410507E-03 -.83754677556093992609E-04

-.31132462954132698700E-02 -.92929074286319413023E-01

-.15930251735800676771E+00 -.20436905574662314611E+00

.11175593410388247051E+01 .84937127867954539256E-02

-.18980394858493194327E-01 .15769992243801850273E-01

-.44842530772231390182E-02 .22941099974477172085E-02

-.13826453168756993456E-02 .48713574343775176666E-03

.96042233393882608244E-03 .88593282892899027914E-01

-.34368571253722640657E-01 .19003316106647064054E+00

-.67489815400273456270E+00 .93333821935462712993E+00

.49797662574692408421E+00 -.34248344198452224107E+00

.82999544109666936742E-01 -.40782742440008963591E-01

.24382215451568201720E-01 -.85948482296648084228E-02

-.40581973569302817045E-03 -.45849327846305548695E-01

.26537836079577131754E-01 -.98297991983352914214E-01

.33172916912479666340E+00 -.26455998534314539850E+00

-.12590568388993286231E+01 .15034625470415432513E+01

.53949466023456937869E+00 .26388749855696849722E-01

-.16512314328048324774E-01 .65364023932163624331E-02

.22668191390731179391E+00 .79427265286898729446E+00

.41664650207575834248E-02 -.52160991093478225461E-02

.40933211922137158895E-02 -.27819738354046439840E-02

.13206731635743533537E-02 -.72745078053979591974E-03

.26468806156624652371E-03

-.77711751541994805725E-01 -.45306919224120378864E+00

.72951391275932142566E+00 .43185602570714304305E+00

-.11703267115547037053E-01 .68794188622714521480E-02

-.30679892375494442237E-02 .16722580318600484880E-02

-.60878998080583128852E-03

.29947477573155190100E-01 .21682627973776655295E+00

-.38913037577608833306E+00 -.30397519183980797886E+00

.82528759970235665566E+00 .40412214455173595118E+00

.17360924180495709325E-02 -.10197789587495997236E-02

.56901068958714292375E-03

.12207218677097519954E+00 .58177811545417124073E+00

.35437577761902711027E+00 -.22126459730724963408E-02

.94004702662284190477E-03 -.17594810756792157377E-03

-.48724628328488207851E-01 -.26893262086455277382E+00

-.10976111392553432322E+00 .49721411617841293484E+00

.53835304812021178567E+00 .12453556289217215802E+00

0/

END

FIT

5S 11.6400000000000

9S 4.18000000000000

9S 1.98000000000000

9S 2.67000000000000

7P 3.87000000000000

8P 6.23000000000000

8P 4.35000000000000

9P 3.86000000000000

8D 4.80000000000000

9D 3.12000000000000

8F 3.29000000000000

8G 2.71000000000000

END

**Indium (TZP, 4d frozen)**

BASIS

1S 32.600

2S 21.350

3S 9.950

4S 6.400

2P 22.250

3P 10.550

4P 5.800

3D 12.100

4D 3.400

5S 3.000

5S 1.900

5S 1.240

5P 2.300

5P 1.300

5P .750

END

CORE 4 3 2 0

1S 339.000

1S 84.700

1S 49.300

2S 57.600

2S 21.100

3S 10.900

3S 6.450

4S 7.000

4S 4.500

5S 3.000

5S 1.900

5S 1.240

2P 31.500

2P 20.250

3P 12.100

3P 8.900

4P 6.000

4P 3.750

5P 2.300

5P 1.300

5P .750

3D 19.250

3D 11.600

3D 7.800

4D 5.550

4D 3.250

4D 1.900

END

DESCRIPTION

.95585887701911155523E-02 .78316550437869239532E-01

.94379086654921329202E+00 -.19816413270296338867E-01

.87874839318756870887E-03 -.19275991267395911535E-02

.55322986395264254697E-02 -.46411689760591737924E-02

.58013853680608526778E-03 -.24210894741156225424E-03

.13792292028263754234E-03 -.49404037012049793656E-04

-.25583601346260238449E-02 -.13696169925689530289E+00

-.64486778698484570338E-01 -.25700584489110250841E+00

.11132633559977942461E+01 .46106341970898008162E-02

-.14394474450160375106E-01 .13666068781907446741E-01

-.27621720081232002675E-02 .13318438612706358457E-02

-.80387557735375802029E-03 .29548300088578068274E-03

.67183080320821586366E-03 .12977519125927142185E+00

-.12201377277264845167E+00 .23506744414909425123E+00

-.65793658587518477621E+00 .10878226053768278447E+01

.22778263315971081227E+00 -.18787097727159640659E+00

.35289974428782629190E-01 -.17762130540343017832E-01

.10914165446071910290E-01 -.40541978022151977518E-02

-.24556635528401153396E-03 -.66546318853973468466E-01

.72907543051474274498E-01 -.11989888152145689038E+00

.31284302355988380384E+00 -.49658471904317036394E+00

-.87880374500149993633E+00 .14687928986535752074E+01

.45190731331763611678E+00 .17690467312975875769E-01

-.10885756534353467628E-01 .44552202006967189796E-02

.24600753189463220316E+00 .77446368089626638209E+00

.44814349583558151305E-02 -.43665123355548221204E-02

.26155716768170183574E-02 -.15188899415512432684E-02

.61998898007022769287E-03 -.32829727853550571322E-03

.12434747886005284123E-03

-.83759478145488869272E-01 -.43608552292918983939E+00

.68592926798916165065E+00 .47129248120666022981E+00

-.61788791321608464713E-02 .33832829159836886809E-02

-.13003723673743591751E-02 .68175158727528501444E-03

-.25810356248389017264E-03

.30375368179331466156E-01 .19920133683615004472E+00

-.36262882637346216530E+00 -.26280589252468172790E+00

.81863736215216664771E+00 .38118693528070113352E+00

-.95450928772225062530E-03 .10034118329140218843E-02

-.33196463371298445388E-03

.13978362866367188833E+00 .58606354521590109385E+00

.33810193582058561956E+00 -.18513900211329563459E-02

.93281707352306628047E-03 -.24443103592197603768E-03

-.47962489236020595940E-01 -.24381444113985595301E+00

-.59190986299993038344E-01 .51263626303722675814E+00

.51918544299010771503E+00 .13540674624387696867E+00

0/

END

FIT

8S 3.79

9S 1.84

8P 3.35

9P 2.72

8D 4.22

9D 2.18

END

**Iridium (TZP, 4f frozen)**

BASIS

1S 55.000

2S 35.000

3S 20.200

4S 12.200

2P 38.650

3P 17.250

4P 12.200

3D 21.400

4D 10.750

4F 7.300

1S 176.600

5S 6.600

5S 4.400

6S 3.100

6S 1.950

6S 1.230

5P 6.300

5P 4.250

5P 2.950

5D 4.800

5D 2.700

5D 1.480

6P 1.950

END

CORE 4 3 2 1

1S 728.000

1S 176.600

1S 84.800

2S 95.400

2S 37.000

3S 47.150

3S 20.000

4S 12.250

4S 8.700

5S 6.600

5S 4.400

6S 3.100

6S 1.950

6S 1.230

2P 63.200

2P 35.450

3P 20.150

3P 14.950

4P 13.000

4P 8.800

5P 5.650

5P 3.500

3D 27.100

3D 17.300

4D 11.750

4D 7.400

5D 4.800

5D 2.700

5D 1.480

4F 15.150

4F 8.250

4F 4.450

END

DESCRIPTION

.20719854153474472069E-01 .13471063977535932543E+00

.87927698163178213964E+00 .51883363477061884839E-02

-.21167708373180559728E-01 .18561714219030411854E-01

.78030472766962160908E-03 -.60484351045625840779E-03

.56552418613421207624E-03 -.31364298476834026781E-03

.16169275933137027923E-03 -.69748947706694150949E-04

.33172610529736338669E-04 -.11334283537762679422E-04

-.69681912241711888717E-02 -.53159250372939760576E-01

-.27831966649523670609E+00 -.86763641457487661324E-01

.98434879929518170893E+00 .11185701775841495886E+00

.31634979409841871949E-02 .13390572774896933837E-02

-.22020204992212319070E-02 .16778247649609303888E-02

-.10027664354791555445E-02 .47003568521157887807E-03

-.23214985773071790225E-03 .80590939643257841307E-04

.33881507800157186112E-02 .14877606564052894486E-01

.15846333072473087200E+00 -.82040822896082936377E-02

-.29932193514727328809E+00 -.38422741474690080832E+00

.11925807756992876651E+01 -.10812100232274078279E-01

.11342920382374766658E-01 -.49970602929902824263E-02

.20816781871144198791E-02 -.75617484721609673059E-03

.32706318303176980331E-03 -.10702873799032159620E-03

-.17743492371937407120E-02 -.21657099777107739237E-02

-.94799408395411691175E-01 .30064081591454180215E-01

.55323645577581299915E-01 .31977831993622851670E+00

-.87174281761892358222E+00 .10455908538755693815E+01

.22523409616592013349E+00 -.60509251248834121720E-02

.33655967577457986117E-02 -.20038945376360233624E-02

.11240067092324077056E-02 -.41336903601328059323E-03

.15350396445602967899E+00 .86987120703021880708E+00

.10353793631250749613E-01 -.16195420123794171396E-01

.12533746851725877450E-01 -.54148945223181834535E-02

.16840996533627799938E-02 -.61798848401348389393E-03

-.57926845304134430559E-01 -.50920079717142630038E+00

.97561258624359192826E+00 .21710364064549431129E+00

-.52604907197900230287E-01 .20135571401332371527E-01

-.62006679840727182626E-02 .22893911028166427819E-02

.25038669214954695913E-01 .26358250290217488665E+00

-.50766307600835225422E+00 -.44414703976902741855E+00

.92064397470388559253E+00 .50918853615254489053E+00

.65544996893130780605E-02 -.21903223883346341697E-02

.37809009567606094437E+00 .66252092613213642291E+00

-.36957854514042352140E-02 .34516214240326659538E-02

-.97755080273666445680E-03 .35666582127675264735E-03

-.11211149508735637661E-03

-.19821072056482025303E+00 -.33680180074277937807E+00

.72609705737188268149E+00 .46077657287582507450E+00

-.55142896259635634446E-02 .35378562850767779693E-02

-.12036344240063646081E-02

.30961257931380870012E+00 .61315362009304030799E+00

.23660728852432588720E+00

0/

END

FIT

9S 6.98

10S 4.48

11S 3.47

9P 7.08

9P 5.34

10P 3.43

9D 6.92

9D 4.85

10D 3.80

END

**Potassium (TZP, 3p frozen)**

BASIS

1S 11.85

2S 7.50

3S 3.00

2P 6.83

3P 2.33

4S 0.55

4S 0.85

4S 1.45

4P 0.80

3D 1.40

3D 3.20

END

CORE 3 2 0 0

1S 23.0500

1S 17.2500

2S 7.0000

2S 4.7500

3S 3.6000

3S 2.2500

4S 0.8500

4S 0.5500

4S 1.4500

2P 10.0500

2P 5.9500

3P 1.5000

3P 2.2500

3P 3.5500

END

DESCRIPTION

.26760579311276638803E+00 .73625345597681746135E+00

.16515468843332586218E-01 -.19606436123600912147E-01

.11711015129095642129E-01 -.60692021958255520583E-02

-.15462541041482754517E-02 .62299778687714604114E-03

.25621437073274273312E-02

.60921097463194254229E-01 -.42033035012665198593E+00

.10020988592266029116E+01 .89423138431008533655E-01

-.10783923685185438715E-01 .70036632145786441653E-02

.24535551353250008300E-02 -.10238097195962856441E-02

-.37118420171451988084E-02

-.30897198314051486923E-01 .16096244778593471469E+00

-.32852845376274197875E+00 -.22785610957507040908E+00

.77117027888848044270E+00 .44986873872468535751E+00

-.50576050317405699813E-02 .23724840261137780315E-02

.76072560040749363092E-02

.37770438598593586432E+00 .65869142494704779889E+00

.23343430070244565455E-02 -.46704542590893438278E-02

.54889623744417423770E-02

-.11468325619140183613E+00 -.24353124208809051598E+00

.14946655648107032288E+00 .54784231302304042810E+00

.43764780452684332035E+00

0/

END

FIT

4S 15.44

4S 10.43

5S 6.18

6S 3.76

6S 2.72

7S 1.69

7S 1.25

5P 4.83

6P 3.21

6P 1.84

7P 1.25

4D 8.81

5D 5.81

6D 3.91

6D 2.26

7D 1.55

END

**Krypton (TZP, 3d frozen)**

BASIS

1S 23.45

2S 14.50

3S 7.15

2P 16.30

3P 7.00

3D 5.73

4S 1.75

4S 2.55

4S 3.80

4P 1.30

4P 2.10

4P 3.40

4D 1.8

END

CORE 3 2 1 0

1S 43.6000

1S 33.9500

2S 14.3500

3S 7.3500

3S 5.0000

4S 1.7500

4S 2.5500

4S 3.9000

2P 19.8500

2P 13.5000

3P 8.1500

3P 5.3000

4P 1.3000

4P 2.1000

4P 3.4500

3D 3.8500

3D 6.5500

3D 11.6000

END

DESCRIPTION

.35518138696592138626E+00 .64591342024517917064E+00

.12946981535064627789E-01 -.22120640841593687542E-01

.27282232859021128546E-01 -.37144163109711155098E-02

.10628298940950811624E-01 -.18496410489401284710E-01

.76514514317406065458E-01 -.47724150337867682348E+00

.11155502517614661606E+01 -.73066444380663328473E-01

.81077548470398108638E-01 -.94736793614685638892E-02

.27669288350689810740E-01 -.50549610738249928232E-01

-.70584424749510654706E-01 .24978128590475837112E+00

-.59059711670625869573E+00 .10540244439939778776E+01

.78094297398440315483E-01 .83217700399763807478E-02

-.25446076551568320295E-01 .47714077159100788672E-01

.41196508723628005866E+00 .60331428876716797216E+00

.21336058845525082001E-01 -.17566726664330276075E-01

.21544715148025402572E-02 -.58717662259152704832E-02

.93356107674716134331E-02

-.14651013331824108699E+00 -.31403610018124328285E+00

.67156786645963106874E+00 .47568759936737864402E+00

-.92747400529561612251E-03 .27907161810287522616E-02

-.38509844364896421778E-02

.29408217981233775173E+00 .57947262433884227928E+00

.23499241776363113954E+00

0/

END

FIT

6S 11.91

6S 9.19

6S 7.10

5P 14.76

6P 10.61

6P 6.56

7P 3.05

7D 3.40

7F 3.90

END

**Lithium (TZP, 1s frozen)**

BASIS

1S 2.46

2S 0.46

2S 0.68

2S 2.36

2P 0.60

2P 1.20

3D 1.2

END

CORE 1 0 0 0

1S 4.24

1S 2.26

2S 0.68

2S 0.46

2S 2.36

END

DESCRIPTION

.19725346782302047299E+00 .84201802789104562841E+00

.58775752485345032078E-02 -.34378414960204964519E-02

-.21801364188416817030E-01

0/

END

FIT

1S 4.92

2S 3.18

3S 1.36

2P 4.72

2P 2.39

2P 1.21

3P .92

END

**Magnesium (TZP, 2p frozen)**

BASIS

1S 10.25

2S 3.85

2P 3.32

3S 0.75

3S 1.10

3S 1.65

3P 1.06

3D 1.50

END

CORE 2 1 0 0

1S 10.5500

1S 14.6500

2S 2.9000

2S 4.4000

3S 1.1000

3S 0.7500

3S 1.7000

2P 2.3500

2P 3.9000

2P 7.1000

END

DESCRIPTION

.74244066469460734403E+00 .26429239203000493008E+00

-.49405551943441259818E-02 .53304398297620023475E-02

-.26837961362488117235E-02 .10627377957177009445E-02

.30097083576436042297E-02

-.34047951296346684602E+00 .27330869777920221986E-01

.42135358196886818494E+00 .66714538890434160745E+00

.43371951496516724170E-02 -.17833963973482194580E-02

-.46834954439057040546E-02

.33489636594351535059E+00 .55515570906963151998E+00

.18730028517876007310E+00

0/

END

FIT

5S 2.14

4P 6.31

4P 3.93

5P 3.07

5P 2.00

5D 3.08

END

**Manganese (TZP, 3p frozen)**

BASIS

1S 15.50

2S 10.10

3S 4.50

2P 9.77

3P 3.78

3D 1.32

3D 2.85

3D 5.95

4S 0.85

4S 1.35

4S 2.20

4P 1.35

END

CORE 3 2 0 0

1S 29.6500

1S 23.0000

2S 9.6500

2S 6.7500

3S 5.6500

3S 3.5000

4S 0.8800

4S 1.3600

4S 2.2000

2P 13.8000

2P 8.7000

3P 4.9500

3P 2.9500

END

DESCRIPTION

.32812736478534848539E+00 .67163117591017096331E+00

.48772167564980588306E-01 -.63397615657635977837E-01

.35873368270834080085E-01 -.14240737376620243512E-01

.14290877819724586827E-02 -.38346233237523705099E-02

.59693368274400995105E-02

.85970826926584545391E-01 -.47004025717418751462E+00

.10224766505367888225E+01 .98408601114478147509E-01

-.47679215096342368596E-01 .24501811865036870336E-01

-.30924729526469242093E-02 .80903335391655280956E-02

-.11932317548030212800E-01

-.45260404305791694657E-01 .19652743824439869447E+00

-.27395299254067334571E+00 -.46122999883535537036E+00

.86145863642255970927E+00 .50705352422620231234E+00

.24991877014567840873E-02 -.55488086406755903965E-02

.70730694811733585067E-02

.35392547394500029068E+00 .67397726115414502424E+00

.30160648167363514512E-02 -.11191983957391791246E-02

-.12034660890705846381E+00 -.29291125293782954708E+00

.69650129733041499236E+00 .44338671215678227000E+00

0/

END

FIT

7S 3.11

6P 5.73

6P 3.38

6D 6.81

6F 2.67

5G 10.40

5G 2.97

END

**Molybdenum (TZP, 4p frozen)**

BASIS

1S 30.500

2S 17.750

3S 8.350

4S 4.850

2P 18.600

3P 8.700

4P 3.550

3D 10.050

5S 2.150

5S 1.350

5S .870

4D 3.500

4D 1.850

4D .970

5P 1.350

END

CORE 4 3 1 0

1S 269.000

1S 67.100

1S 41.500

2S 49.500

2S 17.550

3S 9.000

3S 5.900

4S 5.100

4S 3.300

5S 2.150

5S 1.350

5S .870

2P 25.800

2P 16.750

3P 10.150

3P 7.050

4P 4.950

4P 3.300

4P 2.300

3D 15.300

3D 9.000

3D 5.750

4D 3.500

4D 1.850

4D .970

END

DESCRIPTION

.76334613097897088818E-02 .57142759428960576695E-01

.98382931106779270003E+00 -.42638706576629284428E-01

.32510209146898193027E-03 -.64095131075382920025E-03

.96874272845557469949E-03 -.72723183520210967889E-03

.29614600545213527793E-03 -.12260379067867996448E-03

.71979669834034006818E-04 -.26197628303502755112E-04

-.17762572778005048749E-02 -.27241146493684248453E+00

.19745852315159354018E+00 -.39072707288866898034E+00

.11055538038140106938E+01 -.17489110007982831179E-03

.56335629971665111999E-03 .60539849641184700565E-03

-.70573650316707776681E-03 .42123846501289461276E-03

-.27675313501048849109E-03 .10552187949892146692E-03

.29374783181610326617E-03 .25081932945070550822E+00

-.35539093643241098652E+00 .34707731012940518101E+00

-.62182509937508423370E+00 .10492093411272593872E+01

.13433154608832886812E+00 -.42449380948486277465E-01

.18644492110042498040E-01 -.91254050700881206987E-02

.57529070428141296581E-02 -.21668647913725227507E-02

-.67037347291918681670E-04 -.11964806882756230666E+00

.17878278843493103367E+00 -.16462429261999342311E+00

.27254056429960032970E+00 -.51624619576709562807E+00

-.20552550364504704183E+00 .88451818738606280412E+00

.39785421772843837518E+00 .11144414207401582076E-01

-.68391810491806523387E-02 .28460880996563960726E-02

.27619288264378916153E+00 .74521287232978961068E+00

.34735072184724625870E-02 -.37224806901170639888E-02

.34213301523768864867E-02 -.27888739497818155685E-02

.10902871815906206923E-02

-.92838540645612477320E-01 -.40286611151403772801E+00

.63101033827187613667E+00 .52559783605427357056E+00

-.92319059681401222095E-02 .65905667328579236111E-02

-.22784281744898230750E-02

.32789671297968579766E-01 .16213427796113541457E+00

-.27901330293423781681E+00 -.27765205222477062907E+00

.55979533180832352635E+00 .52145806663600258180E+00

.11273656286260590187E+00

.17132863338490275651E+00 .58547094797331755700E+00

.32288127477676126231E+00 -.11309789342647642244E-02

.73880485703257481409E-03 -.24588455112233654825E-03

0/

END

FIT

8S 3.65

7P 9.73

7P 6.73

9P 2.17

7D 3.88

END

**Nitrogen (TZP, 1s frozen)**

BASIS

1S 6.38

2S 1.50

2S 2.50

2S 5.15

2P 1.00

2P 1.88

2P 3.68

3D 2.2

END

CORE 1 0 0 0

1S 8.74

1S 5.90

2S 1.50

2S 2.50

2S 5.15

END

DESCRIPTION

.26662918423848019911E+00 .73718698062977017216E+00

.22942877984330365890E-02 -.51067583084347433278E-02

.11469962265938677543E-01

0/

END

FIT

2S 15.7700000000000

3S 6.02000000000000

3S 2.40000000000000

3S 3.29000000000000

3P 2.70000000000000

3P 2.44000000000000

3D 4.96000000000000

3D 2.47000000000000

END

**Sodium (TZP, 2p frozen)**

BASIS

1S 9.45

2S 3.50

2P 2.78

3S 0.55

3S 0.80

3S 1.30

3P 0.79

3D 1.2

END

CORE 2 1 0 0

1S 9.6000

1S 13.4500

2S 2.5500

2S 4.0500

3S 0.8000

3S 0.5500

3S 1.3500

2P 1.9000

2P 3.3000

2P 6.1000

END

DESCRIPTION

.74107921492364825511E+00 .26635983054102563283E+00

-.28109434125583305890E-02 .34240475636464753548E-02

-.13859652697210845130E-02 .64848068012180056473E-03

.14527050498106843218E-02

-.32706242013553921133E+00 .22817286425688396850E-01

.46836549632496182749E+00 .62378992565274304916E+00

.55599942423279515025E-02 -.26763330252176859361E-02

-.50837822779752125241E-02

.32808209266291538109E+00 .54001291849944088597E+00

.22373557630192675294E+00

0/

END

FIT

4S 2.85

5S 2.35

5S 1.61

3P 12.57

3P 7.50

4P 5.98

4P 3.79

5P 3.02

5P 2.00

5D 3.00

END

**Niobium (TZP, 4p frozen)**

BASIS

1S 28.400

2S 17.250

3S 8.300

4S 4.600

2P 18.050

3P 8.500

4P 3.350

3D 9.750

5S 2.050

5S 1.300

5S .850

4D 3.250

4D 1.700

4D .880

5P 1.300

END

CORE 4 3 1 0

1S 260.000

1S 64.600

1S 40.400

2S 47.000

2S 17.100

3S 8.850

3S 6.150

4S 4.750

4S 3.100

5S 2.050

5S 1.300

5S .850

2P 25.000

2P 16.250

3P 9.800

3P 6.750

4P 4.700

4P 3.150

4P 2.200

3D 14.700

3D 8.600

3D 5.450

4D 3.250

4D 1.700

4D .880

END

DESCRIPTION

.72966384236899349547E-02 .72111657122554767563E-01

.95476944257065088362E+00 -.26750524451562781442E-01

.76325040654156130551E-03 -.11211804458008556989E-02

.13379700828599161520E-02 -.85283887740898289397E-03

.43941916434815035241E-03 -.19670946064110570719E-03

.11786770508050677174E-03 -.43081289643668868193E-04

-.18239159934980272906E-02 -.19975080528344754671E+00

.45068577086098036977E-01 -.30356728039621799775E+00

.11118713656773555609E+01 -.49493248629462046825E-02

.76865660721254554927E-02 -.45339381828624807438E-02

.21270798717893976519E-02 -.88098275800681539929E-03

.51085438686451984172E-03 -.18397398010269746887E-03

.31039131862614280756E-03 .19647962408945110480E+00

-.24002729318697732519E+00 .28057297818212872942E+00

-.62604951921805229720E+00 .99202989322877177525E+00

.18558690420891663098E+00 -.32928024349931327153E-01

.18099402771920999888E-01 -.92049166298845946227E-02

.58581323049776883385E-02 -.22070536774776751410E-02

-.68702776519532994439E-04 -.93640432449963589834E-01

.12449203627475735612E+00 -.13240523492622202628E+00

.27039704733219249722E+00 -.49134615663404762609E+00

-.15958435838343207336E+00 .85304224026076747034E+00

.36157360341718458585E+00 .13839177610551874065E-01

-.92346973294758546297E-02 .38299093976974824156E-02

.28227715278261250598E+00 .73943242014858379108E+00

.29873499306267898602E-02 -.32001262476364072257E-02

.31161211678309365565E-02 -.26148753550518798357E-02

.10255214912186210591E-02

-.95415290999553162288E-01 -.39524355002309036200E+00

.64261658979634395106E+00 .51151217318367403486E+00

-.63037309294038642096E-02 .44603657789451332111E-02

-.14576413655789917465E-02

.32912836742218426478E-01 .15574686709556559427E+00

-.28155434400100814996E+00 -.25266477009961829658E+00

.54351526586266218910E+00 .51546568592149322185E+00

.12406746565205281629E+00

.17938326655418104782E+00 .58506473933015257227E+00

.31827652465636929735E+00 -.55875769670768189300E-03

.51925282834693867098E-03 -.17342505027098569837E-03

0/

END

FIT

8S 3.54

9S 2.10

8P 5.23

9P 2.15

8D 1.73

END

**Neon (TZP, 1s frozen)**

BASIS

1S 8.60

2S 1.65

2S 2.50

2S 4.15

2P 1.45

2P 2.85

2P 5.50

3D 2.0

END

CORE 1 0 0 0

1S 8.70

1S 12.45

2S 1.55

2S 2.20

2S 3.65

END

DESCRIPTION

.76211521529961934185E+00 .24608546469791264810E+00

.16447336455898673747E-02 -.32649588811284138180E-02

.26193842811583160218E-02

0/

END

FIT

2S 10.03

2S 6.03

3S 3.49

3S 2.27

3S 1.48

2P 7.42

3P 5.84

3P 3.40

END

**Nickel (TZP, 3p frozen)**

BASIS

1S 15.25

2S 12.00

3S 5.10

2P 11.22

3P 4.38

3D 1.54

3D 3.35

3D 7.00

4S 0.95

4S 1.50

4S 2.50

4P 1.47

END

CORE 3 2 0 0

1S 33.0000

1S 25.9000

2S 10.9500

2S 7.1500

3S 6.6000

3S 4.0000

4S 0.9400

4S 1.4800

4S 2.4500

2P 15.4500

2P 10.0000

3P 5.8500

3P 3.4500

END

DESCRIPTION

.36083602966718825167E+00 .63714039075595796380E+00

.59669692423279692417E-01 -.89530165304006967264E-01

.57158904469877008792E-01 -.16655394829189196920E-01

.15471742712473946727E-02 -.40804790491018394852E-02

.64534378929262071686E-02

.92486498630352673156E-01 -.48466950845350598165E+00

.10558959600145960689E+01 .74449822391854894588E-01

-.53722806466350685184E-01 .22773198665799236629E-01

-.27341331772226390440E-02 .70298928120663285030E-02

-.10498250269397583004E-01

-.50606558577175504710E-01 .20727878237809413409E+00

-.28777969840447237271E+00 -.55778337449423498473E+00

.97940983400025871664E+00 .50449855617685179787E+00

.19120167355766535134E-02 -.40602174577290955915E-02

.48503539760956599844E-02

.37007925041607819816E+00 .65474906734949966935E+00

.43302607012051198485E-02 -.19225572485214819079E-02

-.12423606893646135796E+00 -.30139689628554661160E+00

.68367105559313745555E+00 .46613358219408129735E+00

0/

END

FIT

4S 32.61

6S 7.84

6S 5.65

7S 4.75

3P 22.02

5P 9.48

6P 6.41

6P 3.74

4D 18.05

6D 7.68

6D 4.37

5F 5.07

6F 3.01

5G 12.30

5G 6.55

END

**Oxygen (TZP, 1s frozen)**

BASIS

1S 7.36

2S 1.72

2S 2.88

2S 7.58

2P 1.12

2P 2.08

2P 4.08

3D 2.00

END

CORE 1 0 0 0

1S 9.80

1S 6.80

2S 1.72

2S 2.88

2S 7.58

END

DESCRIPTION

.34739631610797133687E+00 .61176611867855479332E+00

.19065498074594599762E-02 -.36240996891512416989E-02

.60900331729172738071E-01

0/

END

FIT

2S 6.48000000000000

3S 3.20000000000000

3S 2.24000000000000

2P 8.85000000000000

3P 5.22000000000000

3P 4.27000000000000

3P 2.84000000000000

3D 4.02000000000000

3D 6.13000000000000

3D 2.08000000000000

4F 4.90000000000000

END

**Osmium (TZP, 4f frozen)**

BASIS

1S 54.000

2S 34.500

3S 19.850

4S 12.000

2P 38.050

3P 17.000

4P 11.950

3D 20.900

4D 10.650

4F 6.950

1S 173.500

5S 6.400

5S 4.250

6S 3.000

6S 1.900

6S 1.210

5P 6.150

5P 4.150

5P 2.900

5D 4.550

5D 2.550

5D 1.400

6P 1.900

END

CORE 4 3 2 1

1S 713.000

1S 173.500

1S 83.400

2S 93.400

2S 36.350

3S 46.400

3S 19.650

4S 12.000

4S 8.500

5S 6.400

5S 4.250

6S 3.000

6S 1.900

6S 1.210

2P 61.850

2P 34.900

3P 19.850

3P 14.700

4P 12.750

4P 8.600

5P 5.500

5P 3.400

3D 26.650

3D 17.000

4D 11.500

4D 7.200

5D 4.550

5D 2.550

5D 1.400

4F 14.650

4F 7.850

4F 4.150

END

DESCRIPTION

.20067677322641194054E-01 .13010224804244646668E+00

.88533869133124498685E+00 .21108841674474204501E-02

-.16405084687443675234E-01 .14550980174942773626E-01

.60244175232495829949E-03 -.46532360702074943796E-03

.43347736511366666019E-03 -.24174403140971977705E-03

.12794735463210186901E-03 -.57393264344819028462E-04

.28055122356178264337E-04 -.96460620455777130241E-05

-.67373022395606704804E-02 -.51353213667904261397E-01

-.28054770630122510822E+00 -.86984600824737565161E-01

.98516202287950704886E+00 .11195241733551196395E+00

.26484106310121322166E-02 .18198566472879735591E-02

-.26381693370233576766E-02 .19191414410066338480E-02

-.11497711536347368297E-02 .55288742595264604631E-03

-.27897372789463498839E-03 .97230837652805316394E-04

.32768978681400984070E-02 .13998771424354513465E-01

.15935730834319539184E+00 -.91953059135238129906E-02

-.29166982382500256099E+00 -.39055961965749930176E+00

.11909349171285110724E+01 -.82251203890560970616E-02

.90072938090589270421E-02 -.37576894347230577198E-02

.14993101533634274353E-02 -.52793292332488925625E-03

.22380772292547785642E-03 -.71842601918463715096E-04

-.17125362467657026695E-02 -.18417825868055185973E-02

-.94651751200091227556E-01 .30425980304613589600E-01

.49974178831802822109E-01 .32288231732146194242E+00

-.86658909531352401601E+00 .10471468067046518780E+01

.21969716281564671823E+00 -.32253483526354220084E-02

.18601743461268713340E-02 -.13390427451851608966E-02

.81346377010836981580E-03 -.30714113912939960309E-03

.15457341294793544573E+00 .86812729226987039866E+00

.11953636554066190620E-01 -.17845070697340932597E-01

.13193422293501485057E-01 -.55437561993010838271E-02

.17096649977294043656E-02 -.62868487414756315905E-03

-.57864690815259051115E-01 -.50825013666614149432E+00

.96842271383669731311E+00 .22490973274268200033E+00

-.53276738074540169743E-01 .19869340351501823400E-01

-.60909366601307034922E-02 .22572436532626578669E-02

.24856018002525730182E-01 .26233105011675628138E+00

-.50425451443556457143E+00 -.44034992889605967248E+00

.91876738252984535560E+00 .50635079106931846216E+00

.64145254066594950548E-02 -.21098519001917677429E-02

.37950183258560810096E+00 .66099301593389014897E+00

-.30167648049555322552E-02 .31220682636348196827E-02

-.88002588296554084320E-03 .34330253384570546225E-03

-.11158691800545269982E-03

-.19828550796797034250E+00 -.33208224051488199891E+00

.72846410079288193007E+00 .45675354921090349780E+00

-.50032248204184446780E-02 .34581105634263679775E-02

-.12158760146710526334E-02

.32284326405167318441E+00 .61457407227045379727E+00

.22951828625973197395E+00

0/

END

FIT

10S 5.27

11S 3.41

11S 2.87

8P 8.18

10P 4.32

9D 6.70

9F 3.90

END

**Phosphorus (TZP, 2p frozen)**

BASIS

1S 12.55

2S 4.90

2P 5.70

3S 1.30

3S 1.85

3S 2.65

3P 0.85

3P 1.30

3P 2.05

3D 1.90

END

CORE 2 1 0 0

1S 13.35

1S 18.00

2S 4.05

2S 5.60

3S 1.30

3S 1.85

3S 2.70

2P 4.10

2P 7.45

3P 0.85

3P 2.30

3P 1.35

END

DESCRIPTION

.71814449386934442465E+00 .28672379719271789966E+00

-.15756790888089990121E-01 .14522903623768375694E-01

.27748042833210966908E-02 -.78369912324877648996E-02

.96040326329322675547E-02

-.38152436071926820738E+00 .43342101363109356016E-01

.35920283169204875184E+00 .73285539720200665315E+00

-.65945747039918116428E-02 .16853350323540537692E-01

-.17924397712822167511E-01

.62772509162240142100E+00 .42230949798084083113E+00

.61425605764475722185E-03 .26912320220878176852E-02

-.97668185655873569241E-03

0/

END

FIT

4S 7.57000000000000

5S 3.01000000000000

5S 1.47000000000000

3P 4.23000000000000

4P 5.24000000000000

4P 5.40000000000000

5P 5.27000000000000

5P 2.25000000000000

5D 3.43000000000000

5D 1.80000000000000

END

**Lead (TZP, 5d frozen)**

BASIS

1S 60.000

2S 37.900

3S 22.100

4S 13.150

5S 7.550

2P 38.650

3P 20.600

4P 12.450

5P 6.900

3D 23.450

4D 11.600

5D 3.850

4F 8.950

1S 196.000

6S 3.700

6S 2.400

6S 1.580

6P 2.700

6P 1.600

6P .960

END

CORE 5 4 3 1

1S 816.000

1S 196.000

1S 92.200

2S 106.000

2S 40.200

3S 51.150

3S 21.850

4S 13.400

4S 9.550

5S 7.650

5S 5.200

6S 3.700

6S 2.400

6S 1.580

2P 70.200

2P 38.300

3P 21.750

3P 15.900

4P 14.500

4P 9.900

5P 6.600

5P 4.200

6P 2.700

6P 1.600

6P .960

3D 29.400

3D 18.800

4D 13.000

4D 8.450

5D 6.000

5D 3.650

5D 2.200

4F 17.560

4F 9.950

4F 5.750

END

DESCRIPTION

.23884813231338807016E-01 .15231008429703191132E+00

.85795681215695540622E+00 .14993251038608941539E-01

-.36339556863077590587E-01 .31685835021763017472E-01

.12221571428106116179E-02 -.87370676601257728916E-03

.85188006225625620329E-03 -.48510410889498768163E-03

.22292485097990673531E-03 -.91326215121888401150E-04

.44672707774762438588E-04 -.15069887108757511464E-04

-.80917302576616594728E-02 -.60379754831038905116E-01

-.26951449205209426774E+00 -.86016465076592454886E-01

.10017377360788006779E+01 .92259272025571700815E-01

.89560758882634172208E-03 .46988866116235592993E-02

-.64613858122066006318E-02 .47077853606932121983E-02

-.25343311165229205628E-02 .11494040424795566851E-02

-.59034286366019381089E-03 .20362890006273752595E-03

.39363051113262157929E-02 .18797071163273275374E-01

.15468204690890238751E+00 -.48566294324983820541E-02

-.33255852651924366903E+00 -.35651445400256021045E+00

.11963342420760729734E+01 -.10815849332076131126E-01

.11694020369363853154E-01 -.50822555202130114890E-02

.16272511774497017654E-02 -.43954445424000703662E-03

.15644861275299260959E-03 -.43415405865617268306E-04

-.20787408398553097917E-02 -.42639062708485610600E-02

-.94273132389307387480E-01 .27500203295083919947E-01

.80935644226779471233E-01 .30400710527614582812E+00

-.88952618782171988254E+00 .10709976318514555782E+01

.21103234982491214655E+00 -.17472583849674185041E-01

.95270656424138455698E-02 -.51977460263887952790E-02

.29551713504390453302E-02 -.10721572968510340029E-02

.98025748402902563097E-03 .10051949420130882468E-02

.46690623759478994126E-01 -.17382195783314090265E-01

-.21253386982342463429E-01 -.16247694532427153780E+00

.45446017491621865103E+00 -.70472902808663540064E+00

-.95006522829643530459E-01 .87039935868011131248E+00

.34927987550536787609E+00 .10929124422970367195E-01

-.54343064163940362973E-02 .22058794049693536236E-02

.14846456291274753858E+00 .87645916596048756286E+00

.12467849372363186758E-01 -.23019668628495044277E-01

.19563883254615607038E-01 -.84153086890889142180E-02

.32348045254781856821E-02 -.16627936026456353012E-02

.68759501509742563478E-03 -.35195795372091671265E-03

.13101120985356236662E-03

-.58121257616812672941E-01 -.51255613875438432725E+00

.97921436238942693997E+00 .23890222550438405813E+00

-.89926548531243349904E-01 .33287415790965217688E-01

-.12478305205087360588E-01 .64102512746357660084E-02

-.26618804084063531559E-02 .13669857459425074474E-02

-.50976071647639161454E-03

.26098881274143715531E-01 .26858768463458876363E+00

-.48859377638477896610E+00 -.56815384868504326121E+00

.98516857035195004588E+00 .54076925513274776147E+00

.11995831562685965732E-01 -.61238295572314275714E-02

.28209347657431960502E-02 -.15144311621171167165E-02

.57535356302051651224E-03

-.11494101675030059251E-01 -.11610625788720520257E+00

.16597770809257550439E+00 .40204746780277123008E+00

-.68175733992916076609E+00 -.18694149388874437001E+00

.82433576280849452100E+00 .34110291160584654824E+00

-.70602325918543672179E-02 .44072633136513286733E-02

-.16503474157193683599E-02

.37029388078082514646E+00 .67180940462189009388E+00

-.84780940184830764356E-02 .59331321868518463111E-02

-.18345110621146593771E-02 .59817306407676821764E-03

-.16221904397641732898E-03

-.19774009476717724354E+00 -.35989319683817344586E+00

.71527441002451253027E+00 .47782997129763499222E+00

-.52856073277453676298E-02 .32924147227717682346E-02

-.94291789530207134259E-03

.67917955907515231706E-01 .12974185507442576037E+00

-.31899787548219810107E+00 -.13632321028466456991E+00

.53012237138020978922E+00 .53379910667374919075E+00

.12747021311494910467E+00

.26312692875597260000E+00 .62364854793842017244E+00

.24480583194477717468E+00

0/

END

FIT

10S 3.63

10P 5.34

11P 3.33

10D 3.46

END

**Palladium (TZP, 4p frozen)**

BASIS

1S 30.000

2S 19.700

3S 9.400

4S 5.650

2P 20.650

3P 9.750

4P 4.250

3D 11.650

5S 2.500

5S 1.450

5S .850

4D 4.250

4D 2.200

4D 1.100

5P 1.450

END

CORE 4 3 1 0

1S 306.000

1S 76.400

1S 45.900

2S 53.800

2S 19.550

3S 10.150

3S 6.650

4S 6.150

4S 3.900

5S 2.500

5S 1.450

5S .850

2P 29.100

2P 18.750

3P 11.250

3P 8.100

4P 5.950

4P 3.950

4P 2.700

3D 17.550

3D 10.450

3D 6.900

4D 4.250

4D 2.200

4D 1.100

END

DESCRIPTION

.87837670917412149357E-02 .75113706571403537904E-01

.95002491048270998331E+00 -.24019588647992865665E-01

.76320066388707369018E-03 -.13104166910954994155E-02

.21384539670041675073E-02 -.15329870551205055772E-02

.43759754065334692617E-03 -.14606287637679434780E-03

.69731456593838191766E-04 -.24841351838398589852E-04

-.22855506239534677942E-02 -.16897886268759140793E+00

-.46379446058668399519E-02 -.28547817289875532243E+00

.11129602815708243835E+01 -.50121163925736997302E-02

.77255596569754956052E-02 -.43410195772192695582E-02

.71051223136973310889E-03 -.13023117772385469672E-03

.42333566740932710251E-04 -.12447993547943735806E-04

.51758845070735601777E-03 .16455554453997453535E+00

-.18748738990514152158E+00 .26540239127724135626E+00

-.64841462424046369950E+00 .10593259023339087754E+01

.15804286278020704404E+00 -.76373184310600361613E-01

.24959555150507267696E-01 -.97108611189647055933E-02

.49509126658509932870E-02 -.18130803650276755210E-02

-.16645341395328576645E-03 -.82487615873943848088E-01

.10434574896551979117E+00 -.13212896591161088500E+00

.29885253161658931198E+00 -.51481945967890485605E+00

-.38014216718353766034E+00 .10204823475768902696E+01

.41342651870078661602E+00 .11141192071675222464E-01

-.50867319416374977281E-02 .20110683677569741491E-02

.25485900773924602403E+00 .76629395288967150357E+00

.36210565479325453574E-02 -.45601898309684668620E-02

.40491567172454357229E-02 -.29866193044093647217E-02

.11077125501888344418E-02

-.86463446687351480646E-01 -.42368906373831527468E+00

.66308758251464960942E+00 .49468044828397245150E+00

-.95736588134956827900E-02 .59242758787109352919E-02

-.19624338000870261180E-02

.32251289206099681417E-01 .18051867756511449925E+00

-.29863621116777522291E+00 -.32464048367229819680E+00

.58943951524167403910E+00 .53024020292996354442E+00

.11447776052936886604E+00

.15269537995380963458E+00 .59293913376715590147E+00

.32341738519658219664E+00 -.79119693995344090622E-03

.46774224704778679090E-03 -.14315899957525985787E-03

0/

END

FIT

6S 11.25

8S 4.46

9S 3.21

9S 2.10

7P 10.21

8P 5.62

9P 3.19

9P 2.30

6D 11.37

7D 8.66

8D 3.05

7F 6.71

7F 4.20

END

**Polonium (TZP, 5d frozen)**

BASIS

1S 62.000

2S 39.250

3S 23.000

4S 13.450

5S 7.950

2P 41.500

3P 20.150

4P 13.250

5P 7.100

3D 24.200

4D 12.000

5D 4.400

4F 9.550

1S 206.600

6S 4.050

6S 2.700

6S 1.830

6P 3.100

6P 1.900

6P 1.170

END

CORE 5 4 3 1

1S 867.000

1S 206.600

1S 95.400

2S 110.600

2S 41.550

3S 52.850

3S 22.650

4S 13.800

4S 9.800

5S 8.150

5S 5.600

6S 4.050

6S 2.700

6S 1.830

2P 73.100

2P 39.450

3P 22.400

3P 16.300

4P 15.150

4P 10.350

5P 7.000

5P 4.600

6P 3.100

6P 1.900

6P 1.170

3D 30.300

3D 19.450

4D 13.500

4D 8.900

5D 6.450

5D 4.100

5D 2.600

4F 18.400

4F 10.650

4F 6.350

END

DESCRIPTION

.24755123166664647766E-01 .15739550201460497658E+00

.85173586876967632975E+00 .19539711535261988784E-01

-.44874134836587337150E-01 .39300092340217772369E-01

.13358074167058473988E-02 -.77762247425493930507E-03

.73061395633718790015E-03 -.39379607802113986408E-03

.15136070484812343107E-03 -.55945392191667577030E-04

.26030605300419352055E-04 -.82440170496164871838E-05

-.84119168842961000720E-02 -.62087463857602859241E-01

-.26793549585500386900E+00 -.84574861962238206958E-01

.10060454269901522029E+01 .86380671540242356943E-01

.86273928319725845786E-03 .52848560158539033618E-02

-.79273314678763185520E-02 .61733792202869541485E-02

-.32389446207354570702E-02 .15102624955403371257E-02

-.79692596601048653216E-03 .26907352339132191779E-03

.40952366266147049306E-02 .20177500410948092519E-01

.15337116062883929257E+00 -.39980241538661812767E-02

-.34215022271030476331E+00 -.34868488510040029915E+00

.11947094698936746582E+01 -.10228216251626039059E-02

.94504230530976086715E-03 .22135790338009531629E-02

-.23179221471384507669E-02 .14260843791750082207E-02

-.84077244097185283023E-03 .29832653810559633945E-03

-.21677877040343906945E-02 -.53989243986666884609E-02

-.93214487242327906391E-01 .25810443045972384507E-01

.91069409003793788671E-01 .29733724585442500299E+00

-.89237721379172174618E+00 .10975150893078777781E+01

.18368930413496617482E+00 -.20390676481322290992E-01

.12002902029215231985E-01 -.71162088014163462149E-02

.42382822032856594732E-02 -.15210712575395974287E-02

.10422256192294961606E-02 .16477747665946107774E-02

.46973625650344685845E-01 -.16648615889238070620E-01

-.27501842511389769375E-01 -.16185310784486697799E+00

.46555632390352635896E+00 -.72939596855558808119E+00

-.11769815732525769347E+00 .88110566873230444784E+00

.37102889912969100328E+00 .11400967878249733053E-01

-.62091692320200573066E-02 .25339207940135812826E-02

.14715337709997666771E+00 .87853415434518478122E+00

.13552109946855277894E-01 -.26964856575416643741E-01

.23591717295703581309E-01 -.10081511406278154813E-01

.43237140732309952557E-02 -.24402843891810866925E-02

.10559435383094212865E-02 -.53112416842172676616E-03

.19018074125298401367E-03

-.58517011376941138745E-01 -.51336023159797117277E+00

.97652296600103316582E+00 .25623927976233501491E+00

-.11080769033407016078E+00 .40313089414777986130E-01

-.16773578172106718559E-01 .94389685032537848364E-02

-.40967325634915507937E-02 .20674954959257478733E-02

-.74191354261627913500E-03

.26708833158055528267E-01 .27018676707410843596E+00

-.47188126465538293708E+00 -.64132029038254012576E+00

.10257064207601593875E+01 .55453786980560237918E+00

.14790014090549672560E-01 -.86269163424687201475E-02

.42022784461287097588E-02 -.22385608208845600951E-02

.82320915221767898464E-03

-.12088693542107094639E-01 -.12041359698090776942E+00

.16037959748692892648E+00 .46115765585948381711E+00

-.72650632886543076161E+00 -.21854129901118959922E+00

.82427940027711221749E+00 .34900246857874095419E+00

-.34379968928675641929E-02 .27712743116718452363E-02

-.97808411940122951325E-03

.36720699958864372325E+00 .67281167406821962285E+00

-.53521284218262790353E-02 .37673926740700957976E-02

-.52912215297869875322E-03 -.15129143442565729785E-03

.11820973558633443080E-03

-.19775739448114731944E+00 -.36666091936555644804E+00

.70704421983871901247E+00 .48569301910667100897E+00

-.40536358397894392547E-02 .28921164676199034339E-02

-.72628026481901019132E-03

.73318713072768396111E-01 .14289208653987270359E+00

-.34264439618550140976E+00 -.15996821069751471223E+00

.53712334110492154782E+00 .53039634057706686043E+00

.11709265288416906281E+00

.25042461156438156067E+00 .61165907688458831526E+00

.26067275344601109621E+00

0/

END

FIT

10S 5.23

11S 2.79

10P 4.69

11P 3.00

10D 5.07

END

**Platinum (TZP, 4f frozen)**

BASIS

1S 56.000

2S 36.100

3S 20.700

4S 12.200

2P 39.350

3P 17.450

4P 12.400

3D 21.750

4D 10.950

4F 7.650

1S 181.700

5S 6.800

5S 4.550

6S 3.100

6S 1.950

6S 1.240

5P 6.500

5P 4.400

5P 3.050

5D 4.950

5D 2.800

5D 1.550

6P 1.950

END

CORE 4 3 2 1

1S 751.000

1S 181.700

1S 86.300

2S 97.300

2S 37.600

3S 48.000

3S 20.400

4S 12.450

4S 8.800

5S 6.800

5S 4.550

6S 3.100

6S 1.950

6S 1.240

2P 64.600

2P 36.000

3P 20.500

3P 15.150

4P 13.300

4P 9.050

5P 5.850

5P 3.650

3D 27.550

3D 17.600

4D 12.000

4D 7.650

5D 4.950

5D 2.800

5D 1.550

4F 15.700

4F 8.600

4F 4.700

END

DESCRIPTION

.21068899084433066748E-01 .13623244672669554411E+00

.87786280146372830391E+00 .65713017829612503998E-02

-.25294263781606349945E-01 .22209870048660138031E-01

.92713577972005842276E-03 -.68174528981444337122E-03

.64135423455104613281E-03 -.35470979778570304068E-03

.16695190633586216313E-03 -.67004250133862554078E-04

.33964505150687408818E-04 -.11963365717704869197E-04

-.70957255010292508493E-02 -.53595509923510663286E-01

-.27828892207130534464E+00 -.86280968228148027799E-01

.99158955931287717789E+00 .10472365602303711218E+00

.19521071800875403838E-02 .26869996187096202404E-02

-.36656748902327701556E-02 .25728659161363301271E-02

-.13761337214597991075E-02 .59677362740115000826E-03

-.31235809577754214059E-03 .11151749074090949385E-03

.34555106696956866714E-02 .15304566044705123634E-01

.15857378097668459183E+00 -.89069566674249128074E-02

-.29946198921630656775E+00 -.38540419120778279805E+00

.11905699594325505064E+01 -.15749933833305606624E-02

.27104116720472522933E-02 -.46294107523038311911E-04

-.54877251788578828694E-03 .38865009086390254383E-03

-.23580541744366739798E-03 .89053264155762546991E-04

-.18122214112892959508E-02 -.26495708716963227262E-02

-.94594569432916664198E-01 .29968956154951039567E-01

.56140602545279312541E-01 .32085321762320484495E+00

-.87235971362640118976E+00 .10530766779723057525E+01

.21883724817194599721E+00 -.11342413445453333048E-01

.55136444364751084971E-02 -.28158091707192775370E-02

.16105145220333067294E-02 -.59977605057360487261E-03

.15235031438435656104E+00 .87182082290351237130E+00

.82241312726582926212E-02 -.14183936289227058389E-01

.11968244632943485228E-01 -.53451494008140117489E-02

.16906558781053272755E-02 -.62109561755518310003E-03

-.57861291746540699510E-01 -.51048550905193057492E+00

.97027501332862919892E+00 .22933775438465714425E+00

-.61816565455424299369E-01 .22966690170210593558E-01

-.69819375649730498062E-02 .25585732218276484480E-02

.25185225571116635207E-01 .26496904861368975048E+00

-.50252251033345196607E+00 -.46740593053067019946E+00

.92454516905356554091E+00 .52003316697636048360E+00

.87920895109763631120E-02 -.28586845893066353992E-02

.37671775197727463969E+00 .66399011202835467316E+00

-.43199227258642032903E-02 .36931565609444644456E-02

-.99721888912622557236E-03 .36215756144108909040E-03

-.11335990691722410957E-03

-.19857303260660083555E+00 -.34039825077491814298E+00

.71918539965637739986E+00 .46565328438990571946E+00

-.18252936975180335342E-02 .21734548167792891796E-02

-.74237078897228150464E-03

.29670095625897796587E+00 .62053682794323472560E+00

.23563838036219963978E+00

0/

END

FIT

9S 8.52

8P 8.63

9P 5.45

10P 4.58

10P 3.50

8D 9.14

9D 4.99

10D 3.91

9F 6.29

9F 4.05

END

**Radium (TZP, 5d frozen)**

BASIS

1S 66.000

2S 43.050

3S 24.800

4S 13.700

5S 9.250

2P 43.450

3P 22.000

4P 13.600

5P 8.150

3D 24.900

4D 13.350

5D 5.300

4F 10.700

1S 225.000

6S 4.400

6S 2.850

7S 2.100

7S 1.350

7S .890

6P 3.500

6P 2.100

7P 1.350

6D 2.500

6D 1.250

5F 2.000

END

CORE 5 4 3 1

1S 954.000

1S 225.000

1S 101.900

2S 121.900

2S 44.300

3S 56.350

3S 24.200

4S 14.800

4S 10.550

5S 9.250

5S 6.450

6S 4.400

6S 2.850

7S 2.088

7S 1.350

7S .890

2P 79.250

2P 41.800

3P 23.750

3P 17.200

4P 16.600

4P 11.350

5P 7.900

5P 5.350

6P 3.500

6P 2.100

3D 32.300

3D 20.750

4D 14.550

4D 9.800

5D 6.500

5D 4.050

4F 20.400

4F 12.050

4F 7.400

END

DESCRIPTION

.27551786885258694459E-01 .17288206455892543567E+00

.83157178859608082977E+00 .28832616494016301972E-01

-.53210862312668527618E-01 .46999662335970845972E-01

.13189996591067133878E-02 -.33402391448649469165E-03

.30714490213994686445E-04 .17571448528642055081E-03

-.13765501283107673238E-03 .70064719545238196953E-04

-.45918854846336231400E-04 .26177494639494050830E-04

-.13297865719866369051E-04 .48051463364650815526E-05

-.94124202956843844303E-02 -.69063525991318153174E-01

-.25723599439304517533E+00 -.84027497180800311827E-01

.10122301307209240484E+01 .76195416116201036827E-01

-.48619562174444702672E-03 .87413766430146297437E-02

-.13955440269491595218E-01 .10653379984515806700E-01

-.44257437126053881041E-02 .17481831985219386302E-02

-.10582847468593506706E-02 .58277158039753682896E-03

-.29122148531749884404E-03 .10453828211902531950E-03

.45797358418702104055E-02 .23758760911685165157E-01

.14886422420325898242E+00 -.36551306394208300105E-03

-.37233210183763409962E+00 -.31975201468133196414E+00

.11970361787652625818E+01 -.47167200784337453764E-02

.41446953519034155852E-02 .88002572574429744050E-03

-.18996392353182688355E-02 .11654684244210809785E-02

-.80498058232796824291E-03 .46988454699309280434E-03

-.24153528296646717671E-03 .87723050038709599117E-04

-.24400808335116540813E-02 -.72731748961227969327E-02

-.92287001156688336367E-01 .22728822642831693346E-01

.11781771880453274781E+00 .27667096876459629229E+00

-.90522663844541495504E+00 .11082065103928298022E+01

.19027861755920444375E+00 -.39301107173200913736E-01

.18540267964226798264E-01 -.91723330501629861400E-02

.60949284058157424462E-02 -.35218512576764790002E-02

.18063697105790293713E-02 -.65614813395397015607E-03

.12144598734750151718E-02 .26539614575840698475E-02

.48259680350171710317E-01 -.15350764512551346233E-01

-.43978512782717978091E-01 -.15578891699043465047E+00

.49040213542983773776E+00 -.73793070681865136073E+00

-.23885826469227072755E+00 .92705635550319254179E+00

.42653297515674648555E+00 .95573091787155874111E-02

-.58195964044443137739E-02 .37142440554522519573E-02

-.20205676244691508914E-02 .75489376057575642565E-03

.14441280975934958630E+00 .88275724401261190799E+00

.16860676922607856221E-01 -.37464962283522321163E-01

.33062497778408798943E-01 -.12856379551794265906E-01

.56601967433949343031E-02 -.29151252135774549483E-02

.94114986360310912052E-03 -.32056936590289591364E-03

-.59199493143722352329E-01 -.51463209002334431297E+00

.95407612990362677952E+00 .32168524775460344278E+00

-.16725414521440126747E+00 .53461934202631858359E-01

-.22536583872631302944E-01 .11505319527860949358E-01

-.37126388352432993130E-02 .12672995967036432535E-02

.27911357992663029387E-01 .27294958949251657554E+00

-.41160805454914184409E+00 -.84806847986775146797E+00

.11228552038726129503E+01 .60179907940235433106E+00

.19590470922918611779E-01 -.10142756550654233316E-01

.36686225746828137785E-02 -.13172408701615119769E-02

-.13149729046761572671E-01 -.12859342526529915496E+00

.14055863822665301544E+00 .59582475492681075124E+00

-.79216717294573268937E+00 -.32707124069857157478E+00

.83157112387622267313E+00 .38212677672663836193E+00

-.59717321004892917960E-02 .25304629055290148686E-02

.35477553783026832823E+00 .68468265566878239170E+00

-.58558350857464006381E-02 .40713914538595442905E-02

-.66782521156363860732E-03 .10905687520030347244E-03

-.19304653772490207309E+00 -.38801782937286610764E+00

.69381418982068610024E+00 .50282433188825659265E+00

-.15265138531417765839E-02 .12890525574528659356E-02

.77107362845514768046E-01 .17717173506523747895E+00

-.41331717403311912795E+00 -.12950997408384737342E+00

.77415321266942305201E+00 .35974345755274794723E+00

.21621802213349644584E+00 .61257174656860413631E+00

.28114001745561340018E+00

0/

END

FIT

11S 3.72

11P 5.05

12P 3.32

END

**Rubidium (TZP, 4p frozen)**

BASIS

1S 25.600

2S 15.250

3S 7.600

4S 3.550

2P 17.050

3P 7.300

4P 2.450

3D 6.080

5S 1.600

5S 0.950

5S 0.610

5P 0.950

4D 2.800

4D 1.400

END

CORE 4 3 1 0

1S 228.000

1S 56.100

1S 36.100

2S 43.100

2S 15.100

3S 7.750

3S 5.300

4S 3.750

4S 2.350

5S 1.600

5S .950

5S .610

2P 22.000

2P 14.300

3P 8.500

3P 5.600

4P 3.750

4P 2.400

4P 1.550

3D 12.200

3D 7.000

3D 4.200

END

DESCRIPTION

.61843450875096479119E-02 .37505284023757058665E-01

.10216421210946784459E+01 -.63620672973920391891E-01

-.25906856415952909077E-03 .11700736154573796843E-03

-.84141911140194400038E-04 .29819196955316903778E-04

-.12257425623444972705E-04 .44907126501006867933E-05

-.21071854741720857060E-05 .78635161697281250524E-06

-.13048993540086773965E-02 -.40712425566024423862E+00

.43352283191304369359E+00 -.49724817944096455502E+00

.10999955305176403186E+01 -.39071460063144465916E-02

.50639519929615893837E-02 -.24825301537591691788E-02

.12622069670754380508E-02 -.54867921809049916091E-03

.28424456122369301829E-03 -.11056479405906618834E-03

.12481683133926316904E-03 .36412667974747037203E+00

-.55393725541517346844E+00 .43071671465941530066E+00

-.59084536316813152101E+00 .96223613893721449131E+00

.19738765845041111868E+00 -.12746275982905298438E-01

.74822538870803539587E-02 -.40591497134208924177E-02

.23668580135584563369E-02 -.96531211612018992356E-03

-.13618315565755260045E-05 -.15316960726378053836E+00

.24092754637327487566E+00 -.18022029872601971756E+00

.22649581724883363631E+00 -.44266301666640717194E+00

-.68247509249615589955E-01 .77653259744113678487E+00

.36832002152514720095E+00 .41842051818385440909E-02

-.26672837448502508158E-02 .14009546016340791892E-02

.30182659709286741023E+00 .72110226861141690780E+00

.14507707355302548562E-02 -.11684000021394231814E-02

.12932591473695522703E-02 -.10395498786013406250E-02

.40103239780155076552E-03

-.10287403174632182845E+00 -.36598809619397804083E+00

.66505515343100962689E+00 .48348542652036036182E+00

-.66363873280053198387E-03 .57558422113085914475E-03

-.12704681238795244094E-04

.30083791380577044189E-01 .12202084115254246965E+00

-.25201982878854517489E+00 -.16814799552115541892E+00

.45791732668171969411E+00 .54887872917216218305E+00

.15113389384195527465E+00

.22140790030395846033E+00 .57355840496320209532E+00

.30684999270414348160E+00

0/

END

FIT

5S 9.64

6S 8.67

7S 5.96

7S 4.66

8S 4.17

8S 3.31

9S 1.89

9S 1.52

9S 1.22

5P 13.31

6P 10.44

6P 7.03

7P 5.55

7P 3.84

8P 3.05

8P 2.16

7D 4.23

8D 1.86

END

**Rhenium (TZP, 4f frozen)**

BASIS

1S 53.000

2S 33.450

3S 19.400

4S 11.950

2P 37.450

3P 16.750

4P 11.750

3D 20.250

4D 10.650

4F 6.600

1S 170.200

5S 6.200

5S 4.100

6S 2.950

6S 1.850

6S 1.170

5P 6.100

5P 4.150

5P 2.800

5D 4.300

5D 2.400

5D 1.310

6P 1.850

END

CORE 4 3 2 1

1S 699.000

1S 170.200

1S 82.000

2S 91.500

2S 35.750

3S 45.600

3S 19.300

4S 11.800

4S 8.300

5S 6.200

5S 4.100

6S 2.950

6S 1.850

6S 1.170

2P 60.500

2P 34.300

3P 19.550

3P 14.550

4P 12.450

4P 8.400

5P 5.350

5P 3.300

3D 26.200

3D 16.650

4D 11.250

4D 7.000

5D 4.300

5D 2.400

5D 1.310

4F 14.200

4F 7.500

4F 3.850

END

DESCRIPTION

.19409139107550345965E-01 .12670498782608075050E+00

.88914739389167340988E+00 .80848420117615943728E-03

-.14108698117454049337E-01 .12607105130824856551E-01

.48567325007989949363E-03 -.35497588880688870630E-03

.32384249615164713157E-03 -.18235448127934701917E-03

.10049589236711968434E-03 -.46162124983513037088E-04

.21348997558379138618E-04 -.73150042432621089342E-05

-.65059728308488025753E-02 -.49950693075592689230E-01

-.28209041240640886361E+00 -.87175728908453417598E-01

.98043005871498478676E+00 .11681356385479906013E+00

.35429055974459241693E-02 .77410564204240435177E-03

-.15399105335614712842E-02 .12618708132932246555E-02

-.81539798431540483174E-03 .40734869245081752791E-03

-.19621578492551791108E-03 .68371989959764133821E-04

.31637026011626535188E-02 .13352774296591898157E-01

.15970492142222122989E+00 -.93548982099297649240E-02

-.28681723968840872052E+00 -.39450834848764892993E+00

.11899170643541951975E+01 -.69107932991786212573E-02

.79223693519737629215E-02 -.33097816571050287307E-02

.13590655125656435874E-02 -.48731711582027993114E-03

.19211408067252357676E-03 -.60997068868078888548E-04

-.16517728798302795147E-02 -.15058807275840717897E-02

-.94589480175615817115E-01 .30680819439497814677E-01

.46168053896515723333E-01 .32519443260094843540E+00

-.86377963314127703676E+00 .10365891140416112481E+01

.23258152293815378964E+00 -.78267948016616309403E-02

.40802442972721914699E-02 -.23166045143938776389E-02

.12252892377813497972E-02 -.44676511670642106650E-03

.15667315362583916771E+00 .86670428958387846219E+00

.76812020914263775748E-02 -.12317034578108289680E-01

.99547209788724347335E-02 -.45458985263564580248E-02

.14402700736680078969E-02 -.53663004671470097232E-03

-.58325965466229978684E-01 -.50756389434965398788E+00

.96395106112806627774E+00 .22576993792791361093E+00

-.47441502847035348089E-01 .18339373603264267493E-01

-.56351454643225962551E-02 .21010536418316406483E-02

.24862901244022333341E-01 .26145698082128715489E+00

-.50627671133726270813E+00 -.41713047950115261786E+00

.90538842918230422185E+00 .49900990021300306587E+00

.68287963892113799635E-02 -.22127883221909759766E-02

.38297658859319050340E+00 .65967263860084346394E+00

-.59334223243982207874E-02 .43746098328102172789E-02

-.13601329169323323701E-02 .60256903583261786205E-03

-.20612466305726951103E-03

-.19929087246743679152E+00 -.32762418632056106160E+00

.73328297587963064963E+00 .45140415320492732754E+00

-.41513903134008465351E-02 .31335993731857751839E-02

-.11228472575390449455E-02

.33140229888841543637E+00 .61620732774884912075E+00

.22891860731243227067E+00

0/

END

FIT

9S 8.04

7P 12.81

8P 10.65

8P 7.88

9P 6.58

9P 4.95

10P 4.14

10P 3.16

8D 8.30

9D 6.42

10D 3.49

8F 13.00

8F 8.14

9F 5.78

9F 3.71

8G 6.60

9G 4.37

END

**Rhodium (TZP, 4p frozen)**

BASIS

1S 29.900

2S 19.300

3S 9.150

4S 5.500

2P 20.050

3P 9.550

4P 4.100

3D 11.000

5S 2.400

5S 1.450

5S .910

4D 4.250

4D 2.300

4D 1.220

5P 1.450

END

CORE 4 3 1 0

1S 297.000

1S 74.200

1S 44.800

2S 51.200

2S 19.100

3S 9.900

3S 6.650

4S 5.800

4S 3.750

5S 2.400

5S 1.450

5S .910

2P 27.950

2P 18.200

3P 11.000

3P 7.850

4P 5.650

4P 3.800

4P 2.650

3D 16.850

3D 10.100

3D 6.650

4D 4.250

4D 2.300

4D 1.220

END

DESCRIPTION

.84187631342328792666E-02 .78877629709428354055E-01

.94087500478000485327E+00 -.18083303188571757392E-01

.10082925796971464478E-02 -.15311690892557358080E-02

.21894469727769920453E-02 -.15035984151029605557E-02

.53957224088901705687E-03 -.19412857564493813832E-03

.10645292531965156596E-03 -.39656899348153682599E-04

-.22718150711754912276E-02 -.12966743627339932798E+00

-.87820087799117166338E-01 -.23878319224872227022E+00

.11169006170386195365E+01 -.46956531824061051306E-02

.85366489342758450354E-02 -.54196814832957341396E-02

.16864446952785869249E-02 -.53674019562952371681E-03

.27953600780371133270E-03 -.10194386869302771799E-03

.52601182114424412887E-03 .13079194147027173312E+00

-.11570586058313353517E+00 .22431857995945142514E+00

-.64986556326349886881E+00 .10496429686064130404E+01

.14997765703826221317E+00 -.54944751319123803779E-01

.22044468530360294967E-01 -.92402236072382307186E-02

.54064280304692621110E-02 -.20733555788155074925E-02

-.16622801408657848321E-03 -.66028229673071411887E-01

.69684759948033858645E-01 -.11196109038518797796E+00

.29801389948729345436E+00 -.52713967732623678852E+00

-.27350113917417723242E+00 .94607712975711544257E+00

.39003474911817920256E+00 .12727404623471333042E-01

-.72517548030513459198E-02 .29976426822696383701E-02

.27217196513029512372E+00 .74782013823696935617E+00

.80943451065293350183E-02 -.90090100679579426801E-02

.67029135655005825725E-02 -.49569758693176445286E-02

.18307678638403157567E-02

-.92469306397668524222E-01 -.41451390044765473730E+00

.64744239460003727427E+00 .51106304954433368071E+00

-.11507071682856338107E-01 .77761989170249600986E-02

-.26685824392565253224E-02

.34074174156208607289E-01 .17588974926777239949E+00

-.29633939126629760263E+00 -.30978833875805672493E+00

.59081704390892653134E+00 .51077712435367406130E+00

.11677212105662770902E+00

.16109297649991774493E+00 .57889101895542793841E+00

.33157203854174066571E+00 -.15045141165109122278E-02

.99474951093724548780E-03 -.33154313258953164610E-03

0/

END

FIT

5S 12.85

8S 4.71

9S 2.76

9S 2.24

8P 5.72

8P 4.06

9P 3.26

7D 4.36

8D 3.22

7G 5.43

END

**Radon (TZP, 5d frozen)**

BASIS

1S 64.000

2S 40.250

3S 23.700

4S 14.000

5S 8.250

2P 43.650

3P 20.050

4P 13.900

5P 7.400

3D 24.900

4D 12.500

5D 4.850

4F 10.150

1S 215.400

6S 4.500

6S 3.000

6S 2.000

6P 3.450

6P 2.150

6P 1.350

END

CORE 5 4 3 1

1S 907.000

1S 215.400

1S 98.600

2S 115.900

2S 42.900

3S 54.600

3S 23.400

4S 14.300

4S 10.200

5S 8.650

5S 6.000

6S 4.500

6S 3.000

6S 2.000

2P 76.100

2P 40.650

3P 23.050

3P 16.700

4P 15.850

4P 10.850

5P 7.400

5P 4.950

6P 3.450

6P 2.150

6P 1.350

3D 31.300

3D 20.100

4D 14.000

4D 9.300

5D 6.800

5D 4.450

5D 2.950

4F 19.500

4F 11.350

4F 6.850

END

DESCRIPTION

.26206033955796521895E-01 .16468338296469661741E+00

.84280431505994202190E+00 .23146308449480484937E-01

-.48450549792610271560E-01 .42584549251624720378E-01

.13836153976652762910E-02 -.70750827301082686634E-03

.60956357896563945990E-03 -.27927434013746467185E-03

.75558297072709270639E-04 -.16060860619278989164E-04

.29650798854706419775E-05 -.26751171651271107682E-06

-.89298052008614306474E-02 -.65309702123724611145E-01

-.26338192419804168365E+00 -.83874193320127965467E-01

.10102450341524855659E+01 .80455473354713008538E-01

-.10608405013387923533E-03 .75620205842415804884E-02

-.11615228161340338670E-01 .92778979692095114862E-02

-.49093924411961241752E-02 .22547884097952426360E-02

-.10452012521805457024E-02 .32831542947578667025E-03

.43451935386850685725E-02 .21887846299774103842E-01

.15131328609504712812E+00 -.23020593404628380066E-02

-.35722961071393249943E+00 -.33391978659272275598E+00

.11966725643922748734E+01 -.64496492364612568321E-02

.64068166364619613326E-02 -.74635998662760225011E-03

-.13692667052781198195E-02 .11263459866446097132E-02

-.64827382746592758338E-03 .22311845754065733971E-03

-.23075437478406206002E-02 -.62979645560362323281E-02

-.92812385156888554949E-01 .24393693612043389207E-01

.10373652796164872791E+00 .28739740994430723608E+00

-.89948712692672005975E+00 .11030938977332067630E+01

.18654149751164775251E+00 -.30139742243277090061E-01

.18766751645701396783E-01 -.10735699345408600616E-01

.56372996562154666436E-02 -.18888731750821116835E-02

.11296091647389522490E-02 .21188830944733039706E-02

.47688917073145632719E-01 -.16126373277101375969E-01

-.35058873666651876877E-01 -.15940310862427392591E+00

.47863486131652954736E+00 -.73759501189010467925E+00

-.16508848894579541966E+00 .90580768617403395471E+00

.38338497654996478436E+00 .14522368539169537918E-01

-.73959050090514569936E-02 .28485821884696766078E-02

.14551081925391001759E+00 .88000774096407319291E+00

.20460419961551772755E-01 -.40426790523746633510E-01

.33959956760202678061E-01 -.13302601636632757631E-01

.59400541244136462193E-02 -.35419878681077570336E-02

.15721359049925324146E-02 -.76833617573747394099E-03

.26949198818843186562E-03

-.58745454462988719313E-01 -.51355605831037043352E+00

.96850486662480061995E+00 .28497691434653138476E+00

-.14009662400075267863E+00 .48561713433578339305E-01

-.21373862409957374020E-01 .12761771070580634965E-01

-.56944511349768375461E-02 .27960372273734854058E-02

-.98356376327614759673E-03

.27232627960234262465E-01 .27142731603904707294E+00

-.44779061295121447284E+00 -.73551181838227897014E+00

.10734357639851306310E+01 .57493737189961735545E+00

.19681524197947750793E-01 -.12059409690358303610E-01

.59638193077364660619E-02 -.30929505754781162416E-02

.11168927852130403934E-02

-.12620049046110049309E-01 -.12439166743876850507E+00

.15044479145270980847E+00 .53265787095246963645E+00

-.77198160219547962146E+00 -.25917038945571280184E+00

.83374718381844059589E+00 .35336313446462636056E+00

-.49886983913000667451E-02 .33260422202386103092E-02

-.11390708002303414660E-02

.36065166322781855301E+00 .67905772619080340124E+00

-.55319573127329806778E-02 .38251834812488018887E-02

-.36067234651854755819E-03 -.35568518039360067211E-03

.21478254116602757623E-03

-.19537067290443133061E+00 -.37724281485750538190E+00

.70632320747774468561E+00 .49060389447379232353E+00

-.66775471538889075163E-02 .38381521379522139450E-02

-.97921074697353239699E-03

.76787607286104883619E-01 .15658230822873547838E+00

-.36673617820294784897E+00 -.17593416162421496307E+00

.56768627949963623713E+00 .51153616021806780356E+00

.10219927979945092611E+00

.22936098127838119853E+00 .61966802119243757918E+00

.26702987938744582319E+00

0/

END

FIT

11S 4.54

9P 10.92

9P 8.18

10P 6.83

10P 5.19

11P 3.35

10D 3.85

10F 6.49

10F 4.30

9G 5.00

END

**Ruthenium (TZP, 4p frozen)**

BASIS

1S 30.700

2S 18.750

3S 8.800

4S 5.350

2P 19.650

3P 9.200

4P 3.900

3D 10.700

5S 2.300

5S 1.400

5S .890

4D 4.000

4D 2.150

4D 1.140

5P 1.400

END

CORE 4 3 1 0

1S 288.000

1S 71.900

1S 43.700

2S 51.900

2S 18.550

3S 9.550

3S 6.100

4S 5.600

4S 3.600

5S 2.300

5S 1.400

5S .890

2P 27.400

2P 17.750

3P 10.700

3P 7.600

4P 5.500

4P 3.650

4P 2.500

3D 16.400

3D 9.750

3D 6.350

4D 4.000

4D 2.150

4D 1.140

END

DESCRIPTION

.81628045191196405700E-02 .65511331040644174561E-01

.96752121902923648999E+00 -.33287855528730155019E-01

.54801028218922753082E-03 -.93531299169345564516E-03

.15594006837649800317E-02 -.11927679225130864964E-02

.38350962511160855773E-03 -.14100250521783226697E-03

.79847646747182077806E-04 -.30076142208576838464E-04

-.19879605682004038159E-02 -.22091589999233252062E+00

.10081548223461501979E+00 -.34269351534052694408E+00

.11082298761266968778E+01 .36041485789828884862E-03

-.42611333845437779615E-03 .13721362886265678870E-02

-.93744586184709756144E-03 .46580577647732537925E-03

-.28998318867731005463E-03 .11330101050400241734E-03

.38463488558264087407E-03 .20690493660794123665E+00

-.27323887186188688947E+00 .30886643696549209714E+00

-.63427064151208700782E+00 .10640986416851387908E+01

.13854238219612527439E+00 -.62888792034719906421E-01

.22099796450552534194E-01 -.95898116394034678411E-02

.58144559363654865075E-02 -.22578752346128377372E-02

-.10556212551172590734E-03 -.10176776065683720840E+00

.14433512827371791643E+00 -.15097377974631961961E+00

.28647587039701366551E+00 -.52956099502619191632E+00

-.28600266459733830748E+00 .96782697305250464659E+00

.39540136824229693557E+00 .11267846776772890566E-01

-.67561714227173099920E-02 .28589114786774305968E-02

.26598078720827755816E+00 .75484327798831007961E+00

.49043803100446616700E-02 -.54365304714746008619E-02

.44065233837354287130E-02 -.32415587411564208108E-02

.12038300108050772365E-02

-.89977274167541504424E-01 -.41294889814911928116E+00

.64299386958141968584E+00 .51228169161061087866E+00

-.69718371692652934477E-02 .48891228075160653860E-02

-.16328764075117180657E-02

.33057524558005924042E-01 .17171612927275839433E+00

-.28570261239647959384E+00 -.31006133507928312909E+00

.56220584356085134825E+00 .54868261684615082796E+00

.10520332677497834517E+00

.16168245636351466499E+00 .58355364195709080732E+00

.32869681881530038448E+00 -.12274582475157462449E-02

.78921791986503694091E-03 -.25634332754835651574E-03

0/

END

FIT

7S 6.45

9P 3.17

9P 2.29

7D 6.59

8D 3.09

8D 2.03

7F 5.31

7G 2.28

END

**Sulphur (TZP, 2p frozen)**

BASIS

1S 13.45

2S 5.15

2P 6.15

3S 1.50

3S 2.25

3S 3.40

3P 1.00

3P 1.60

3P 2.45

3D 2.10

END

CORE 2 1 0 0

1S 14.35

1S 19.40

2S 4.25

2S 5.90

3S 2.25

3S 1.50

3S 3.45

2P 4.55

2P 8.05

3P 1.00

3P 1.60

3P 2.65

END

DESCRIPTION

.73529306698194618619E+00 .26918403901932036471E+00

-.27316307014589220198E-01 .21255050570878272764E-01

-.82006225872409254513E-02 .25571076288459573811E-02

.15797464667041488662E-01

-.39006810149841486535E+00 .46903228966606098915E-01

.27733497612529078857E+00 .82281442122930470440E+00

.18897614191974408671E-01 -.68050453949235166495E-02

-.30270067863224597166E-01

.62756541595009562862E+00 .41717140344133479823E+00

.19999637007486600546E-02 -.41399164788908562190E-02

.56663367140738166855E-02

0/

END

FIT

4S 4.48000000000000

5S 3.62000000000000

5S 2.76000000000000

4P 9.37000000000000

5P 4.44000000000000

5P 2.50000000000000

4D 9.16000000000000

5D 8.03000000000000

5D 4.38000000000000

5D 2.30000000000000

4F 2.90000000000000

END

**Antimony (TZP, 4d frozen)**

BASIS

1S 33.200

2S 22.450

3S 10.200

4S 6.850

2P 23.650

3P 10.900

4P 6.300

3D 12.800

4D 4.050

5S 3.400

5S 2.200

5S 1.470

5P 2.650

5P 1.600

5P .990

END

CORE 4 3 2 0

1S 363.000

1S 90.500

1S 51.600

2S 59.150

2S 22.150

3S 11.600

3S 7.500

4S 7.800

4S 5.000

5S 3.400

5S 2.200

5S 1.470

2P 33.300

2P 21.300

3P 12.650

3P 9.400

4P 6.500

4P 4.200

5P 2.650

5P 1.600

5P .990

3D 20.450

3D 12.350

3D 8.400

4D 6.150

4D 3.850

4D 2.450

END

DESCRIPTION

.10041679477884108557E-01 .82723508009450344436E-01

.93535234504576891812E+00 -.14622239938678066248E-01

.10394040599209788348E-02 -.25211173898242035356E-02

.58289474622410381022E-02 -.45180156867723570910E-02

.75622802721567769328E-03 -.31501688521870117778E-03

.18045093623345970441E-03 -.63509272376236140085E-04

-.28121205709528821794E-02 -.10295877565280577015E+00

-.13641642435195153182E+00 -.21736788326473860389E+00

.11166600493182035070E+01 .44352278470797595541E-02

-.11583313596360254177E-01 .10873554914864748747E-01

-.29778247732429722651E-02 .14952021058889655106E-02

-.92106585544361816841E-03 .33507078246413442204E-03

.81385760098984455749E-03 .98518738698823341005E-01

-.56448683320493429905E-01 .20102422664814725839E+00

-.66865923456282982951E+00 .10079478356231155178E+01

.38954207777407107161E+00 -.29209281033276873751E+00

.61355332489511783811E-01 -.29370398247616620602E-01

.17912847992095054517E-01 -.65130723248107928042E-02

-.32539348614254038749E-03 -.50738253048841816528E-01

.38589663779678803901E-01 -.10319488278555649652E+00

.32347357183692182492E+00 -.38618272569689016116E+00

-.10881392085017078664E+01 .15025899260472836971E+01

.50283001297996954282E+00 .25052647707267922117E-01

-.15099354454197601999E-01 .60192965047308321508E-02

.23547380398835132675E+00 .78412257424857034760E+00

.75927699029587174262E-02 -.74423697321216401163E-02

.43146274248509960958E-02 -.25722236991068676663E-02

.11223391061181038630E-02 -.66049804774943161011E-03

.24919498986404977660E-03

-.80234610658443950015E-01 -.44468398542201947876E+00

.70547835932169733475E+00 .45353738151385403210E+00

-.95410165418864031905E-02 .52871061037529825180E-02

-.22093249726890303725E-02 .12957942435820586722E-02

-.49000898197921072957E-03

.29918329243999338546E-01 .20830227888153587945E+00

-.37660879983345862954E+00 -.28189636949405627586E+00

.81478520853479330377E+00 .39638492082718562726E+00

.33138102219620996745E-02 -.13850313258068518759E-02

.62989493947668132902E-03

.13140276860755070731E+00 .58603863072087691855E+00

.34263935778569321045E+00 -.80951177854364573909E-03

.43564673123786733190E-03 -.38128892509193685050E-04

-.48683342809966174969E-01 -.26286687767690758433E+00

-.76011506100331874269E-01 .50027215916389700112E+00

.51154954818477205158E+00 .14254969523527361019E+00

0/

END

FIT

8S 3.89

8P 4.05

9P 3.32

8D 5.26

9D 2.82

8F 3.44

END

**Scandium (TZP, 3p frozen)**

BASIS

1S 12.80

2S 8.45

3S 3.45

2P 7.81

3P 2.92

3D 0.90

3D 2.00

3D 4.40

4S 0.75

4S 1.15

4S 1.90

4P 1.14

END

CORE 3 2 0 0

1S 25.20

1S 19.15

2S 7.95

2S 5.85

3S 4.30

3S 2.75

4S 0.78

4S 1.16

4S 1.80

2P 11.65

2P 6.95

3P 3.75

3P 2.25

END

DESCRIPTION

.29029417110936545088E+00 .71189450074329496676E+00

.31817742215137397033E-01 -.36664995145194473336E-01

.19055838152086793102E-01 -.10742448749755439952E-01

.12976227389653611360E-02 -.35496901979854317451E-02

.52594293316297526658E-02

.71571022659030147928E-01 -.44110458654381412558E+00

.95590312306231761674E+00 .14985293194116627857E+00

-.34823080864714403559E-01 .23414742759629472713E-01

-.34954970532873872770E-02 .93110402798215301245E-02

-.13089348058523055107E-01

-.35995677594200262139E-01 .17626709348520433474E+00

-.26658225442846072983E+00 -.34778676748884207459E+00

.77685319655497886426E+00 .47684573393233986582E+00

.36184469227245330547E-02 -.83123113498474836269E-02

.10052537237519063723E-01

.33028286323102379329E+00 .70453631072025724169E+00

-.57154413972497909290E-03 .11988034575525989122E-02

-.11165369578054037958E+00 -.27293452257864275756E+00

.71020199072587253397E+00 .41337158860797146298E+00

0/

END

FIT

6S 8.16

6S 5.93

7S 3.66

7S 2.72

7S 1.50

5P 7.09

6P 2.91

7P 2.04

5D 8.34

6D 5.66

7D 2.28

5G 3.89

5G 2.00

END

**Selenium (TZP, 3d frozen)**

BASIS

1S 21.45

2S 13.95

3S 6.60

2P 15.60

3P 6.45

3D 4.95

4S 1.45

4S 2.20

4S 3.40

4P 1.10

4P 1.80

4P 2.95

4D 1.8

END

CORE 3 2 1 0

1S 31.5500

1S 38.9000

2S 13.6000

2S 7.5000

3S 8.5000

3S 5.4000

4S 1.5000

4S 2.2000

4S 3.4000

2P 18.7000

2P 12.6000

3P 7.5500

3P 4.7500

4P 1.8000

4P 1.1000

4P 3.0000

3D 3.2000

3D 5.7000

3D 10.4000

END

DESCRIPTION

.47621839545565691765E+00 .51390733569556334981E+00

.14102592381224429707E+00 -.36373612394090493183E+00

.29086188665408013732E+00 -.32440472560848823691E-01

.41111121215606817592E-02 -.10947001967567711322E-01

.15348067332375493407E-01

-.49711759519625003945E+00 .93628958163318079522E-01

.10755615463537115950E+01 .11759555620259402875E+00

-.12675673744316465208E+00 .34971235012339403625E-01

-.53307200386087357294E-02 .13843056878828549280E-01

-.18484157654147778671E-01

.22456792983553050536E+00 -.57412050011810995820E-01

-.25907075531704853999E+00 -.11045068501117989435E+01

.14635459326533388769E+01 .55901738211713802862E+00

.51486059601485523532E-02 -.11746435376783211785E-01

.12942937786432406888E-01

.40655956262393450062E+00 .61115917352927318085E+00

.15674754457493278448E-01 -.12260240647804798025E-01

-.42890634496405783196E-02 .16087444294545017506E-02

.66431333805485170280E-02

-.14294140960023393738E+00 -.30633827662472984388E+00

.67649441190379877575E+00 .47262634973882949874E+00

.42559272865090166590E-02 -.14913261547999205614E-02

-.61843485601132596949E-02

.28434005446902371750E+00 .57780018399529409212E+00

.26291132190274224545E+00

0/

END

FIT

5S 10.9900000000000

6S 13.4500000000000

7S 3.30000000000000

6P 4.29000000000000

6P 4.87999999999991

7P 3.65000000000000

7P 2.47000000000000

6D 4.59000000000000

7D 2.96000000000000

7F 3.82000000000000

7G 3.95000000000000

END

**Silicon (TZP, 2p frozen)**

BASIS

1S 11.90

2S 4.50

2P 5.15

3S 1.20

3S 1.85

3S 2.85

3P 0.75

3P 1.20

3P 1.85

3D 1.75

END

CORE 2 1 0 0

1S 12.4500

1S 17.0500

2S 3.6000

2S 5.1500

3S 1.8500

3S 1.2000

3S 2.9500

2P 3.6500

2P 6.8500

3P 1.2000

3P 0.7500

3P 2.0000

END

DESCRIPTION

.74132093841004376156E+00 .26411308317587584016E+00

-.18823774413067550365E-01 .14634821984215454910E-01

-.52174506175092531454E-02 .16702118882283090509E-02

.10907496520984735419E-01

-.36650294003799654963E+00 .37081275742819150287E-01

.36137679162642760700E+00 .73511228779796289334E+00

.11624844777689234687E-01 -.42905376638512167028E-02

-.21742551312019638143E-01

.62828360476611955221E+00 .42795391550405398329E+00

.21991761834486583428E-02 -.80730955964365919229E-03

.72213139818808015288E-04

0/

END

FIT

4S 2.03000000000000

4S 11.0100000000000

4S 1.03000000000000

5S 2.41000000000000

5S 2.29000000000000

5P 5.50000000000000

5P 3.00000000000000

5P 1.89000000000000

3D 4.24000000000000

4D 7.59000000000000

4D 4.88000000000000

4F 2.57000000000000

END

**Tin (TZP, 4d frozen)**

BASIS

1S 32.400

2S 21.950

3S 10.050

4S 6.600

2P 23.300

3P 10.550

4P 6.150

3D 12.500

4D 3.750

5S 3.200

5S 2.050

5S 1.360

5P 2.450

5P 1.450

5P .880

END

CORE 4 3 2 0

1S 357.000

1S 89.000

1S 50.500

2S 56.800

2S 21.650

3S 11.300

3S 6.950

4S 7.400

4S 4.750

5S 3.200

5S 2.050

5S 1.360

2P 32.400

2P 20.750

3P 12.350

3P 9.150

4P 6.250

4P 4.000

5P 2.450

5P 1.450

5P .880

3D 19.900

3D 12.000

3D 8.100

4D 5.800

4D 3.550

4D 2.200

END

DESCRIPTION

.94970707936099605512E-02 .80346213927217871587E-01

.93629697727732252854E+00 -.13089263570928192162E-01

.12306609703521612098E-02 -.25492232699709624529E-02

.65355658276360197381E-02 -.52996457795536355828E-02

.75853580624540806968E-03 -.31424608254206932989E-03

.18020478288857316794E-03 -.64339935557231926625E-04

-.26993021742826332418E-02 -.84600153412044784562E-01

-.17476920450505958882E+00 -.19621017614113051009E+00

.11196138004732365534E+01 -.23422889082471949965E-02

.68991862060580637014E-02 -.43903125810590137856E-02

-.19869678513841391521E-03 .25517902611319484910E-03

-.18981104348721850820E-03 .74986579621537916875E-04

.77772693264365909318E-03 .82406373758493287052E-01

-.22641236073422454228E-01 .18185653473775426070E+00

-.66951710285459054006E+00 .10405494469233991239E+01

.33676780866501265788E+00 -.25820696077306098948E+00

.46020300266255170785E-01 -.22308998880045959740E-01

.13694792706011463604E-01 -.50588784092171455720E-02

-.30417997164012818088E-03 -.42486302172403948851E-01

.21859724039666215273E-01 -.93187190444953868496E-01

.32152204270811679532E+00 -.44328643991855587458E+00

-.10020321225409143384E+01 .15029518971950479589E+01

.48151949010602418388E+00 .19532637077167798456E-01

-.11857850221867747773E-01 .48356865810467017028E-02

.24140617691145571655E+00 .77949666553787677614E+00

.34694031906317675598E-02 -.38367704238847946500E-02

.26804053088441007549E-02 -.16355902990730700457E-02

.69876110668503309525E-03 -.41306976971155725305E-03

.15843890517474424435E-03

-.82601095524164960815E-01 -.44002287720230554013E+00

.70247556944007594204E+00 .45459849481057751364E+00

-.57214743073063321216E-02 .32650116545863247693E-02

-.13082554575176460858E-02 .76629913676523619698E-03

-.29388727208990545695E-03

.30342139006512374583E-01 .20383614572545727395E+00

-.37505298712670981365E+00 -.26529938579430001333E+00

.80919365386132269258E+00 .39238505385412353332E+00

.37713209615086130608E-02 -.13596085283156713729E-02

.57721781230098980452E-03

.13368284278700251488E+00 .58572513226009048637E+00

.34293698742167399995E+00 -.19188273526630941594E-02

.95964343618911351549E-03 -.22810735373671354878E-03

-.47265565934455890107E-01 -.25728899928379128648E+00

-.61337933970587787957E-01 .51116997262419816561E+00

.50065878667039964700E+00 .14284926587970067646E+00

0/

END

FIT

9S 3.19

8P 3.85

9P 3.15

9P 2.33

8D 3.27

9D 1.76

END

**Strontium (TZP, 4p frozen)**

BASIS

1S 24.450

2S 16.150

3S 7.750

4S 3.800

2P 17.400

3P 7.600

4P 2.750

3D 6.460

5S 1.800

5S 1.150

5S .760

5P 1.150

4D 3.000

4D 1.500

END

CORE 4 3 1 0

1S 227.000

1S 56.800

1S 37.100

2S 43.900

2S 15.600

3S 8.050

3S 5.600

4S 4.000

4S 2.600

5S 1.800

5S 1.150

5S .760

2P 22.800

2P 14.800

3P 8.900

3P 5.900

4P 4.000

4P 2.650

4P 1.800

3D 13.050

3D 7.450

3D 4.500

END

DESCRIPTION

.69062883632074953399E-02 .42086140648375874873E-01

.10160903647891488255E+01 -.63147883582113706336E-01

.28901854089001078279E-04 -.31953739466358696769E-03

.39372196985254656002E-03 -.27250280419570806038E-03

.17911447375486174967E-03 -.94149572197224873317E-04

.55539543848461027918E-04 -.20191186615569077555E-04

-.15440298858212853951E-02 -.40695595689294578046E+00

.41485021881388350895E+00 -.48030543036647643795E+00

.11035666724731263422E+01 -.43600949433435605071E-02

.59609424931958299859E-02 -.32111435271521262744E-02

.17919448553802969342E-02 -.83504694129979710124E-03

.46523732724727692939E-03 -.16473266714942186913E-03

.17013842954152920677E-03 .38430838536881767320E+00

-.57480573214914199021E+00 .43662621932157674731E+00

-.60052515184830612682E+00 .95325928912305146756E+00

.21149146231214527303E+00 -.20645607326961086525E-01

.14051141640033515401E-01 -.82121478772659208673E-02

.51368980086182319811E-02 -.19242739541282876618E-02

-.11254004074014144085E-04 -.16937913898528159096E+00

.26207450556180483803E+00 -.19129620287467274942E+00

.24005171175343040235E+00 -.45195796216960720804E+00

-.99118004915838608948E-01 .79637248528105475387E+00

.35631515268028846055E+00 .17226920541615987903E-01

-.10980798132340582884E-01 .45400183206968118196E-02

.29318529605172927166E+00 .72947071455113776661E+00

.14922680488725663037E-02 -.12879233887506959765E-02

.16306498751937422050E-02 -.14335243525988459936E-02

.56706067554836332451E-03

-.98346881137898994885E-01 -.37795321120430303008E+00

.64357810199231046955E+00 .51100930182353265785E+00

-.79945644609704276384E-02 .56358036687386989586E-02

-.18208388194563679668E-02

.30743115797380426990E-01 .13469805907424009517E+00

-.25921910579496665328E+00 -.20186710496128743020E+00

.47959342409377070382E+00 .53441127668025933950E+00

.14531590812776756794E+00

.19747957452225098351E+00 .59167361021622788542E+00

.30702450959035876288E+00

0/

END

FIT

8S 3.92

7P 6.27

7P 4.41

8P 3.56

END

**Tantalum (TZP, 4f frozen)**

BASIS

1S 51.000

2S 32.550

3S 18.700

4S 11.500

2P 36.400

3P 16.200

4P 11.350

3D 19.300

4D 10.400

4F 5.750

1S 162.500

5S 5.850

5S 3.800

6S 2.750

6S 1.750

6S 1.120

5P 5.750

5P 3.850

5P 2.600

5D 3.850

5D 2.050

5D 1.080

6P 1.750

END

CORE 4 3 2 1

1S 665.000

1S 162.500

1S 79.200

2S 88.100

2S 34.500

3S 44.100

3S 18.550

4S 11.400

4S 8.000

5S 5.850

5S 3.800

6S 2.750

6S 1.750

6S 1.120

2P 57.900

2P 33.200

3P 18.900

3P 14.150

4P 11.850

4P 8.000

5P 5.000

5P 3.050

3D 25.350

3D 16.050

4D 10.750

4D 6.600

5D 3.850

5D 2.050

5D 1.080

4F 12.950

4F 6.600

4F 3.200

END

DESCRIPTION

.18415978774280472585E-01 .12127230653107828884E+00

.89530442507595353607E+00 -.24008372145526025304E-02

-.73534343424149963692E-02 .67995768912316274443E-02

.25927558788616791516E-03 -.21240093644039358448E-03

.19526188351837072809E-03 -.11045728341788477232E-03

.64950509804932894664E-04 -.33221322059664546865E-04

.16238889896599141398E-04 -.55738309058624265898E-05

-.61508673980603242523E-02 -.48266838162483380326E-01

-.28292342633714745448E+00 -.88541937851508614732E-01

.97703541055992382258E+00 .12106354344291700431E+00

.33004417429020140896E-02 .93134283631672392344E-03

-.16094031736462463891E-02 .12352819858094058608E-02

-.81942671313069202852E-03 .44472725290594581878E-03

-.22370158674137893091E-03 .77733584050829884890E-04

.29858667961554859367E-02 .12251953527657457113E-01

.15995025452418182299E+00 -.90947373291635075221E-02

-.28116461928201014198E+00 -.39633130759159518330E+00

.11906693841861468730E+01 -.16510353148556405206E-01

.15970168019090066197E-01 -.77357830568122246118E-02

.41005105139923902352E-02 -.19734879099291540577E-02

.93552397186374882786E-03 -.31703200635862594306E-03

-.15544598568872189555E-02 -.78137329004844861217E-03

-.94568958358029669986E-01 .30827476561350448253E-01

.41093533671026770404E-01 .32578631745857478119E+00

-.85959834107709187645E+00 .10225377290175403644E+01

.24797471881275992556E+00 -.63883353411092521495E-02

.33068993803456181446E-02 -.19985753052312780174E-02

.10960968491141474465E-02 -.39825100690278169690E-03

.15931584764778433660E+00 .86310634076634507039E+00

.91534939503924977328E-02 -.13037826319837617084E-01

.96257861899865584943E-02 -.43657767114489752849E-02

.13244018772700109823E-02 -.49815607999687688563E-03

-.58571365892319343649E-01 -.50505326788924675974E+00

.96300339648389399194E+00 .21890817127136760756E+00

-.37218192094993288177E-01 .15193858457005438023E-01

-.45949498995136355342E-02 .17434646974093432909E-02

.24508657410961011414E-01 .25927926669948653249E+00

-.51719134654327869605E+00 -.36922611623219525612E+00

.88897305972995455114E+00 .48077992099177496721E+00

.73878769651245833686E-02 -.23748220459143547408E-02

.38412771088164904265E+00 .65924483097372232887E+00

-.60032988220938676011E-02 .43432259264137799276E-02

-.12983669020505347620E-02 .58086651296945942223E-03

-.20586661307593428182E-03

-.19849420386855892717E+00 -.31974454993125095426E+00

.74166360506585971013E+00 .44075666156140980156E+00

-.34101021600821293349E-02 .26476929018628275747E-02

-.98616709519954814760E-03

.37483749704908936318E+00 .60560600624943061732E+00

.21865665201499029369E+00

0/

END

FIT

10S 4.09

11S 3.76

8P 7.18

9P 4.47

10P 2.83

9D 4.03

10D 3.12

END

**Technetium (TZP, 4p frozen)**

BASIS

1S 30.200

2S 18.250

3S 8.600

4S 5.100

2P 19.100

3P 9.000

4P 3.750

3D 10.350

5S 2.250

5S 1.400

5S .900

4D 3.750

4D 2.000

4D 1.060

5P 1.350

END

CORE 4 3 1 0

1S 278.000

1S 69.500

1S 42.600

2S 50.700

2S 18.050

3S 9.300

3S 6.150

4S 5.350

4S 3.450

5S 2.250

5S 1.400

5S .900

2P 26.350

2P 17.200

3P 10.400

3P 7.300

4P 5.250

4P 3.500

4P 2.400

3D 15.700

3D 9.400

3D 6.100

4D 3.750

4D 2.000

4D 1.060

END

DESCRIPTION

.79145047516946693406E-02 .61202888854256155826E-01

.97570466349626838731E+00 -.37907941910772263716E-01

.47787515841607740340E-03 -.86958946980316309972E-03

.12995641857762437951E-02 -.94788218793526797697E-03

.36904172984071517260E-03 -.14923325084138000149E-03

.86008472293281625857E-04 -.31601822840011504644E-04

-.18846657191719823777E-02 -.24368168089165834211E+00

.14385951728560175678E+00 -.36420031641912625764E+00

.11069572222663011107E+01 .36631594949465198915E-03

-.31713446299915258380E-03 .12946183065552653146E-02

-.98227535239633033404E-03 .52798172836190492702E-03

-.33418289340196707720E-03 .12759108591916978773E-03

.33525618784804156860E-03 .22698832954900086123E+00

-.31111054037620206048E+00 .32665843383733655925E+00

-.62855394439563638809E+00 .10430484119501870488E+01

.15066633671799051220E+00 -.55251341791909991397E-01

.23452098158697060049E-01 -.10985553800973327926E-01

.67524448361199786162E-02 -.25582528698504304089E-02

-.85677833893304004235E-04 -.11005744044555171302E+00

.16027154245514588227E+00 -.15747189245191811624E+00

.28057893569497588393E+00 -.51385824959018266966E+00

-.25021825677523462650E+00 .92563380408403694855E+00

.39119485848022078622E+00 .74035326115954227383E-02

-.53883013789247907049E-02 .23732714701433562515E-02

.28216843359523130497E+00 .73827578832658191654E+00

.69001463666172697595E-02 -.76159368867617874971E-02

.57867270439665259227E-02 -.41923034942253044333E-02

.15317510793683363716E-02

-.95880640425136776095E-01 -.40344092518485091814E+00

.64467008737306663591E+00 .51178827806905180520E+00

-.88369713485047726720E-02 .56564591894384414622E-02

-.18444260385135132635E-02

.34922596086193184728E-01 .16638966212550704293E+00

-.28757341691872739808E+00 -.29477666342253877607E+00

.55488163080471664124E+00 .55487478541719581493E+00

.95407974534744366402E-01

.17109643606143040695E+00 .56793337432045976065E+00

.33765492630656679385E+00 -.16573826777149666071E-02

.12369397459480143461E-02 -.44654930558792391279E-03

0/

END

FIT

8P 5.59

8P 3.97

9P 3.18

9P 2.30

7D 6.39

8D 1.96

7G 4.87

7G 2.12

END

**Tellurium (TZP, 4d frozen)**

BASIS

1S 34.100

2S 22.950

3S 10.250

4S 7.150

2P 23.550

3P 11.466

4P 6.350

3D 13.100

4D 4.300

5S 3.600

5S 2.350

5S 1.580

5P 2.950

5P 1.800

5P 1.110

END

CORE 4 3 2 0

1S 381.000

1S 94.600

1S 52.800

2S 60.400

2S 22.650

3S 11.950

3S 8.000

4S 8.200

4S 5.200

5S 3.600

5S 2.350

5S 1.580

2P 34.200

2P 21.800

3P 12.900

3P 9.650

4P 6.750

4P 4.400

5P 2.950

5P 1.800

5P 1.110

3D 21.100

3D 12.750

3D 8.700

4D 6.500

4D 4.100

4D 2.650

END

DESCRIPTION

.10052581756842021016E-01 .81750053731865751638E-01

.93586109018726992836E+00 -.13485320493029114355E-01

.10697040855614193752E-02 -.26513510294778278725E-02

.56550585131723719173E-02 -.41702715840658812971E-02

.73658162600256634010E-03 -.32148549485825240069E-03

.18206410303958092006E-03 -.63009502819828626646E-04

-.28439449164092073588E-02 -.91175239097539453881E-01

-.15890281584805029946E+00 -.20517187557373303686E+00

.11171367225270321111E+01 .15694290368288284047E-02

-.65679019212370199493E-02 .73719755335496404622E-02

-.27453114862408850745E-02 .15195558712322193053E-02

-.94344163446667900566E-03 .34045943309301987621E-03

.85190159918388411002E-03 .85969002200438141004E-01

-.32392756750281470612E-01 .18857777425666308324E+00

-.67155895618924454915E+00 .95952423186044655257E+00

.47401197060487648693E+00 -.33746119121760170811E+00

.73077854792181817789E-01 -.36094780636332875201E-01

.21701914090852477551E-01 -.77562666868240277146E-02

-.35273710543100528312E-03 -.44143869940757272941E-01

.25333927365388076575E-01 -.96740045674605285031E-01

.32713447475042756185E+00 -.31182791698978923334E+00

-.12260420388480748333E+01 .15451064220486794287E+01

.52364360645797136673E+00 .20899789660940311375E-01

-.13531655500324066971E-01 .55150406568119647588E-02

.23193734225489223699E+00 .78800800605836229806E+00

.71962989366207213635E-02 -.76307142131315603903E-02

.48615896266727106137E-02 -.31391035571894311713E-02

.14609472203062075051E-02 -.78920702070223126487E-03

.28261791001494136711E-03

-.79472838212686969239E-01 -.44801543063899779140E+00

.72229986509087884539E+00 .43663152828049672793E+00

-.93671180023419640948E-02 .56695111277565738914E-02

-.25578546706845266094E-02 .13825705806231698355E-02

-.49746716919681977561E-03

.30157990290238610026E-01 .21221744568772898942E+00

-.38573739908210091798E+00 -.28948079325552983354E+00

.81886933897556424267E+00 .40073438367870678478E+00

.25317590375166830821E-02 -.11727572800912997291E-02

.59824683196785786478E-03

.12601832540779023262E+00 .58525459727608208915E+00

.34749459005875854567E+00 -.91933097041635978598E-03

.36875188876547409242E-03 .29475135659298364754E-05

-.48787502867246833327E-01 -.26499407910941036315E+00

-.96336136317368786797E-01 .49781278283387331296E+00

.53018060342653694494E+00 .13056894963797002762E+00

0/

END

FIT

8S 4.30

8P 4.38

9P 2.69

8D 3.98

END

**Titanium (TZP, 3p frozen)**

BASIS

1S 15.05

2S 8.50

3S 3.75

2P 8.30

3P 3.15

3D 1.04

3D 2.30

3D 4.95

4S 0.75

4S 1.20

4S 2.00

4P 1.20

END

CORE 3 2 0 0

1S 26.3000

1S 20.1000

2S 8.3000

2S 5.6000

3S 4.6500

3S 2.9500

4S 0.8000

4S 1.2000

4S 1.9000

2P 12.0000

2P 7.3500

3P 4.0500

3P 2.4500

END

DESCRIPTION

.30008547379925964993E+00 .70179108260469547087E+00

.30971873637311915506E-01 -.41260295262091815915E-01

.26631203707239693018E-01 -.12123618030042330670E-01

.13189464097120029169E-02 -.35316410045338913516E-02

.53564922471576546098E-02

.74701837744269070085E-01 -.44816422778876158217E+00

.10259744011727984780E+01 .81737696996568062757E-01

-.33125817694371451072E-01 .19374549469598472545E-01

-.27789458580917275610E-02 .72133251242859882171E-02

-.10239625397086822220E-01

-.39370745209337490444E-01 .18352080745991675159E+00

-.31516163994103868484E+00 -.36451586076848668805E+00

.83799112261317898476E+00 .47800315725373393461E+00

.43339957375633847078E-02 -.10151355979008853947E-01

.13360502125084720154E-01

.35432668149125512436E+00 .67740730085329892152E+00

.24309502812947434390E-02 -.72096371139482773229E-03

-.12161933765827573350E+00 -.27151736104139262418E+00

.70203819767983177691E+00 .42399036210717361284E+00

0/

END

FIT

7S 2.70

6P 5.05

6P 3.03

7P 2.15

4D 13.07

5D 8.72

6D 3.47

7D 2.40

6F 2.24

5G 8.40

5G 4.39

5G 2.29

END

**Thallium (TZP, 5p frozen)**

BASIS

1S 59.000

2S 37.600

3S 21.800

4S 12.850

5S 7.400

2P 39.400

3P 19.450

4P 12.550

5P 6.600

3D 22.950

4D 11.500

4F 8.600

1S 192.400

6S 3.500

6S 2.250

6S 1.460

6P 2.550

6P 1.450

6P .830

5D 5.650

5D 3.350

5D 1.950

END

CORE 5 4 2 1

1S 800.000

1S 192.400

1S 90.700

2S 103.700

2S 39.550

3S 50.350

3S 21.500

4S 13.150

4S 9.300

5S 7.450

5S 5.000

6S 3.500

6S 2.250

6S 1.460

2P 68.700

2P 37.700

3P 21.450

3P 15.700

4P 14.200

4P 9.700

5P 6.400

5P 4.050

6P 2.550

6P 1.450

6P .830

3D 28.900

3D 18.500

4D 12.750

4D 8.250

5D 5.650

5D 3.350

5D 1.950

4F 17.000

4F 9.600

4F 5.500

END

DESCRIPTION

.23122680865530785554E-01 .14825083471866848384E+00

.86290423624312750928E+00 .13170810020580264732E-01

-.34428433496231411470E-01 .30029830404127207805E-01

.11796980194425466058E-02 -.83969382759040503156E-03

.81913173825133318157E-03 -.46476999088292467047E-03

.20657760695280330868E-03 -.84833109698123041224E-04

.42253558668836530790E-04 -.14404920467234493187E-04

-.78228633727171134615E-02 -.58561940520297152413E-01

-.27202255786361168033E+00 -.85912775449381056925E-01

.99806762748739874525E+00 .96405037732803858241E-01

.15183013371355060395E-02 .36222889721473075161E-02

-.50997946113385999103E-02 .36902867911909357709E-02

-.19162676750893064322E-02 .86560632625473990509E-03

-.45008802183333902844E-03 .15642352871633272171E-03

.38084011190565032583E-02 .17799902829103451951E-01

.15604062690026879179E+00 -.63780060507440308054E-02

-.32215882201990708733E+00 -.36623398511311461290E+00

.11939014202796269792E+01 -.43755688928904436924E-02

.52686914957316040298E-02 -.13158474628267335157E-02

-.14417431625845240190E-03 .28530240532934579827E-03

-.19846328466446559757E-03 .76669708709613354990E-04

-.20079173409171132379E-02 -.37996613624566560191E-02

-.94542474780451510319E-01 .28432090342757350421E-01

.73352710273850166289E-01 .30985305906423682432E+00

-.88446276150338520861E+00 .10681294341617733057E+01

.21222076961852809340E+00 -.18382850578600843511E-01

.88724746113065088710E-02 -.46496027228229057718E-02

.26333350585659442977E-02 -.95567684031563871708E-03

.93669898855973561264E-03 .80581455903417330278E-03

.46229007153449630518E-01 -.17590801928209557486E-01

-.17421473123850627490E-01 -.16338177552860852493E+00

.44643881310022276443E+00 -.69487497688729271683E+00

-.87574032412267105441E-01 .86553274288133064118E+00

.35058485875756933980E+00 .60697845005097568472E-02

-.29189379196952773207E-02 .12824737763504890069E-02

.15001745003664412881E+00 .87505093010143153442E+00

.99025497796483424873E-02 -.18909740253514727842E-01

.16618523814470469863E-01 -.73707560797423559568E-02

.27562178552137774415E-02 -.13779730438564896221E-02

.52394486408086939338E-03 -.24751118002164376004E-03

.91065408342824095858E-04

-.58294438356787291189E-01 -.51216415992275499125E+00

.97521230267518155266E+00 .23967688471157422292E+00

-.84333898459315659313E-01 .31035334235696543370E-01

-.11148201238920938533E-01 .55487650178465131034E-02

-.21147346737583456143E-02 .10015025544824630171E-02

-.36897702948394077568E-03

.25972084420017776951E-01 .26779698033154458736E+00

-.49255069315241023498E+00 -.54177139080710445818E+00

.96652273576977032477E+00 .53750289651737204188E+00

.12038170406975256393E-01 -.57195933782538599041E-02

.23801653026190357915E-02 -.11710482917507212923E-02

.43783324535847544705E-03

-.11315793528191250492E-01 -.11376547262531870597E+00

.16387526086953482785E+00 .38419722376756065829E+00

-.66681153592142927700E+00 -.16882667199010259473E+00

.81656818925864482761E+00 .33952476629482275516E+00

-.33981758640992923599E-02 .26703137370130123653E-02

-.10085164110925698075E-02

.37333283400794770035E+00 .66774530117197639090E+00

-.61647473827544548020E-02 .44968993671615394014E-02

-.12092918242381925972E-02 .37794241623585609268E-03

-.10192196420115086266E-03

-.19874993789947756340E+00 -.35391199917789878393E+00

.71518595572476839628E+00 .47534920978135936087E+00

-.43430457910395084409E-02 .30951879768057865422E-02

-.96631238076142313750E-03

.27530392799226233169E+00 .61744170753804972218E+00

.24331278279565243805E+00

0/

END

FIT

10S 3.19

10P 4.89

10P 3.66

11P 3.02

10D 4.62

END

**Vanadium (TZP, 3p frozen)**

BASIS

1S 15.10

2S 9.10

3S 3.95

2P 8.79

3P 3.37

3D 1.14

3D 2.50

3D 5.30

4S 0.80

4S 1.30

4S 2.20

4P 1.26

END

CORE 3 2 0 0

1S 28.5500

1S 21.2500

2S 8.7500

2S 6.2000

3S 5.0500

3S 3.1500

4S 0.8400

4S 1.2800

4S 2.0500

2P 12.4000

2P 7.7500

3P 4.4000

3P 2.6500

END

DESCRIPTION

.24261946942472378819E+00 .75983815749099559156E+00

.31951933863151538162E-01 -.41021387260947665687E-01

.23353794768155092254E-01 -.10511269882997416672E-01

.11049618970386308834E-02 -.29886568364417753427E-02

.47207446257881736928E-02

.71818191709305909121E-01 -.44625002072093544747E+00

.99858025297661823227E+00 .11781255649935576391E+00

-.51843544217613828085E-01 .28985480334837505911E-01

-.37728781992534699691E-02 .99473526554696330149E-02

-.14892443725125388554E-01

-.35853008528408412958E-01 .18088555526905833459E+00

-.26157301461606785598E+00 -.43955657797707936574E+00

.82967321973580809846E+00 .50997187791545617319E+00

.29294917456765528357E-02 -.64907561722867233855E-02

.81829302290920315210E-02

.37532475578407753503E+00 .65393667814564615526E+00

.46153499560086519438E-02 -.20908354356248891152E-02

-.12789923634957298115E+00 -.27521098982472597116E+00

.68059864336456143974E+00 .45268181332175971843E+00

0/

END

FIT

6S 6.25

7S 2.15

5P 7.60

6P 3.14

7P 2.21

5D 9.01

6D 6.16

6D 3.63

END

**Tungsten (TZP, 4f frozen)**

BASIS

1S 52.000

2S 33.250

3S 19.100

4S 11.650

2P 36.850

3P 16.500

4P 11.550

3D 20.100

4D 10.300

4F 6.200

1S 163.900

5S 6.050

5S 3.950

6S 2.850

6S 1.800

6S 1.140

5P 5.900

5P 4.000

5P 2.700

5D 4.150

5D 2.250

5D 1.200

6P 1.800

END

CORE 4 3 2 1

1S 669.000

1S 163.900

1S 80.500

2S 89.000

2S 35.150

3S 44.850

3S 18.900

4S 11.600

4S 8.200

5S 6.050

5S 3.950

6S 2.850

6S 1.800

6S 1.140

2P 59.200

2P 33.750

3P 19.250

3P 14.350

4P 12.150

4P 8.150

5P 5.150

5P 3.150

3D 25.750

3D 16.350

4D 11.000

4D 6.800

5D 4.150

5D 2.250

5D 1.200

4F 13.550

4F 7.050

4F 3.550

END

DESCRIPTION

.19439168067705871884E-01 .12648558256383402032E+00

.88946598912864816011E+00 -.18316760854811852451E-02

-.66945730502543081969E-02 .61030577578422076646E-02

.28813100145131721221E-03 -.28567308064260068428E-03

.27729496708100179149E-03 -.16029257378988642974E-03

.92866849977973314864E-04 -.45894626475096101563E-04

.21894163235689672112E-04 -.74822715540226567184E-05

-.65125092613020239482E-02 -.49612796783309778825E-01

-.28288058068779786680E+00 -.87893932987690173708E-01

.98064002634992308849E+00 .11781346125284808402E+00

.40666726359722587050E-02 .17203355868143838971E-03

-.91221947021306144191E-03 .84270057559247336151E-03

-.57612129966150391232E-03 .30800922898181282497E-03

-.15247542873090847446E-03 .52919729386954093375E-04

.31608184026980542906E-02 .12657735211620899443E-01

.16048358036368481905E+00 -.10554824838370965509E-01

-.28047546108561061118E+00 -.39766423009926815668E+00

.11911628609251831978E+01 -.16550854692074214169E-01

.16115098298314780439E-01 -.76526669582423411872E-02

.38896431347455184824E-02 -.17768410213571772353E-02

.81429859014040862925E-03 -.27367170361956155711E-03

-.16466331641506023933E-02 -.82014153455309225446E-03

-.95297957979076586277E-01 .32390561223870489860E-01

.38540116242778539590E-01 .32912831329857367146E+00

-.86216634420278037698E+00 .10304708352641400726E+01

.23774962458566425982E+00 -.24032293925210180749E-02

.13565833166448233900E-02 -.10478015917846823530E-02

.62569388028857789944E-03 -.23540029718305589021E-03

.15787365325066080035E+00 .86502973437661867528E+00

.82203926035462997202E-02 -.12355055155817780757E-01

.95049718216334254384E-02 -.42957729149795129478E-02

.13525766109412510133E-02 -.50510297706318600403E-03

-.58316064794245975333E-01 -.50662937684216136081E+00

.95761240635674893529E+00 .22998894627694854709E+00

-.44227534772973517430E-01 .17036651069470221120E-01

-.52185763026864244798E-02 .19522864831548049185E-02

.24698420041008261466E-01 .26026748328251675391E+00

-.50490226303695162624E+00 -.40428794571900095178E+00

.90879784848322253676E+00 .48472228716600429532E+00

.46377131384472015246E-02 -.15602247300151360441E-02

.38466951617934597163E+00 .65791541136983877625E+00

-.52978900132579914706E-02 .40612319688147300450E-02

-.12317279968200886640E-02 .52195074934742935122E-03

-.17986996646397954689E-03

-.19953310766988169300E+00 -.32269546988859715286E+00

.73648874142071463211E+00 .44686451313483810388E+00

-.39458612543603966208E-02 .29298023155029315201E-02

-.10591518595634494271E-02

.35427446605529550050E+00 .60792788380476769916E+00

.22392054659764198954E+00

0/

END

FIT

10S 4.16

9P 6.28

9P 4.71

10P 3.94

10P 3.00

9D 4.25

END

**Xenon (TZP, 4d frozen)**

BASIS

1S 35.100

2S 24.100

3S 10.500

4S 7.650

2P 24.650

3P 11.950

4P 6.800

3D 13.800

4D 4.850

5S 4.000

5S 2.650

5S 1.800

5P 3.300

5P 2.050

5P 1.290

END

CORE 4 3 2 0

1S 397.000

1S 99.300

1S 55.100

2S 62.700

2S 23.700

3S 12.550

3S 9.000

4S 8.950

4S 5.700

5S 4.000

5S 2.650

5S 1.800

2P 36.100

2P 22.850

3P 13.450

3P 10.150

4P 7.250

4P 4.850

5P 3.300

5P 2.050

5P 1.290

3D 22.350

3D 13.550

3D 9.300

4D 7.000

4D 4.600

4D 3.100

END

DESCRIPTION

.10983365024654462641E-01 .85361377576659347444E-01

.93219312957464539604E+00 -.13264399092658118659E-01

.12123432675947445978E-02 -.38930986057541202595E-02

.72775494896917597085E-02 -.48381837919929376185E-02

.97869279944408815904E-03 -.43288248107404902554E-03

.24404937265383084243E-03 -.82373491505435112348E-04

-.31743258162799021550E-02 -.86163502907286262933E-01

-.17242137027144713302E+00 -.19676347810784913794E+00

.11178930755545111175E+01 .47432547187749533185E-02

-.13055854023630904942E-01 .11928335547841104058E-01

-.41262394056821279434E-02 .22341714830734380956E-02

-.13668927135254789126E-02 .47962651008130318276E-03

.10094407945923902694E-02 .80800153042463088160E-01

-.19222849169432211502E-01 .18181186107863223089E+00

-.67668992159176655221E+00 .89636106778558555241E+00

.54936797754690358531E+00 -.36345945211390617846E+00

.91641841386995229191E-01 -.45433938905055544499E-01

.27119782916232289954E-01 -.94531381793859555612E-02

-.43647848811085334904E-03 -.41862773783180727560E-01

.18235499257016091201E-01 -.94316427069244285208E-01

.33500913973040818972E+00 -.18428086882514074785E+00

-.13757841285270906440E+01 .15088310813888707873E+01

.56503535292488116237E+00 .28356041218256723691E-01

-.17589966214370283959E-01 .68929065077120979174E-02

.22242845427902901978E+00 .79717587613884421938E+00

.93980175980838305538E-02 -.10302133756854915322E-01

.67293316794571917533E-02 -.43977652873620010424E-02

.19994828198748797014E-02 -.10804761282775766310E-02

.38143304995284771754E-03

-.76435070062040449956E-01 -.45570876469293247757E+00

.74323400815508700035E+00 .41767017383370796946E+00

-.12990825226682172280E-01 .79812480684777007550E-02

-.35719274042619510862E-02 .19444378577263572022E-02

-.69199507860719989584E-03

.29739636318004993715E-01 .22058320931766511874E+00

-.39863532797795420137E+00 -.30886712411221645125E+00

.82214084418141508692E+00 .41056520841475474537E+00

.70983625663561212768E-02 -.34550902191491776085E-02

.14171667745323285499E-02

.11742707244899062979E+00 .58047407340872447801E+00

.35930477894551782203E+00 -.26303115626953718284E-02

.11118220497193054361E-02 -.21054935506827136783E-03

-.47507227507844494718E-01 -.27886949847813152781E+00

-.10974906225128289172E+00 .50332301783438238019E+00

.52008505643408953212E+00 .13112656974623465578E+00

0/

END

FIT

9S 3.76

7P 8.13

8P 4.96

9P 4.12

9P 3.09

8D 4.54

9D 3.60

8F 5.91

8F 3.80

8G 3.29

END

**Yttrium (TZP, 4p frozen)**

BASIS

1S 28.200

2S 16.300

3S 7.800

4S 4.150

2P 17.100

3P 7.900

4P 3.000

3D 9.100

5S 1.850

5S 1.200

5S .810

4D 2.750

4D 1.400

4D .750

5P 1.200

END

CORE 4 3 1 0

1S 240.000

1S 59.500

1S 38.200

2S 45.000

2S 16.100

3S 8.300

3S 5.750

4S 4.300

4S 2.800

5S 1.850

5S 1.200

5S .810

2P 23.400

2P 15.250

3P 9.200

3P 6.200

4P 4.300

4P 2.850

4P 1.950

3D 13.500

3D 7.850

3D 4.850

4D 2.750

4D 1.400

4D .750

END

DESCRIPTION

.69074529488362696600E-02 .61220895293084991173E-01

.97875374075892174197E+00 -.41984097847513578150E-01

.36928504274347625147E-03 -.65208325343577401914E-03

.77169784901476930379E-03 -.50226142985851721101E-03

.28293813334253275571E-03 -.14048642181595244341E-03

.92046910857030228375E-04 -.34447938952982162634E-04

-.16121087227823113330E-02 -.30681361228578363232E+00

.23943206774371303402E+00 -.39774172885137626388E+00

.11069950428663037911E+01 -.48772149650486993147E-02

.70633419451023556415E-02 -.40263328062186956244E-02

.20177164048468831416E-02 -.91285805362365359319E-03

.57598484620558160554E-03 -.21180031777456978558E-03

.21020681666873122957E-03 .29322745164089447645E+00

-.41546877766767204010E+00 .36291431069843327339E+00

-.60969945778209200249E+00 .97527123572494744952E+00

.19303055180845701821E+00 -.22938078474878876439E-01

.13589266542571257906E-01 -.77304084800984803130E-02

.53895403444891827952E-02 -.20819968752052147448E-02

-.29250875531752271169E-04 -.13288266574766999240E+00

.19721588604215928298E+00 -.16331531865965898120E+00

.25146734926826930234E+00 -.46637708283908485551E+00

-.12880692233240600975E+00 .80383655077182714077E+00

.38089837161838668855E+00 .92956148304364442231E-02

-.68091687943668301186E-02 .29956670162945807367E-02

.29655176766615898298E+00 .72556276535982633380E+00

.29362792241401348102E-02 -.31001474725773328807E-02

.29339769139675934799E-02 -.23731010236901408511E-02

.91470948193807526290E-03

-.10025389439538948122E+00 -.38102746930447145557E+00

.64088902134372793817E+00 .51202912413620760290E+00

-.48434590985881806335E-02 .35881280027503861807E-02

-.11097821882143199101E-02

.33085779104446343124E-01 .14162347575589648296E+00

-.26539750033002773577E+00 -.22876590225980203419E+00

.48451734661378215163E+00 .55771425434383148367E+00

.12682401484528149638E+00

.19500219381720268474E+00 .57705963331077203549E+00

.31932122333175461115E+00 -.13740062902512354716E-02

.11165792130879194106E-02 -.44582747874180014879E-03

0/

END

FIT

8S 4.24

8P 4.94

8P 3.49

9P 2.01

7D 3.35

8D 2.42

7F 2.15

7G 3.69

END

**Zinc (TZP, 3p frozen)**

BASIS

1S 17.45

2S 12.70

3S 5.60

2P 12.19

3P 4.77

3D 1.68

3D 3.70

3D 7.70

4S 0.90

4S 1.50

4S 2.60

4P 1.54

END

CORE 3 2 0 0

1S 35.2500

1S 27.8500

2S 11.8000

2S 6.6000

3S 7.2000

3S 4.3500

4S 1.0000

4S 1.6000

4S 2.7000

2P 16.6500

2P 10.9000

3P 6.3000

3P 3.7000

END

DESCRIPTION

.38450382214131723080E+00 .61193102536368970679E+00

.74317625043614096714E-01 -.16886177764765969433E+00

.13148610639488389484E+00 -.18443848494372944158E-01

.16266012371755204143E-02 -.42939719136489808496E-02

.71715571820622457666E-02

.94048379195171255551E-01 -.49015848572219911095E+00

.10775001860801711739E+01 .72844789563030976076E-01

-.73506175914250543313E-01 .21858127822301946785E-01

-.25228779190250767185E-02 .64853338970496535523E-02

-.10175304993821803964E-01

-.52605248041694918637E-01 .21174477552669906943E+00

-.28702368307148040660E+00 -.86533325605373345724E+00

.13038803750211729326E+01 .51359635422401428428E+00

.16996270752170511160E-02 -.35597899309880130791E-02

.45515121884775154582E-02

.37087030303008677867E+00 .65251542971064502296E+00

.47431293744281000785E-02 -.20874877346855145316E-02

-.12749938537217453405E+00 -.29826990480414483642E+00

.71119972645495177499E+00 .43742941743092716989E+00

0/

END

FIT

5S 11.86

6S 10.03

6S 7.26

7S 2.43

5P 9.60

6P 6.61

6P 3.92

7P 2.74

5F 11.35

END

**Zirconium (TZP, 4p frozen)**

BASIS

1S 26.800

2S 16.700

3S 8.200

4S 4.300

2P 17.500

3P 8.250

4P 3.200

3D 9.250

5S 2.050

5S 1.300

5S .850

4D 3.100

4D 1.650

4D .900

5P 1.300

END

CORE 4 3 1 0

1S 249.000

1S 62.000

1S 39.300

2S 46.000

2S 16.600

3S 8.600

3S 6.050

4S 4.550

4S 3.000

5S 2.050

5S 1.300

5S .850

2P 24.200

2P 15.750

3P 9.550

3P 6.500

4P 4.450

4P 3.000

4P 2.100

3D 14.150

3D 8.250

3D 5.150

4D 3.100

4D 1.650

4D .900

END

DESCRIPTION

.71399916387992947570E-02 .67487984025194763804E-01

.96499777164117217509E+00 -.33388536035847946049E-01

.65198015505943114449E-03 -.10515764755610276533E-02

.12362170016573807881E-02 -.79558776110635269063E-03

.45509130650948724896E-03 -.21016658039582506237E-03

.11932203777753981123E-03 -.42810150912927477694E-04

-.17304766594331227967E-02 -.24380297156637714684E+00

.12537072010514627163E+00 -.34243753727221093452E+00

.11098029559142246203E+01 -.53791774599499155712E-02

.78558549147447979261E-02 -.45261692982473018809E-02

.23274028822263424440E-02 -.98496822821419072885E-03

.53676618459422601685E-03 -.18901492671474497088E-03

.25825182172364619018E-03 .23752389712214591611E+00

-.31478797147492187936E+00 .31559365059916671958E+00

-.61849763965228443663E+00 .96703215915504836531E+00

.20707862529012777131E+00 -.31487909150411511061E-01

.19688203379743633714E-01 -.10354457467166996998E-01

.62808693921099535695E-02 -.23291368726475717051E-02

-.48350433359316777619E-04 -.11050723439782289548E+00

.15583170367127471834E+00 -.14562105529921440250E+00

.26183415614485805456E+00 -.46838848859930143353E+00

-.15978626926569933220E+00 .81990253058388051777E+00

.37877331980003364764E+00 .16894989763187710091E-01

-.10074906488731610529E-01 .40782682834329831573E-02

.28902858454930513421E+00 .73288235969205339781E+00

.28883350447610070880E-02 -.30327178081729975860E-02

.30360395190770854933E-02 -.26225285164007522930E-02

.10304375211589802703E-02

-.97134068255364788835E-01 -.38974455283752018619E+00

.62932898855375929426E+00 .52568555643993997162E+00

-.74864412389706643663E-02 .59149917341676846311E-02

-.20241344041079353984E-02

.32781660531070205833E-01 .15059726183751392936E+00

-.27247978469023798054E+00 -.24238386964333147322E+00

.53091734215621066983E+00 .51653699534864949694E+00

.12404868471375053718E+00

.18402219899026506233E+00 .58231358063870708452E+00

.32091541848266014192E+00 -.17056861737095446729E-02

.11409650230559472035E-02 -.41315981689188027715E-03

0/

END

FIT

5S 15.47

7S 6.01

8S 4.36

8S 3.50

9S 2.09

7P 6.48

9P 2.96

9P 2.15

5D 17.77

6D 12.73

6D 7.85

7D 5.70

7D 3.63

8D 2.67

8D 1.75

7F 2.30

7G 1.80

END
